# A novel application of the Staudinger ligation to access neutral cyclic di-nucleotide analog precursors *via* a divergent method†
†Dedicated to Prof. Al Padwa, a scholar and a sportsman who has reached pinnacles in both science and sport, on the occasion of his 80^th^ birthday.
‡
‡Electronic supplementary information (ESI) available. See DOI: 10.1039/c7ra06045a



**DOI:** 10.1039/c7ra06045a

**Published:** 2017-06-07

**Authors:** M. H. Fletcher, C. E. Burns-Lynch, K. W. Knouse, L. T. Abraham, C. W. DeBrosse, W. M. Wuest

**Affiliations:** a Department of Chemistry, Temple University, Philadelphia, PA 19122, USA; b Department of Chemistry, Emory University, Atlanta, GA 30322, USA. Email: wwuest@emory.edu

## Abstract

Our efforts to develop a scalable and divergent synthesis of cyclic di-nucleotide analog precursors have resulted in (1) an orthogonally protected di-amino carbohydrate as well as (2) the novel application of the Staudinger ligation to provide medium-sized macrocycles featuring carbamate or urea linkages.

## Abstract 2

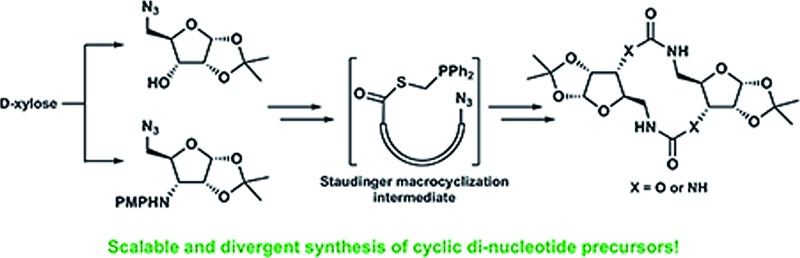
Herein we present a scalable and divergent synthesis of cyclic di-nucleotide analog precursors facilitated by differentiated di-amino sugars and a Staudinger ligation to provide medium-sized macrocycles featuring carbamate or urea linkages.

## 

Natural products have been implicated in a variety of bacterial survival processes. While compounds such as *N*-acyl homoserine lactones (AHLs), auto-inducers (AIs), and the *Pseudomonas* quinolone signal (PQS) allow for external bacterial communication and coordination, second messenger molecules such as cyclic mono- and di-nucleotides provide internal regulation and organization of proteins and their downstream targets (Fig. 1).^1–5^ These second messenger molecules are synthesized in response to environmental triggers such as changes in oxygen and amino acid concentrations.^6–8^ In the thirty years since Benziman discovered that c-di-GMP regulates cellulose synthesis in *Acetobacter xylinum*, many others have demonstrated that cyclic di-nucleotides (CDNs) also regulate motility, biofilm formation, fatty acid synthesis, cell wall homeostasis, and more (Fig. 1).^5,9^ The ubiquity of CDNs as ligands of bacterial signaling pathways stems from their inherent flexibility which allows them to target many disparate entities. The phosphate linkages allow the integral twelve-membered macrocycle to adopt a breadth of conformations, displaying the nucleobases across an expanse of chemical space (Fig. 2). Many crystal structures demonstrate this polymorphism, as c-di-GMP and c-di-AMP bind to proteins and riboswitches in both the open and closed forms, as well as intercalated dimers.^10–14^ The development of conformationally biased CDN analogs is essential for selectively inhibiting and probing these bacterial signaling targets.

**Fig. 1:**
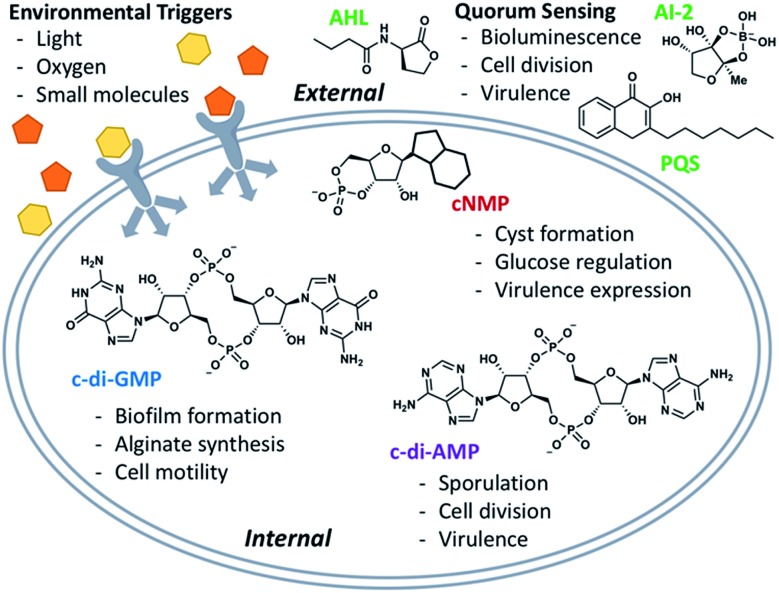
Overview of signaling systems externally (quorum sensing) and internally (second messenger molecules).

**Fig. 2:**
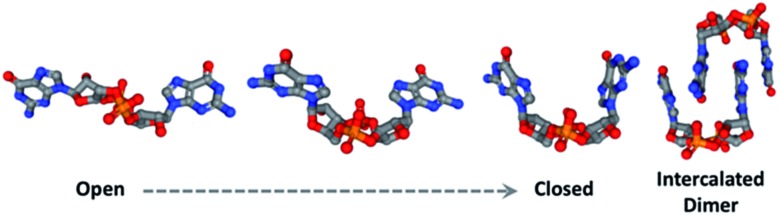
c-di-GMP takes on an array of conformations as observed in these structures taken from crystal structures (PDB IDs from left to right: 5MF5, ; 5HTL, ; 4RT1, ; 5EXX).^16^

Pioneering work has been done to discover inhibitors of these signaling systems, which has led to promising nucleotide and non-nucleotide based molecules. A recent review by Sintim *et al.* provides an excellent overview of how both the native CDN ligands and other reported analogs interact *in vitro* with various signaling targets in both Gram-positive and Gram-negative bacteria.^5^ However to the best of our knowledge, there have not been any *in vivo* applications with CDN analogs. This is likely due to both the polarity and hydrolytic instability of the phosphate groups, and the propensity for aggregation due to the extensive hydrogen bonding donors and acceptors. To correct for the lack of probe molecules, Strobel has called for “designing c-di-GMP analogs that tightly bind second messenger targets but simultaneously incorporate modifications that impart desirable properties for *in vivo* studies, such as increased cell permeability and increased resistance to enzymatic degradation, is a desirable goal”.^15^ Therefore, while the phosphate group plays a key role in the flexibility of CDNs, it also a primary reason why suitable tool compounds have not yet been realized. Thus, a chief strategy for CDN analog development has been to substitute the phosphate moiety for neutral non-hydrolyzable linkages. Any effective *in vivo* analogs must straddle the border between hydrophobicity and hydrophilicity to allow for acceptable solubility in water as well as maintain the ability to cross the bacterial membrane.

Typically, non-phosphate analogs are formed by joining two equivalents of a common bis-substituted nucleoside, which in turn comes from the commercially available nucleoside (Fig. 3A).^17^ Some synthetic efforts have been undertaken to develop non-phosphate containing CDNs.^17–19^ Successful endeavours by Jones and Isobe, have yielded c-di-GMP analogs featuring urea, thiourea, carbodiimide and triazole connections.^17,18^ Additionally, carbamate analogs lacking the 2′-hydroxyl group were reported by Miller.^19^

**Fig. 3:**
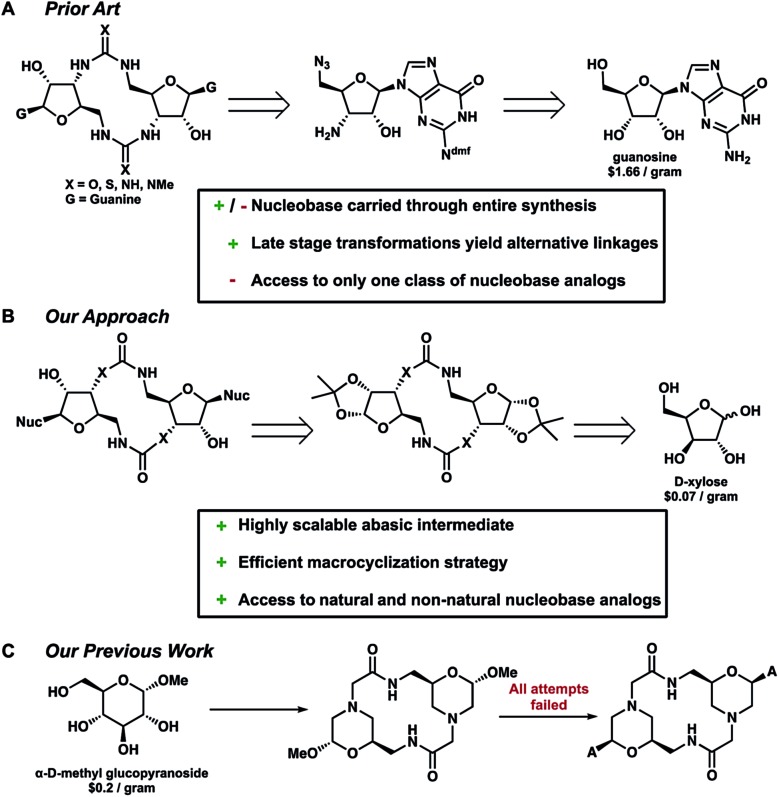
Retrosynthesis of (A) analogs by Jones (B) our current work and (C) our previous morpholinoside work.

However, these routes all share a few major limitations. No extensive work has been done to incorporate both linkage and nucleobase substitutions in a synthetic design. To date, only the triazole analogs developed by Isobe *et al.* have featured a nucleobase other than guanine which were accessed *via* the respective adenosine and guanosine substituted monomers. Following these methods, to facilitate access to other CDNs, the entire sequence would need to be repeated and optimized. Therefore, these routes limit analog design to those that rely on abundant and relatively inexpensive nucleoside precursors. Additionally, there is no certainty that the previously reported methods would be amenable to such a course of action.

With these limitations and Strobel's inspiration in mind, we set out to develop a divergent strategy that would provide late-stage abasic macrocyclic intermediates featuring neutral non-phosphate linkages. This could then be reacted with an array of natural and non-natural nucleobases and other heterocycles expanding the available targets for biological study (Fig. 3B). Previous work by Giese reaffirmed our hypothesis providing access to the natural c-di-GMP and c-di-AMP ligands *via* a similar rationale.^20^ Efforts in our lab set out under a similar approach with a morpholinoside-based backbone, however, all attempts to install the adenine nucleobase failed (Fig. 3C).^21^ This was due in part to both the reactivity of the anomeric leaving group as well as the tertiary amine within the scaffold. With these previous reports in mind, we set out to develop analogs from readily available, inexpensive carbohydrate starting materials.

Although Jones and Miller have previously reported the urea and carbamate analogs, no biological studies were reported, possibly due to limited amounts of compound. To circumvent similar short-comings we sought to develop an approach to afford gram quantities of late stage intermediates for the further development of this chemistry. Continuing our investigation not only allowed us to develop a novel method for the macrocyclization but by developing an orthogonally protected bis-amino carbohydrate we could also provide other di-amino-based scaffolds which are currently being developed in our laboratory.

Following previously reported chemistry, azide ketone **1** was prepared from d-xylose in four steps (Scheme 1).^22,23^ Using this common intermediate we could provide both the *N*,*O*- and *N*,*N*-substituted glycosides *via* minimal transformations. Reduction of the ketone afforded compound **2**, which could then be protected with *para*-methoxylbenzyl chloride and subsequently treated with RANEY® nickel under hydrogen atmosphere yielded the free amine **4**.^23^ Similarly, reductive amination conditions using either PMB-amine or *para*-methoxylphenylamine yielded the orthogonally protected glycosamines **5a** or **5b**. While both products were formed efficiently, deprotection to yield the free 3′-amine **6** was less effective with the PMB protecting group. Initial attempts with DDQ were unsuccessful, while CAN provided the amine in 42% yield. Deprotection of compound **5b** with CAN gave the amine in variable yields (54–75%) however, the use of trichloroisocyanuric acid (TCCA) provided the product reliably in greater than 70% yield.^24^ Additionally, following workup, deprotection with TCCA required no further purification. Finally, treatment of azide **5b** with Pd/C under hydrogen atmosphere revealed the 5′-amine **7** in high yield. Next, each linear bis-glycoside **11a** or **11b** was formed *via* carbonyldiimidazole coupling (CDI) (Scheme 2). The 3′-alcohol **2** or 3′-amine **6** was coupled to CDI to introduce the obligatory carbonyl linkage to afford **8a** and **8b** respectively. The requisite 5′-amino coupling partner **4** or **7** was next introduced to generate the carbamate-linked compound **9a** or the urea-linked compound **9b** each in greater than 90% yield. Following the successful carbohydrate coupling, the deprotection of the PMB-protected ether **9a** using DDQ or the PMP-protected amine **9b** using TCCA yielded compounds **10a** and **10b** ready for the second CDI coupling. Treatment with CDI yielded the necessary linear precursors **11a** and **11b** for macrocyclization in high yield. Any attempts to use less than two equivalents of CDI afford a dimerized tetra-glycoside byproduct, which made purification difficult.

**Scheme 1:**
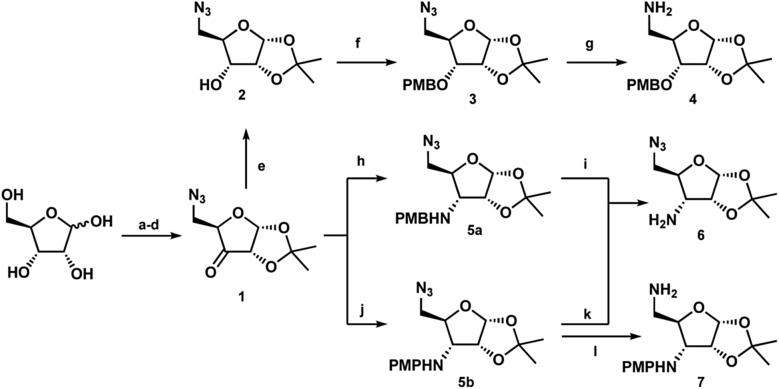
Divergent synthesis to access both *N*,*O*- and *N*,*N*-substituted glycosides; (a) (i) H_2_SO_4_, acetone; (ii) Na_2_CO_3_, H_2_O (70%) (b) TsCl, DMAP, Et_3_N, CH_2_Cl_2_ (64%) (c) NaN_3_, DMF (99%) (d) DMP, CH_2_Cl_2_ (89%) (e) NaBH_4_, MeOH (88%) (f) NaH, PMBCl, DMF (88%) (g) RaNi, H_2_, MeOH (93%) (h) (i) PMBNH_2_, CH_2_Cl_2_, 4 Å mol sieves (ii) NaBH_4_, MeOH (71%) (i) CAN, DCM/H_2_O (42%) (j) (i) *p*-anisidine, CH_2_Cl_2_, 4 Å mol sieves (ii) NaBH_4_, MeOH (82%) (k) TCCA, 1 M H_2_SO_4_, MeCN/H_2_O (75%) (l) Pd/C, H_2_, MeOH (92%).

**Scheme 2:**
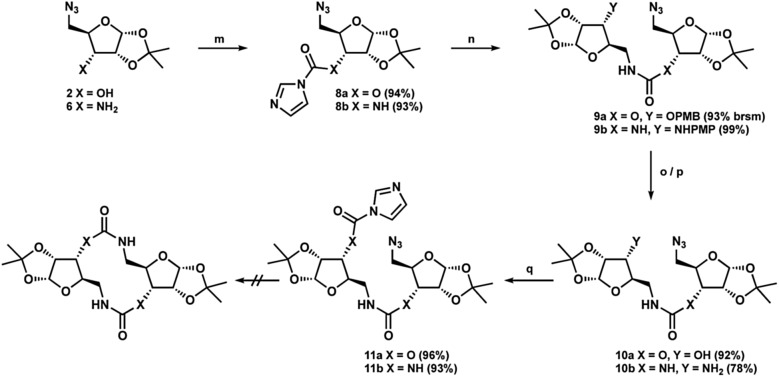
Coupling strategy to provide bis-glycosides: (m) CDI, THF (n) **4** or **7**, THF (o) DDQ, CH_2_Cl_2_/H_2_O (p) TCCA, 1 M H_2_SO_4_, MeCN/H_2_O (q) CDI, THF.

Next, we turned our attention to the macrocyclization reaction which would provide our desired abasic product. We hoped, as was the case with the initial coupling to afford the linear dimer, that upon revealing the amine, the compound would prefer intramolecular reaction. Knowing that this reaction would afford a fused pentacyclic scaffold with a central 12-membered macrocycle, we anticipated difficulties including oligomerization. Initial efforts to perform the macrocyclization with both **11a** and **11b** using several reduction methods including Lindlar's catalyst, RANEY® nickel, Pd/C, and PPh_3_ under various concentrations and temperatures yielded no appreciable product formation (Scheme 2). In both cases the azide was rapidly consumed as observed by TLC, however, oligomerization of the linear compounds was also observed and therefore the route was abandoned. Similar results were also disclosed by Jones *et al.* during their macrocyclization to afford the urea analog.^17^

Recently, synthetic chemists have adapted the bioorthogonal Staudinger ligation developed independently by Bertozzi and Raines to generate medium-sized macrolactams and peptides.^25–29^ This method, which has begun to see increased synthetic application, employs an activated thioester tethered to a phosphine to enforce an intramolecular Staudinger reaction with a distal azide. Subsequent reaction with the activated carbonyl moiety provides cyclic intermediate **14**, which following extrusion of the thiol and hydrolysis of the phosphine affords the desired macrolactam (Scheme 3). We postulated that this ligation strategy could be translated to our own macrocyclization efforts. To the best of our knowledge, this is the first report of this strategy being utilized to provide non-amide linked macrocycles.

**Scheme 3:**
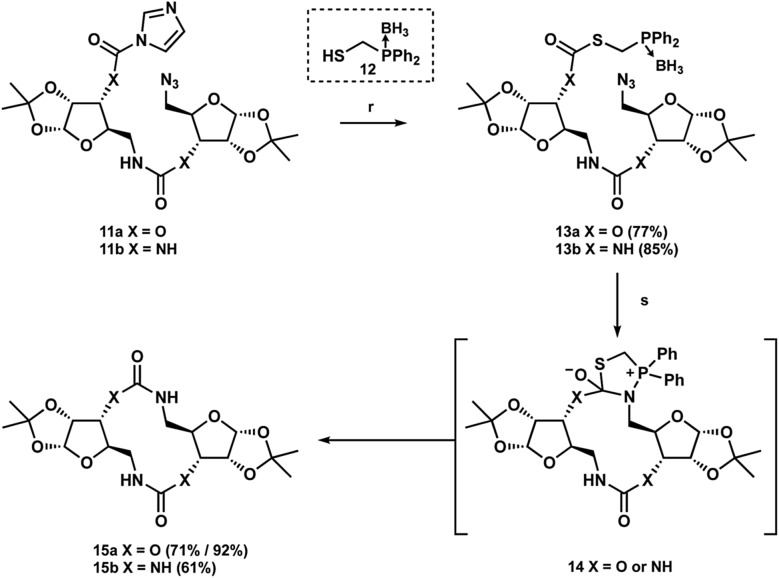
Staudinger ligation macrocyclization (r) **12**, DMAP, CH_2_Cl_2_ (s) DABCO, THF/H_2_O, reflux.

Using the typical borane-protected thiophosphine **12** initial investigations revealed nearly complete transformation into the desired thioester **13a** or thiourea **13b** after stirring for two days.^30–32^ With the addition of DMAP, we were able to decrease these reaction times to only two hours with little change in isolated yield (Scheme 2). With the thiocarbamate **13a** and thiourea **13b** in hand we were ready to begin screening the Staudinger conditions. Initial reaction of the thiocarbamate was performed under conventional heating by refluxing in THF/H_2_O with DABCO overnight at 80 °C, which gave the desired macrocycle product **15a** in 71% yield. The reaction to afford the urea macrocycle **15b** proceeded similarly to give the product in 61% yield. Although we were gratified at the prospect of being able to provide the respective macrocycles, we wondered if this reaction would be amenable to microwave conditions to decrease both reaction times as well as the temperature. While we could afford the carbamate macrocycle in 90% yield in only 5 minutes at 50 °C, we did not observe any product formation for the urea analog. We have not yet identified the cause of this result, but we propose that aggregation of the urea compound maybe be hindering its reactivity.

In conclusion, we have disclosed the first divergent route toward late-stage non-phosphate CDN precursors. This work provides a highly efficient and scalable route to orthogonally protected bis-substituted *N*,*O*- and *N*,*N*-glycosides in gram quantities, which we expect to find utility within the carbohydrate community. Our synthesis features an extended application of the Staudinger ligation to furnish carbamate- and urea-linked macrocycles. We believe that this method will also be valuable in providing non-amide or non-ester medium-sized macrocycles for the use in analog development. Ongoing studies in our laboratory are focused on expanding the repertoire of the Staudinger ligation as well as utilizing the *N*,*O*- and *N*,*N*-substituted glycosides to provide other non-phosphate linked scaffolds. We are also actively exploring the bis-glycosylation strategy from these abasic intermediates and will report our methods in due course.
