# Supplementary material for: A novel application of the Staudinger ligation to access neutral cyclic di-nucleotide analog precursors via a divergent method
Source: RSC Adv. 2017 Jun 7;7(47):29835–8. doi: 10.1039/c7ra06045a (PMC5472050; doi:10.1039/c7ra06045a)

Electronic Supplementary Material (ESI) for RSC Advances.
This journal is © The Royal Society of Chemistry 2017

A Novel Application of the Staudinger Ligation to Access Neutral Cyclic Di-Nucleotide Analog Precursors via a Divergent Method

Madison H. Fletcher; Claire E. Burns-Lynch; Kyle W. Knouse; Leslie T. Abraham; Charles W. DeBrosse; and William M. Wuest*

Department of Chemistry, 1901 N. 13th St., Temple University, Philadelphia, PA 19122

Supporting Information

General Notes ……………………………………………………………………………….S2
Experimental Procedures…………………………………………………………………….S3
References……………………………………………………………………………………S16
Spectral Data ………………………………………………………………………………..S17

General Notes.

NMR Spectra (^1H, ^{13}C) were obtained at 298K on a Bruker Avance 500 (500/125 MHz) or Bruker Avance 400 (400/100 MHz) Nuclear Magnetic Resonance spectrometer. ^1H-NMR/^{13}C-NMR spectra obtained in $CDCl_3$ and d6-DMSO were referenced to residual non-deuterated solvent. Spectra obtained in MeOD/D_2O were referenced to residual non-deuterated methanol. The following abbreviations are used to describe signal multiplicities: s (singlet), d (doublet), t (triplet), q (quartet), m (multiplet), br (broad), dd (doublet of doublets), dt (doublet of triplets). Accurate mass measurement data were acquired on a Waters LCT Premier XE by use of electrospray ionization with an internal lock mass reference of leucine enkephalin. The accurate mass electron ionization data were acquired by use of a Waters GCT Premier equipped with an Agilent GC oven for sample introduction. Internal EI lock mass accomplished with fragments of PFTBA. Waters instruments are calibrated, and report by use of neutral atom masses. Infrared spectra were obtained using a ThermoFisher FT-IR Nicolet iS5 (iD5 ATR) spectrophotometer. Thin Layer Chromatography was performed on Merck 60 F_{254} silica gel plates. Detection was performed using UV light, *p*-Anisaldehyde stain, or PMA stain. Flash chromatography was performed on a Biotage Isolera system using high performance silica gel or prepacked SNAP Ultra Columns. Microwave reactions were performed in a CEM Discover System, Model SP-1239 microwave.

Reactions, other than those requiring aqueous solvent, were performed using flame-dried glassware under argon atmosphere. Anhydrous dichloromethane (CH_2Cl_2), tetrahydrofuran (THF), dimethylformamide (DMF), and acetonitrile (MeCN) were obtained by passing the previously degassed solvents through activated alumina columns. Deuterated solvents and all reagents were purchased at the highest purity from commercially available sources used without further purification unless otherwise specified. Brine refers to a saturated aqueous solution of sodium chloride.

Experimental Procedures.

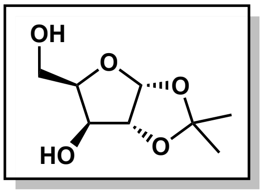

Acetonide-protected ribofuranose (S1). To a solution of H_2SO_4 (5 mL) in acetone (130 mL, 0.26 M) was added D-xylose (5 g, 33.3 mmol). This was stirred for 30 minutes until fully dissolved and the solution began to turn yellow. The reaction was cooled to 0°C and a solution of Na_2CO_3 (6.5 g, 61.3 mmol) in water (55 mL, 1.1M) was slowly added via dropping funnel (bubbles violently). Upon addition, the solution was became colorless and white solids immediately precipitated. After stirring for 3 hours at room temperature, additional solid Na_2CO_3 (6 g) was added and stirred for 30 minutes. Solids were removed via filtration and the remaining liquid was concentrated until only water remained (condensation in bump trap). The remaining aqueous layer was extracted with EtOAc (3 x 200 mL), organics dried over Na_2SO_4, filtered, and concentrated under reduced pressure to yield a clear syrup. The crude product was purified with flash chromatography (0→30%, 30→75% EtOAc/Hexanes) to afford diol **S1** as colorless crystalline solid (4.24 g, 70% yield). Characterization matches that in previously reported literature.[1] **Rf:** 0.06 (50% EtOAc/Hexanes), No UV activity, Phosphomolybdic acid stain: dark blue; **^{1}H NMR:** (500 MHz; $CDCl_3$): δ 1.32 (s, 3H), 1.48 (s, 3H), 2.69 (br, 1H), 4.05 (dd, J = 12.2, 2.2, 1H), 4.17-4.13 (m, 2H), 4.33 (d, J = 2.6, 1H), 4.52 (d, J = 3.7, 1H), 5.98 (d, J = 3.7, 1H).

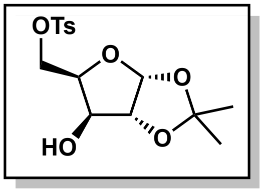

5'-Tosyl, 3'-(*S*) hydroxyl monomer (S2). To a CH_2Cl_2 solution (200 mL, 0.5 M) containing diol **S1** (19.36 g, 101.8 mmol) was added Et_3N (28 mL, 203.66 mmol, 2 equiv), DMAP (0.62 g, 5.09 mmol, 0.05 equiv), and TsCl (19.02 g, 99.8 mmol, 0.98 equiv) portionwise at 0°C. Warm to room temperature over 1 hour. Stir overnight or until starting material consumed by TLC. Dilute with water (~50 mL) and separate layers. Organics were washed once more with water (50 ml), saturated $NaHCO_3$ (3 x 50 mL), and 2N HCl (2 x 50 mL). The organic layer was dried over Na_2SO_4, filtered, and concentrated under reduced pressure. To the crude white solid was added a minimal amount cold ether, the solids filtered off and rinsed with additional cold ether to afford tosylate **S2** as a homogenous white solid (22 g, 64%). Characterization matches that in previously reported literature.[2] **Rf:** 0.42 (50% EtOAc/Hexanes), UV active, Phosphomolybdic acid stain: dark blue; **^{1}H NMR:** (500 MHz; $CDCl_3$): δ 1.30 (d, J = 0.4, 3H), 1.47 (s, 3H), 2.28 (dd, J = 5.1, 1.3, 1H), 2.46 (s, 3H), 4.15-4.11 (m, 1H), 4.35-4.30 (m, 3H), 4.52 (d, J = 3.6, 1H), 5.88 (d, J = 3.6, 1H), 7.36 (dd, J = 8.6, 0.7, 2H), 7.82-7.79 (m, 2H).

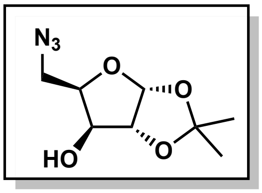

5’-Azide, 3’-(*S*)-hydroxyl monomer (S3). NaN_3 (8.05 g, 123.9 mmol, 5 equiv), was added to a solution of tosylate **S2** (8.53 g, 24.8 mmol) in DMF (250 mL, 0.1 M) and heated at 90°C overnight. Water (50 mL) was added after the reaction had cooled to room temperature and the solution was extracted with Et_2O (5 x 50 mL). Next the organic layer was washed with brine (5 x 20 mL), dried over $MgSO_4$, filtered, and concentrated under reduced pressure to afford the azide **S3** as a white solid after drying on high vacuum overnight (5.3 g, 99%). When necessary, the product was purified by flash chromatography (30% EtOAc/Hexanes). Characterization matches that in previously reported literature.[3] **Rf:** 0.53 (50% EtOAc/Hexanes), No UV activity, Phosphomolybdic acid stain: dark blue; **^{1}H NMR:** (400 MHz; $CDCl_3$) δ 1.31 (s, 3H), 1.49 (s, 3H), 2.10 (s, 1H), 3.67—3.57 (m, 2H), 4.30—4.24 (m, 2H), 4.52 (d, J = 3.7 Hz, 1H), 5.95 (d, J = 3.6 Hz, 1H).

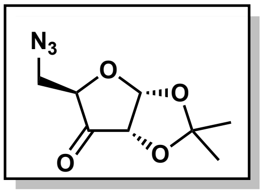

5’-Azide 3’-ketone monomer (1). To a solution of compound **S3** (3.07 g, 14.3 mmol) in CH_2Cl_2 (95 mL, 0.15 M) was added DMP (6.66 g, 15.7 mmol, 1.1 equiv). This was stirred for 4 hours. The mixture was quenched with a 1:1 solution of saturated $NaHCO_3$ and saturated $Na_2S_2O_3$ (40 mL) and was stirred for 20 minutes (top layer = milky white; bottom layer = clear). The aqueous layer was extracted with CH_2Cl_2 (3 x 75 mL), dried over Na_2SO_4, filtered, and concentrated under reduced pressure to afford ketone **1** as a clear oil with yellow suspension (2.71 g, 89%). Characterization matches that in previously reported literature.[4] **Rf:** 0.23 (30% EtOAc/Hexanes), UV active, Phosphomolybdic acid stain: dark blue; **^{1}H NMR:** (500 MHz; $CDCl_3$) δ 1.43 (s, 3H), 1.49 (s, 3H), 3.54 (dd, J = 13.2, 3.3, 1H), 3.68 (dd, J = 13.2, 3.2, 1H), 4.38 (dd, J = 4.4, 0.8, 1H), 4.50 (td, J = 3.2, 0.9, 1H), 6.15 (d, J = 4.4, 1H).

Carbamate macrocycle:

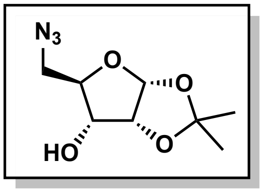

5’-Azide, 3’-(*R*)-hydroxyl monomer (2). To a cooled solution (0°C) solution of ketone **1** (4.33 g, 20.3 mmol) in anhydrous methanol (40 mL, 0.5 M) was added $NaBH_4$ (1.54 g, 40.6 mmol, 2 equiv) portionwise. This was stirred for 3 hours at room temperature. The reaction was brought back to 0°C, opened to air and slowly quenched by the addition of water until bubbling ceased. The solution was extracted into EtOAc (3 x 100 mL), organics dried over Na_2SO_4, filtered, and concentrated under reduced pressure to afford the (*R*)-alcohol **2** as a clear syrup (3.86 g, 88%) Characterization matches that in previously reported literature.[4] **Rf:** 0.67 (75% EtOAc/Hexanes) No UV activity, Phosphomolybdic acid stain: dark blue; **^{1}H NMR:** (500

MHz; $CDCl_3$): δ 1.38 (d, J = 0.6, 3H), 1.57 (d, J = 0.5, 4H), 2.34 (d, J = 10.6, 1H), 3.40 (dd, J = 13.4, 4.4, 1H), 3.70 (dd, J = 13.4, 2.5, 1H), 3.95-3.88 (m, 2H), 4.59 (t, J = 4.3, 1H), 5.85 (d, J = 3.9, 1H).

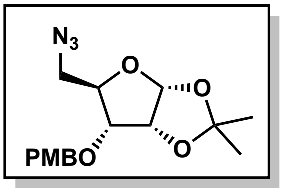

5'-Azide, 3'-O-PMB monomer (3). To a solution of (*R*)-alcohol **2** (1.02 g, 4.7 mmol) in DMF (24 mL, 0.2 M) cooled to 0°C was added NaH (60%, 0.283 g, 11.8 mmol, 2.5 equiv). The reaction turned dark red, and upon addition of *para*-methoxybenzyl chloride (0.96 mL, 7.1 mmol, 1.5 equiv) the reaction turned darker red. Stir at room temperature for 1 hour. The reaction was slowly quenched at 0°C with water (~10 mL). The solution was extracted into EtOAc (3 x 50 mL), washed with brine (5 x 50 mL), dried over Na_2SO_4, filtered, and concentrated under reduced pressure. The crude product was purified by flash chromatography (0→20% EtOAc/Hexanes) to afford PMB-protected alcohol **3** as a light yellow oil (1.39 g, 88%). **Rf:** 0.74 (50% EtOAc/Hexanes), UV active, Phosphomolybdic acid stain: dark red; **^{1}H NMR:** (400 MHz; $CDCl_3$) *δ* 1.36 (s, 3H), 1.58 (s, 3H), 3.21 (dd, *J* = 13.5, 3.9 Hz, 1H), 3.66—3.62 (m, 1H), 3.74 (dd, *J* = 8.9, 4.2 Hz, 1H), 3.81 (s, 3H), 4.17—4.14 (m, 1H), 4.49 (d, *J* = 11.6 Hz, 1H), 4.55 (t, *J* = 3.9 Hz, 1H), 4.69 (d, *J* = 11.6 Hz, 1H), 5.74 (d, *J* = 3.6 Hz, 1H), 6.90 (d, *J* = 8.4 Hz, 2H), 7.29 (d, *J* = 8.4 Hz, 2H); **^{13}C NMR:** (101 MHz; $CDCl_3$): δ 26.4, 26.8, 50.4, 55.2, 72.0, 77.2, 77.2, 77.3, 104.0, 113.1, 113.9, 129.3, 129.7, 159.5; **IR:** 1017, 1246, 1512, 2098, 2513, 2934, 2988 cm^{-1} ; **HRMS:** Accurate mass (ES+) Found 358.1389 (1.0 mDa), C16H21N3O5Na (M+Na^+) requires 358.1379

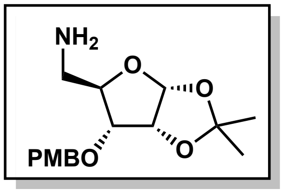

5'-Amine, 3'-O-PMB monomer (4). To a solution of the PMB-protected alcohol **3** (1.39 g, 4.14 mmol) in MeOH (21 mL, 0.2M) was added a slug of Raney Nickel. The reaction was purged with H_2 atmosphere and stirred for 1 hour. Raney Nickel was filtered off over a celite plug and rinsed twice with additional methanol. The remaining solution was concentrated under reduced pressure to afford amine **4** (1.19 g, 93%) as a light orange syrup. **Rf:** baseline (50% EtOAc/Hexanes), UV active, Phosphomolybdic acid stain: dark red ; **^{1}H NMR:** (400 MHz; $CDCl_3$): δ 1.35 (s, 3H), 1.58 (s, 3H), 2.75-2.71 (m, 1H), 3.06-3.01 (m, 1H), 3.65-3.61 (m, 1H), 3.80 (s, 3H), 4.07-4.02 (m, 1H), 4.48 (dd, J = 11.4, 0.4, 1H), 4.57-4.55 (m, 1H), 4.72-4.69 (m, 1H), 5.72-5.71 (m, 1H), 6.88 (d, J = 8.5, 2H), 7.29 (d, J = 8.3, 2H); **^{13}C NMR:** (126 MHz; $CDCl_3$): δ 26.5, 26.7, 50.3, 55.2, 71.7, 77.4, 77.9, 78.2, 104.0, 112.7, 113.7, 129.5, 129.7, 159.34; **IR:** 1019, 1246, 1513, 2027, 2160, 2548, 2935, 2981 cm^{-1}; **HRMS:** Accurate mass (ES+) Found 310.1656 (0.2 mDa), C16H24NO5 (M+H) requires 310.16540

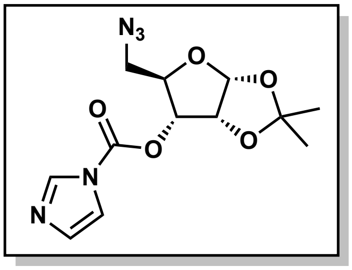

5’-Azide, 3’-*O*-carbonyl-imidazole monomer (8a). To a solution of (*R*)-alcohol **2** (2.49 g, 11.6 mmol) in THF (116 mL, 0.1 M) was added carbonyldiimidazole (4.80 g, 29.1 mmol, 2.5 equiv). The reaction was stirred for 1 hour, concentrated and immediately purified by flash chromatography to afford carbamate **8a** (3.37 g, 94%) as a white foam. **Rf:** 0.30 (50% EtOAc/Hexanes), UV active, Phosphomolybdic acid stain: light blue/green; **^{1}H NMR:** (500 MHz; $CDCl_3$): δ 1.36 (d, J = 0.5, 3H), 1.55 (s, 3H), 3.42 (dd, J = 13.7, 3.8, 1H), 3.76 (dd, J = 13.6, 3.2, 1H), 4.43-4.39 (m, 1H), 5.00-4.95 (m, 2H), 5.93 (d, J = 3.6, 1H), 7.10 (dd, J = 1.7, 0.8, 1H), 7.43 (t, J = 1.5, 1H), 8.15 (t, J = 1.0, 1H); **^{13}C NMR:** (126 MHz; $CDCl_3$): δ 26.5, 26.5, 50.2, 75.2, 76.3, 76.7, 77.0, 77.0, 77.3, 104.2, 113.7, 117.1, 130.9, 137.1, 147.6; **IR:** 1754, 2101, 2580, 2937, 2981, 3142 cm^{-1}; **HRMS:** Accurate mass (ES+) Found 310.1153 (0.2 mDa), C12H16N5O5 (M+H) requires 310.1151

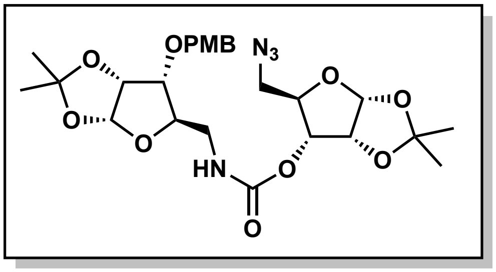

5a’-Azide, 3b’-O-PMB linear dimer (9a). To a solution of carbamate **8a** (1.19 g, 3.85 mmol) in THF (39 mL, 0.1M overall) was added amine **4** (1.19 g, 3.85 mmol, 1 equiv) in THF (2.1 mL, 0.1 M overall). The reaction was stirred overnight, concentrated and immediately purified by flash chromatography (50% EtOAc/Hexanes) to afford carbamate *bis*-glycoside **9a** (1.01 g, 93%, *brsm*) as a white foam. **Rf:** 0.46 (50% EtOAc/Hexanes), UV active, Phosphomolybdic acid stain: red to dark blue; **^{1}H NMR:** (500 MHz; $CDCl_3$) *δ* 1.34 (s, 3H), 1.35 (s, 3H), 1.56 (s, 3H), 1.58 (s, 3H), 3.33 (dd, *J* = 13.5, 4.6 Hz, 1H), 3.44—3.39 (m, 1H), 3.54—3.49 (m, 2H), 3.64 (dd, *J* = 13.6, 2.8 Hz, 1H), 3.81 (s, 3H), 4.10 (ddd, *J* = 8.9, 5.3, 3.5 Hz, 1H), 4.20 (ddd, *J* = 8.1, 4.7, 3.2 Hz, 1H), 4.52—4.48 (m, 2H), 4.67 (d, *J* = 11.6 Hz, 1H), 4.81—4.78 (m, 2H), 5.09 (t, *J* = 5.8 Hz, 1H), 5.68 (d, *J* = 3.7 Hz, 1H), 5.83 (d, *J* = 3.6 Hz, 1H), 6.88 (d, *J* = 8.7 Hz, 2H), 7.31 (d, *J* = 8.6 Hz, 2H); **^{13}C NMR:** (126 MHz; $CDCl_3$): δ 26.4, 26.5, 26.7, 26.7, 41.5, 50.8, 55.3, 72.0, 72.9, 76.8, 77.0, 77.3, 77.9, 78.0, 103.9, 104.0, 113.1, 113.3, 113.9, 129.4, 129.7, 155.0, 159.5, some carbons overlap; **IR:** 1021, 1245, 1514, 1728, 2034, 2100, 2160, 2444, 2982, 3349 cm^{-1}; **HRMS:** Accurate mass (ES+) Found 573.2163 (-1.0 mDa), C25H34N4O10Na (M+Na^+) requires 573.2173

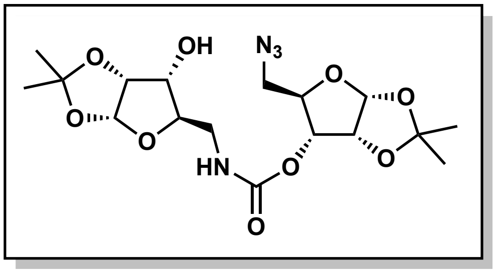

5a'-Azide, 3b'-(R/S) hydroxyl linear dimer (10a). To a solution of linear dimer **9a** (1.01g, 1.88 mmol) and DCM/H_2O (2:1, 19 mL, 0.1 M) stirring vigorously was added DDQ (0.855 g, 3.76 mmol, 2 equiv). The reaction immediately turned dark red and was stirred at room temperature for 3 hours. The biphasic solution was filtered over celite, and rinsed with water and DCM. The aqueous layer was extracted with DCM (3 x 100 mL, until DCM layer was clear), dried over Na_2SO_4, filtered, and concentrated under reduced pressure. The red residue was purified by flash chromatography (MeOH/EtOAc) to afford azide-alcohol *bis*-glycoside **10a** (0.742 g, 92%) as a white foam. **Rf:** 0.52 (5% MeOH/Et_2O), not UV active, Anisaldehyde stain: pink/brown; **^{1}H NMR:** (500 MHz; $CDCl_3$) δ 1.38 (s, 3H), 1.39 (s, 3H), 1.59 (s, 3H), 1.59 (s, 3H), 2.52 (d, J = 10.1 Hz, 1H), 3.36 (dd, J = 13.6, 4.5 Hz, 1H), 3.46 (dt, J = 14.3, 6.0 Hz, 1H), 3.70—3.61 (m, 2H), 3.77 (td, J = 9.5, 5.0 Hz, 1H), 3.88 (ddd, J = 8.9, 5.5, 3.5 Hz, 1H), 4.25—4.22 (m, 1H), 4.60 (t, J = 4.4 Hz, 1H), 4.85—4.82 (m, 2H), 5.22—5.20 (m, 1H), 5.80 (d, J = 3.8 Hz, 1H), 5.86 (d, J = 3.2 Hz, 1H); **^{13}C NMR:** (126 MHz; $CDCl_3$): δ 26.4, 26.4, 26.5, 26.7, 30.9, 41.6, 50.7, 72.7, 73.0, 76.8, 77.0, 77.3, 77.9, 78.5, 78.7, 103.8, 104.0, 112.9, 113.4, 155.4; **IR:** 871, 1001, 1214, 1239, 1374, 1539, 1723, 2032, 2101, 2160, 2981, 3354 cm^{-1}; **HRMS:** Accurate mass (ES+) Found 453.1589 (-0.8 mDa), C17H26N4O9Na (M+Na^+) requires 453.1597

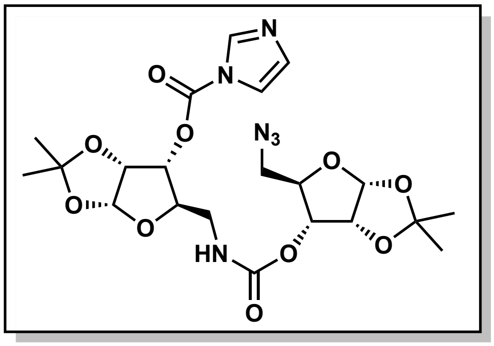

5a'-Azide, 3b'-O-carbonyl-imidazole linear dimer (11a). To a solution of compound **10a** (0.742 g, 1.72 mmol) in THF (17 mL, 0.1 M) was added carbonyldiimidazole (0.697 g, 4.31 mmol, 2.5 equiv). Stir at room temperature for 2 hours. The reaction was concentrated under reduced pressure and purified by flash chromatography (75% EtOAc/Hexanes to afford compound **11a** (0.870 g, 96%) as a white foam. **Rf:** 0.41 (75% EtOAc/Hexanes), light UV activity, Anisaldehyde: pink/brown; **^{1}H NMR:** (500 MHz; $CDCl_3$) δ 1.35 (s, 3H), 1.37 (s, 3H), 1.55 (s, 3H), 1 .57 (s, 3H), 3.33—3.29 (m, 1H), 3.53 (ddd, J = 14.7, 6.9, 4.8 Hz, 1H), 3.65—3.61 (m, 2H), 4.20 (ddd, J = 8.5, 4.5, 3.2 Hz, 1H), 4.35—4.32 (m, 1H), 4.80—4.72 (m, 3H), 4.92 (t, J = 4.4 Hz, 1H), 5.23—5.21 (m, 1H), 5.82 (d, J = 3.5 Hz, 1H), 5.87 (d, J = 3.8 Hz, 1H), 7.10 (s, 1H), 7.44 (s, 1H), 8.15 (s, 1H); **^{13}C NMR:** (126 MHz; $CDCl_3$): δ 26.4, 26.5, 26.5, 26.7, 41.0, 50.7, 73.1, 75.3, 76.0, 76.6, 76.9, 77.8, 103.9, 104.1, 113.3, 113.7, 131.0, 137.2, 147.8, 155.3; **IR:** 1018, 1724, 1766, 2029, 2101, 2160, 2523, 2987, 3337 cm^{-1}; **HRMS:** Accurate mass (ES+) Found 525.1964 (1.9 mDa), C21H29N6O10 (M+H) requires 525.1945

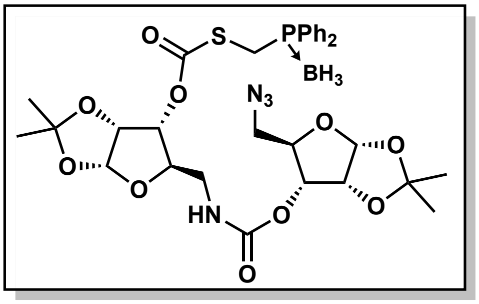

5a'-Azide, 3b'-O-carbonyl-thiophosphine linear dimer (13a). To a solution of compound **11a** (0.870 g, 1.66 mmol) in CH_2Cl_2 (10 of 11 mL, 0.15 M total) was added DMAP (2 mg, 0.017 mmol, 0.01 equiv) and thiol **12** (preparation described on S14 from references 5, 6, 7) (0.713 g, 2.9 mmol, 1.75 equiv) in the remaining 1 mL of CH_2Cl_2. The reaction was stirred overnight, concentrated and purified by flash chromatography to afford thiocarbamate **13a** (0.898 g, 77%) as a white foam. **Rf:** 0.85 (75% EtOAc/Hexanes), UV active, Anisaldehyde: yellow; **^{1}H NMR:** (500 MHz; $CDCl_3$): δ 1.00 (broad q, 3H), 1.32 (s, 3H), 1.35 (s, 3H), 1.49 (s, 3H), 1.56 (s, 3H), 3.33-3.23 (m, 2H), 3.56 (ddd, J = 14.5, 6.0, 3.3, 1H), 3.63 (dd, J = 13.5, 2.8, 1H), 3.75-3.66 (m, 2H), 4.11 (m 1H), 4.20 (m 1H), 4.54 (dd, J = 9.0, 4.9, 1H), 4.70 (t, J = 4.3, 1H), 4.80-4.76 (m, 2H), 5.16 (t, J = 5.9, 1H), 5.75 (d, J = 3.8, 1H), 5.82 (d, J = 3.4, 1H), 7.49-7.47 (m, 4H), 7.52 (m, 2H), 7.72-7.69 (m, 4H); **^{13}C NMR:** (126 MHz; $CDCl_3$): δ 26.1, 26.4, 26.4, 26.6, 26.7, 41.2, 50.7, 73.0, 75.6, 75.9, 76.6, 76.8, 77.0, 77.1, 77.3, 77.9, 103.9, 113.3, 113.5, 126.9, 127.3, 127.3, 128.9, 128.9, 131.8, 131.8, 131.8, 131.9, 132.5, 132.5, 155.1, 169.4, 169.4, splitting of aromatic carbons by phosphorus observed; **IR:** 999, 1107, 1149, 1722, 2030, 2100, 2160, 2390, 2982, 3355 cm^{-1}; **HRMS:** Accurate mass (ES+) Found 725.2170 (-2.4 mDa), C31H40BN4O10PSNa (M+Na$^+$) requires 725.2194

Conventional Staudinger Procedure: Into a 25 mL round bottom flask equipped with a reflux condenser was added thioester **5** (94 mg, 0.13 mmol) in THF (9.75 mL. 3:1 THF/H_2O, 0.01 M). Then, H_2O (3.25 mL) was added and was warmed to 80°C before the addition of DABCO (82 mg, 0.67 mmol, 5 equiv). This was refluxed at 80°C for 16-24 hours until the observed intermediate was fully converted to product by TLC. This solution was concentrated under reduced pressure, azeotropically dried with MeCN, and purified by flash chromatography (60% EtOAc/Hexanes) to afford macrocycle **7** (40 mg, 71 %) as a white foam.

Microwave Staudinger Procedure: Into a 5mL CEM microwave vial containing DABCO (5 equiv), and H_2O was added a solution of thioester **5** (1 equiv) dissolved in THF (9:1 THF/H_2O, 0.1 M). The vial was capped and heated in the microwave at 50°C for 5 minutes. This procedure was done twice, all solutions were combined and concentrated under reduced pressure, and purified by flash chromatography (60% EtOAc/Hexanes) to afford the macrocycle **7** as a white foam (theoretical yield: 125 mg, actual yield: 113 mg, 90%).

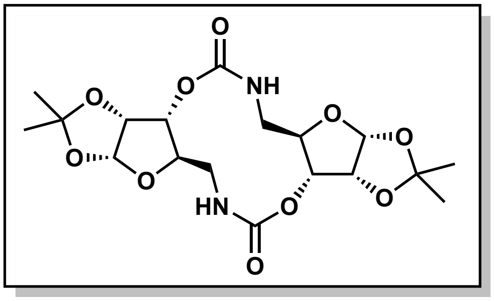

Carbamate macrocycle (15a). Rf: *Intermediate Rf:* 0.56 (75% EtOAc/Hexanes), UV active, Anisaldehyde stain: brown; *Product Rf*: 0.43 (75% EtOAc/Hexanes), No UV activity, Anisaldehyde stain: pink/brown; **^{1}H NMR:** (500 MHz; $CDCl_3$) *δ* 1.32 (s, 3H), 1.36 (s, 3H), 1.55 (s, 2H), 1.59 (s, 3H), 3.33—3.30 (dt, 1H), 3.44—3.40 (d, 1H), 3.80—3.73 (m, 2H), 4.00—3.96 (dt, 1H), 4.11—4.09 (m, 1H), 4.72—4.67 (m, 2H), 4.82—4.79 (m, 1H), 4.92—4.91 (m, 2H), 5.15 (dd, *J* = 9.7, 4.2 Hz, 1H), 5.74 (d, *J* = 3.5 Hz, 1H), 5.76 (d, *J* = 3.6 Hz, 1H); **^{13}C NMR:** (126 MHz; $CDCl_3$): δ 21.1, 21.2, 26.1, 26.3, 26.4, 39.5, 42.8, 71.5, 75.1, 75.6, 77.9, 78.2, 103.5, 103.6, 113.1, 113.5, 154.4, 157.1; **IR:** 1016, 1724, 2031, 2160, 2981, 3339 cm^{-1}; **HRMS:** Accurate mass (ES+) Found 431.1656 (-1.0 mDa), C18H27N2O10 (M+H) requires 431.1666

Urea Macrocycle:

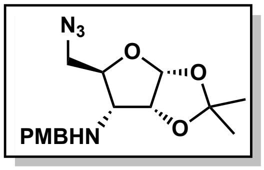

5'-Azide, 3'-(*R*)-NH-PMB monomer (5a) Ketone **1** (0.689 g, 3.22 mmol) in CH_2Cl_2 (30 mL, 0.1 M) was added to flame activated 4Å powdered molecular sieves (0.75 g). *p*-Methoxylbenzylamine (0.84 mL, 6.45 mmol, 2 equiv) was added and the suspension was stirred at 35°C for 6 hours. The molecular sieves were removed by filtration, and concentrated under reduced pressure to yield a dark brown residue. This crude imine was dissolved in anhydrous methanol (10 mL, 0.3 M) cooled to 0°C and $NaBH_4$ (3.42 g, 90.4 mmol, 2 equiv) was added portionwise and stirred overnight at ambient temperature. When necessary, an additional 0.75 equiv of $NaBH_4$ was added. The reaction was quenched with water, extracted into Et_2O (4 x 50 mL) and organics washed with brine. This was dried over Na_2SO_4, filtered, and concentrated under reduced pressure to afford the crude product as a light yellow oil. This was purified by flash chromatography (0→30% Et_2O/Hexanes) and concentrated to yield the PMB-protected amine **5a** as a light yellow syrup (0.77 g, 71%) and is slightly light sensitive. **Rf:** 0.20 (40% Et_2O/Hexanes) UV active, Phosphomolybdic acid: dark red to dark blue with heating ; **^{1}H NMR:** (400 MHz; $CDCl_3$) δ 1.34 (s, 3H), 1.51 (s, 3H), 1.85 (s, 1H), 2.95 (dd, J = 9.7, 4.5 Hz, 1H), 3.31 (dd, J = 13.3, 4.6 Hz, 1H), 3.70—3.63 (m, 2H), 3.83—3.76 (m, 4H), 3.86 (d, J = 12.9 Hz, 1H), 4.54 (t, J = 4.2 Hz, 1H), 5.78 (d, J = 3.8 Hz, 1H), 6.86 (d, J = 8.7 Hz, 2H), 7.27—7.25 (m, 2H); **^{13}C NMR:** (101 MHz; $CDCl_3$): δ 26.4, 26.6, 51.1, 51.4, 55.2, 61.1, 77.0, 79.0, 104.5, 112.0, 113.7, 129.1, 131.9, 158.7; **IR:** 1028, 1244, 1511, 2097, 2836, 2933, 2988, 3329 cm^{-1}; **HRMS:** Accurate mass (ES+) Found 335.1712 (-0.7 mDa), C16H23N4O4 (M+H) requires 335.1719

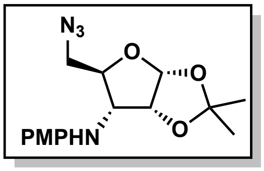

5’-Azide, 3’-(*R*)-NHPMP monomer (5b). Ketone **1** (9.64 g, 45.2 mmol) in CH_2Cl_2 (90 mL, 0.5 M) was added to flame activated 4Å powdered molecular sieves (10.6 g). *p*-Anisidine (6.69 g, 54.3 mmol, 1.2 equiv) was added and the suspension was stirred at 35°C overnight. The molecular sieves were removed by filtration, and concentrated under reduced pressure to yield a dark brown residue. This crude imine was dissolved in anhydrous methanol (90 mL, 0.5 M) cooled to 0°C and $NaBH_4$ (3.42 g, 90.4 mmol, 2 equiv) was added portionwise. This was stirred overnight at ambient temperature. The reaction was quenched with water, extracted into EtOAc (4 x 100 mL) and organics washed with saturated $NaHCO_3$. This was dried over Na_2SO_4, filtered, and concentrated under reduced pressure to afford the crude product as a dark brown syrup. This was purified by flash chromatography (5→20% EtOAc/Hexanes) and concentrated to yield the PMP-protected amine **5b** as a light yellow syrup (11.93 g, 82%) and is slightly light sensitive. **Rf:** 0.56 (30% EtOAc/Hexanes) UV active, Phosphomolybdic acid: dark red to dark blue with heating; **^{1}H NMR:** (400 MHz; $CDCl_3$) δ 1.32 (s, 3H), 1.56 (s, 3H), 3.29 (dd, J = 13.5, 3.6 Hz, 1H), 3.73 (q, J = 4.7 Hz, 4H), 3.80 (s, 2H), 3.91 (s, 1H), 4.62 (s, 1H), 5.84 (d, J = 3.7 Hz, 1H), 6.68 (d, J = 8.8 Hz, 2H), 6.77 (d, J = 8.9 Hz, 2H). ; **^{13}C NMR:** (101 MHz; $CDCl_3$): δ 26.4, 26.7, 50.6, 55.7, 58.0, 78.2, 79.1, 99.7, 104.1, 112.5, 115.0, 115.5, 140.7, 153.0; **IR:** 1028, 1233, 1511, 2097, 2833, 2982, 3369 cm^{-1}; **HRMS:** Accurate mass (ES+) Found 321.1559 (-0.4 mDa), C15H21N4O4 (M+H) requires 321.1563

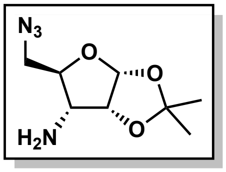

5’-Azide, 3’-(*R*)-NH_2 monomer (6). To a solution of PMP-protected amine **5b** (1.54 g, 4.81 mmol) in $MeCN/H_2O$ (24 mL, 1:1, 0.055 M) at 0°C was added TCCA (0.558 g, 2.40 mmol, 0.5 equiv) and 1M H_2SO_4 (4.81 mL). The reaction immediately turned dark red/purple was stirred at room temperature for two hours. The reaction was washed with CH_2Cl_2 (3 x 50 mL, orange layer), aqueous layer brought to a pH of 10 with a 10% NaOH solution (color change from purple to green then brown) and extracted with EtOAc (3 x 100 mL). The organics were dried over Na_2SO_4, filtered, and concentrated under reduced pressure to afford the free amine **6** as a light brown oil (0.773 g, 75%). **Rf:** 0.47 (10% $MeOH/CH_2Cl_2$), baseline (30% Et_2O/Hexanes), no UV activity, Ninhydrin: red/brown; **^{1}H NMR:** (500 MHz; $CDCl_3$) δ 1.36 (s, 3H), 1.39 (s, 2H), 1.54 (s, 3H), 3.16—3.13 (m, 1H), 3.38 (dd, J = 13.3, 4.4 Hz, 1H), 3.69 (dd, J = 13.3, 3.0 Hz, 1H), 3.81—3.77 (m, 1H), 4.47 (t, J = 4.3 Hz, 1H), 5.82 (d, J = 3.7 Hz, 1H). **^{13}C NMR:** (126 MHz; $CDCl_3$): δ 26.4, 26.6, 50.8, 55.8, 80.1, 80.5, 104.2, 112.1; **IR:** 1022, 2096, 2982, 3383 cm^{-1}; **HRMS:** Accurate mass (ES+) Found 215.1165 (2.1 mDa), C8H15N4O3 (M+H) requires 215.1144

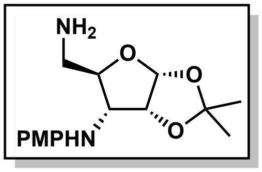

5'-Amine, 3'-NH-PMP monomer (7). To a solution of PMP-protected amine **5b** (1.21 g, 3.78 mmol) in MeOH (7.5 mL, 0.5M) was added 10% Pd/C (200 mg). The reaction was purged with H_2 atmosphere and stirred for overnight. Pd/C was filtered off over a celite plug and rinsed twice with additional methanol. The remaining solution was concentrated under reduced pressure to afford amine **7** (1.02 g, 92%) as a light red/orange syrup. **Rf:** baseline (50% EtOAc/Hexanes), UV active, Phosphomolybdic Acid: dark red; **^{1}H NMR:** (400 MHz; $CDCl_3$) δ 1.32 (s, 3H), 1.48—1.47 (m, 3H), 1.57 (s, 3H), 2.84—2.80 (m, 1H), 3.14—3.10 (m, 1H), 3.74 (s, 4H), 3.83—3.81 (m, 1H), 4.64 (t, J = 4.0 Hz, 1H), 5.82 (d, J = 3.8 Hz, 1H), 6.71 (d, J = 9.1 Hz, 2H), 6.77 (d, J = 9.1 Hz, 2H); **^{13}C NMR:** (126 MHz; $CDCl_3$): δ 26.4, 26.6, 42.4, 55.8, 58.4, 78.4, 81.0, 104.1, 112.2, 114.9, 115.5, 141.0, 152.8**; IR:** 821, 1005, 1233, 1511, 2031, 2160, 2934, 3366 cm^{-1}; **HRMS:** Accurate mass (ES+) Found 295.1685 (2.7 mDa), C15H23N2O4 (M+H) requires 295.1658

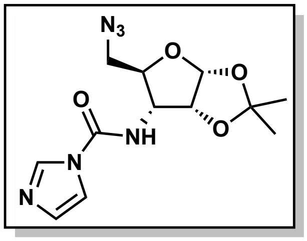

5'-Azide, 3'-N-carbonyl-imidazole monomer (8b). To a solution of amine **6** (0.299 g, 1.40 mmol) in THF (7 mL, 0.2 M) was added CDI (0.453 g, 2.79 mmol, 2 equiv). The reaction was stirred for 1 hour, concentrated and immediately purified by flash chromatography to afford urea monomer **8b** (0.398 g, 93%) as a white foam. **Rf:** 0.64 (10% MeOH/Et_2O), UV active, Anisaldehyde: pink/brown; **^{1}H NMR:** (500 MHz; $CDCl_3$) δ 1.39 (s, 2H), 1.60 (s, 3H), 3.49 (dd, J = 13.5, 4.7 Hz, 1H), 3.74 (dd, J = 13.5, 2.8 Hz, 1H), 4.06—4.02 (m, 1H), 4.41 (ddd, J = 9.2, 8.5, 5.2 Hz, 1H), 4.73 (dd, J = 5.2, 3.8 Hz, 1H), 5.96 (d, J = 3.8 Hz, 1H), 6.03—6.01 (m, 1H), 7.14 (dd, J = 1.6, 0.9 Hz, 1H), 7.32 (t, J = 1.5 Hz, 1H), 8.11 (s, 1H); **^{13}C NMR:** (126 MHz; $CDCl_3$): δ 26.4, 26.6, 51.2, 53.5, 78.6, 79.1, 104.3, 113.2, 115.7, 131.2, 136.0, 148.5**; IR:** 1525, 1711, 2102, 2527, 3419 cm^{-1}; **HRMS:** Accurate mass (ES+) Found 309.1307 (-0.4 mDa), C12H17N6O4 (M+H) requires 309.1311

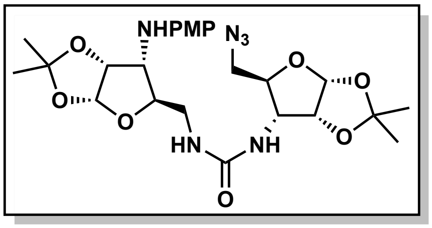

5a'-Azide, 3b'-N-PMP linear dimer (9b). To a solution of urea **8b** (0.398 g, 1.29 mmol) in THF (1.5 mL, 0.5 M overall) was added amine **7** (0.380 g, 1.29 mmol, 1 equiv) in THF (1 mL). The reaction was stirred for 4 hours, concentrated and immediately purified by flash chromatography (50% EtOAc/Hexanes) to afford urea linear dimer **9b** (0.323 g, 99%) as a white foam. **Rf:** 0.55 (10% MeOH/Et_2O), UV active, Anisaldehyde: brown; **^{1}H NMR:** (500 MHz; $CDCl_3$) δ 1.33 (s, 4H), 1.36 (s, 3H), 1.55 (s, 3H), 1.58 (s, 3H), 3.30 (dt, J = 14.4, 6.0 Hz, 1H), 3.36 (dd, J = 13.5, 5.6 Hz, 1H), 3.71—3.60 (m, 4H), 3.77 (s, 3H), 3.80 (s, 1H), 3.87 (ddd, J = 9.6, 5.6, 2.6 Hz, 1H), 3.91 (ddt, J = 8.5, 5.3, 2.7 Hz, 1H), 4.19 (td, J = 9.4, 5.0 Hz, 1H), 4.60 (dd, J =

4.8, 3.9 Hz, 1H), 4.66 (t, J = 4.1 Hz, 1H), 4.82—4.80 (m, 1H), 5.83 (d, J = 3.7 Hz, 1H), 5.86 (d, J = 3.7 Hz, 1H), 6.71—6.69 (m, 2H), 6.81—6.79 (m, 2H); **^{13}C NMR:** (126 MHz; $CDCl_3$): δ 26.4, 26.4, 26.7, 42.1, 51.4, 53.6, 55.7, 77.9, 79.0, 79.0, 79.2, 104.1, 104.2, 112.5, 115.0, 116.0, 140.5, 153.2, 157.6; **IR:** 1012, 1644, 2101, 2988, 3367 cm^{-1}; **HRMS:** Accurate mass (ES+) Found 535.2495 (-2.1 mDa), C24H35N6O8 (M+H) requires 535.2516

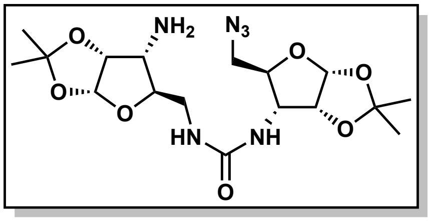

5a'-Azide, 3b'-NH$_2$ linear dimer (10b). To a solution of linear dimer **9b** (0.634 g, 1.19 mmol) and MeCN/H_2O (1:1, 22 mL, 0.055 M) at 0°C was added TCCA (0.137 g, 0.59 mmol, 0.5 equiv) and 1M H_2SO_4 (1.19 mL). The reaction was stirred at room temperature for one hour. The reaction was washed with CH_2Cl_2 (3 x 20 mL, yellow layer), aqueous layer brought to a pH of 10 with a 10% NaOH solution, and extracted with EtOAc (3 x 50 mL). The organics were dried over Na_2SO_4, filtered, and concentrated under reduced pressure to afford the amine **10b** as a white foam (0.408 g, 78%). **Rf:** 0.16 (10% MeOH/Et_2O), no UV activity, Anisaldehyde: pink; **^{1}H NMR:** (500 MHz; $CDCl_3$) δ 1.37 (s, 5H), 1.56 (d, J = 7.5 Hz, 8H), 2.97 (dd, J = 9.6, 4.7 Hz, 1H), 3.41 (dd, J = 13.5, 5.7 Hz, 1H), 3.52 (dd, J = 5.4, 3.3 Hz, 2H), 3.67 (dd, J = 13.5, 2.6 Hz, 1H), 3.73 (dt, J = 9.6, 4.0 Hz, 1H), 3.90 (ddd, J = 9.5, 5.7, 2.7 Hz, 1H), 4.22 (td, J = 9.3, 5.0 Hz, 1H), 4.51 (t, J = 4.2 Hz, 1H), 4.62 (dd, J = 4.9, 3.9 Hz, 1H), 4.92 (t, J = 5.9 Hz, 1H), 5.17 (s, 1H), 5.78 (d, J = 3.7 Hz, 1H), 5.88 (d, J = 3.8 Hz, 1H).; **^{13}C NMR:** (126 MHz; $CDCl_3$): δ 26.3, 26.4, 26.6, 26.7, 40.9, 51.5, 53.6, 56.2, 79.0, 79.2, 80.6, 80.7, 104.0, 104.2, 112.2, 112.5, 157.4; **IR:** 1007, 1553, 2099, 2982, 3356 cm^{-1}; **HRMS:** Accurate mass (ES+) Found 429.208 (-1.8 mDa), C17H29N6O7 (M+H) requires 429.2098

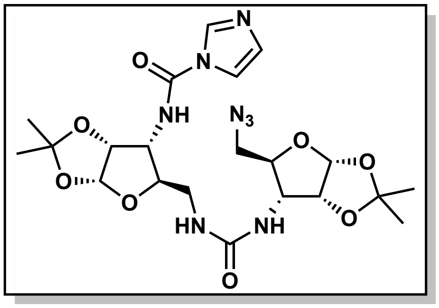

5a'-Azide, 3b'-N-carbonyl-imidazole linear dimer (11b). To a solution of compound **10b** (0.408 g, 0.92 mmol) in THF (9.2 mL, 0.1 M) was added CDI (0.449 g, 2.77 mmol, 3 equiv). Stir at room temperature for one hour. Concentrate reaction under reduced pressure and purify by flash chromatography (2% MeOH/Et_2O to afford the bis-urea linear dimer **11b** (0.446 g, 93%) as a white foam. **Rf:** 0.52 (10%MeOH/Et_2O), UV active, Anisaldehyde: brown; **^{1}H NMR:** (500 MHz; $CDCl_3$) δ 1.36 (s, 3H), 1.40 (s, 3H), 1.57 (s, 3H), 1.60 (s, 3H), 3.35 (ddt, J = 28.2, 11.4, 5.7 Hz, 2H), 3.65 (dd, J = 13.4, 2.7 Hz, 1H), 3.73 (ddd, J = 15.0, 6.8, 3.1 Hz, 1H), 3.90 (ddd, J = 9.5, 5.5, 2.8 Hz, 1H), 4.06 (ddd, J = 9.4, 5.8, 3.4 Hz, 1H), 4.21—4.16 (m, 2H), 4.56 (t, J = 4.4 Hz, 1H), 4.77 (dd, J = 4.8, 3.8 Hz, 1H), 5.14 (t, J = 6.3 Hz, 1H), 5.86 (d, J = 3.8 Hz, 1H), 5.91 (d, J = 3.7 Hz, 1H), 6.47—6.45 (m, 1H), 7.15 (dd, J = 1.6, 0.9 Hz,

1H), 7.39 (t, J = 1.5 Hz, 1H), 8.17 (t, J = 1.1 Hz, 1H). ; ^{13}**C NMR:** (126 MHz; $CDCl_3$): δ 26.3, 26.4, 26.5, 26.6, 31.0, 41.7, 51.4, 53.6, 78.6, 78.9, 79.0, 104.1, 104.2, 112.5, 113.2, 115.8, 131.0, 136.1, 148.9; **IR: 1011, 1556, 2100, 2934, 2989, 3188; HRMS:** Accurate mass (ES+) Found 523.2245 (-2.0 mDa), C21H31N8O8 (M+H) requires 523.2265

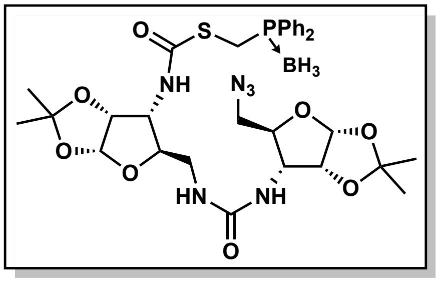

5a'-Azide, 3b'-N-carbonyl-thiophosphine linear dimer (13b). To a solution of compound **11b** (0.545 g, 1.66 mmol) in CH_2Cl_2 (9 mL, 0.1 M total) was added DMAP (1 mg, 0.01 mmol, 0.01 equiv) and thiol **12** (0.384 g, 1.56 mmol, 1.5 equiv) in CH_2Cl_2 (1 mL). The reaction was stirred overnight, concentrated and purified by flash chromatography (50% EtOAc/Hexanes) to afford thiourea **13b** (0.617 g, 85%) as a white foam. **Rf:** 0.21 (50% EtOAc/Hexanes), UV active, Anisaldehyde: yellow to brown; ^{1}**H NMR:** (500 MHz; $CDCl_3$) δ 0.89 (t, J = 6.9 Hz, 5H), 1.27 (s, 5H), 1.33 (s, 8H), 1.52 (s, 3H), 1.54 (s, 3H), 3.20—3.16 (m, 1H), 3.37 (dd, J = 13.6, 5.6 Hz, 1H), 3.64—3.60 (m, 2H), 3.73—3.69 (m, 1H), 3.79—3.76 (m, 1H), 3.88 (dd, J = 14.4, 6.1 Hz, 1H), 3.95—3.92 (m, 1H), 4.20—4.17 (m, 2H), 4.54 (t, J = 4.3 Hz, 1H), 4.62 (t, J = 4.2 Hz, 1H), 5.03—5.01 (m, 1H), 5.80 (d, J = 3.7 Hz, 1H), 5.86 (t, J = 7.3 Hz, 2H), 7.55—7.49 (m, 6H), 7.75—7.71 (m, 4H).; ^{13}**C NMR:** (126 MHz; $CDCl_3$): δ 26.3, 26.5, 26.5, 26.7, 29.7, 51.4, 53.8, 78.8, 78.8, 78.9, 79.1, 103.9, 104.3, 112.5, 113.1, 128.9, 128.9, 129.0 (2 carbons), 131.8, 131.8, 131.9, 132.5, (2 carbons) ; **IR:** 999, 2031, 2159, 2390, 2925, 3345 cm^{-1}; **HRMS:** Accurate mass (ES+) Found 701.2697 (0.3 mDa), C31H43BN6O8PS (M+H) requires 701.2694

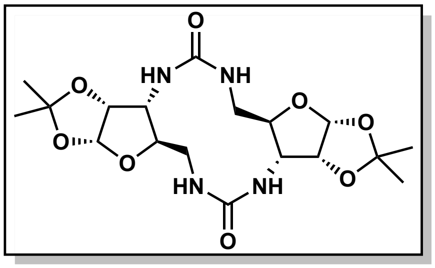

Urea macrocycle (15b). Thiourea **13b** (46 mg, 0.066 mmol) was treated according to the conventional Staudinger procedure on page S8 and purified using flash chromatography (column 1: 5% MeOH/Et_2O, column 2: 50% Acetone/Et_2O) to afford the urea macrocycle **15b** as a white foam (17 mg, 61%). **Rf:** 0.19 (5% MeOH/Et_2O), no UV activity, Anisaldehyde: pink/brown; ^{1}**H NMR:** *fluxional* (500 MHz; $CDCl_3$) δ 1.32 (s, 6H), 1.52 (s, 6H), 3.98 (broad s, 7H), 4.55 (broad t, 2H), 5.17 (broad s, 3H), 5.78 (d, J = 3.5 Hz, 2H) (some NH protons not observed); ^{13}**C NMR:** (126 MHz; $CDCl_3$): δ 26.3, 26.6, 41.9, 57.1, 79.6, 103.6, 112.6 (carbonyl carbon never observed)**; IR:** 1017, 1561, 1637, 2933, 2985, 3335 cm^{-1}**; HRMS:** Accurate mass (ES+) Found 429.197 (-1.5 mDa), C18H29N4O8 (M+H) requires 429.1985

Thiophosphine reagent:

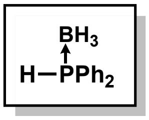

Phosphine-BH_3 (S4) To a flame dried round bottom was added chlorodiphenylphosphine (3 mL, 16.72 mmol) and Et_2O (60 mL, 0.28 M) which was cooled to 0°C. To this was added 1M BH_3•THF (18.40 mL, 18.40 mmol, 1.1 equiv) and LAH (0.70 g, 18.40 mmol, 1.1 equiv). Upon addition of LAH the reaction turned grey with a solid suspension and slight bubbling. The reaction was stirred at 0°C for 2 hours. The reaction was quenched at 0°C with 2N HCl *slowly* while open to air until bubbling ceased. The cloudy grey aqueous solution was extracted with Et_2O (3 x 100 mL). The organic layer was dried over $MgSO_4$, filtered, and concentrated under reduced pressure. The cloudy oil was purified by flash chromatography (10% Et_2O/Hexanes) to afford the borane protected phosphine **S4** as an opaque (white/clear) liquid (2.78 g, 83%). Characterization matches that in previously reported literature.[5] **Rf:** 0.46 (75% EtOAc/Hexanes), UV active, Phosphomolybdic acid stain: dark blue.

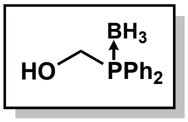

Phosphine-BH_3 alcohol (S5) To a solution of phosphine **S4** (2.78 g, 13.90 mmol) in THF (21 mL, 0.65 M) was added 30% formaldehyde in water (10.7 mL, 1.3 M with respect to phosphine) and KOH (0.761 g, 3.99 mmol, 3 equiv). The reaction was stirred for 3 hours at room temperature. The reaction was with water, and extracted with EtOAc (3 x 100 mL). The organic layer was dried over Na_2SO_4, filtered, and concentrated under reduced pressure. The resulting cloudy oil was azeotropically dried with toluene (3 x 10 mL) and filtered over silica gel to afford the alcohol **S5** as a cloudy thick oil (3.00 g, 94%). Characterization matches that in previously reported literature.[6] **Rf:** 0.58 (25% EtOAc/Hexanes), UV active, Phosphomolybdic acid stain: dark blue.

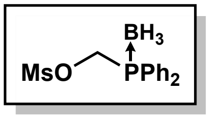

Phosphine-BH_3 mesylate (S6) To a solution of alcohol **S5** (3.0 g, 13.04 mmol) in DCM (26 mL, 0.5 M) was added Et_3N (3.62 mL, 26.08 mmol, 2 equiv). The reaction was cooled to 0°C and MsCl (1.5 mL, 19.30 mmol, 1.48 equiv) was added. Upon addition the reaction turned yellow and was stirred overnight at room temperature. The reaction was concentrated under

reduced pressure, taken up in EtOAc and filtered over celite. The filtrate was concentrated again, and immediately purified by flash chromatography (20% EtOAc/Hexanes) to afford the mesylate **S6** as a thick light yellow oil (2.0 g, 50%).[6] **Rf:** 0.24 (25% EtOAc/Hexanes), UV active, Phosphomolybdic acid stain: dark blue.

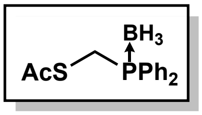

Phosphine-BH$_3$ thioacetate (S7) To a solution of mesylate **S6** (2.0 g, 6.49 mmol) in DMF (11.6 mL, 0.56 M) was added thioacetic acid (0.70 mL, 9.99 mmol, 1.54 equiv) and the reaction was cooled to -20°C. Upon the slow addition of $CsCO_3$ (4.14 g, 12.72 mmol, 1.96 equiv) the reaction turns orange. The reaction was allowed to warm to room temperature over 8 hours and subsequently stirred overnight. The light tan suspension was carefully diluted with water (30 mL) which began to bubble and turn clear. The aqueous was extracted with EtOAc (3 x 100 mL) and washed with brine (3 x 20 mL). The light yellow organic layer was dried over Na_2SO_4, filtered, and concentrated under reduced pressure to afford crude thioacetate **S7** as a clear oil (1.92 g). Characterization matches that in previously reported literature.[6]

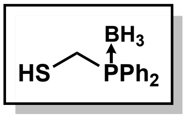

Phosphine-BH$_3$ thiol (12) Thioacetate **S7** (1.87 g, 6.49 mmol) was dissolved in MeOH (13 mL, 0.5 M) to which was added NaOH (0.52 g, 12.98 mmol, 2 equiv). The reaction was stirred for 1.5 hours and reduced. The crude syrup was diluted with water and extracted with EtOAc (3 x 100 mL). The organic layer was dried over Na_2SO_4, filtered, and concentrated under reduced pressure to afford the thiol **12** as a clear/opaque oil (1.46 g, 91% over two steps). Characterization matches that in previously reported literature.[7] **Rf:** 0.60 (30% Et_2O/Hexanes x 2), UV active, Phosphomolybdic acid stain: dark blue.

References:

(1) J. Moravcová, J. Čapková and J. Staněk, *Carbohydr. Res.*, **1994**, *263*, 61–66.

(2) G. Sharma and T. Gopinath, *Tetrahedron*, 2003, **59**, 6521–6530.

(3) J. Mcdevitt and P. Lansbury, *J. Am. Chem. Soc.*, 1996, **118**, 3818–3828.

(4) Ewing, D. F.; Goethals, G.; Mackenzie, G.; Martin, P.; Ronco, G.; Vanbaelinghem, L.; Villa, P. *Carbohydr. Res.* **1999**, *321* , 190–196.

(5) Wyatt, P.; Eley, H.; Charmant, J.; Daniel, B.J.; Kantacha, A. *Eur. J. Org. Chem.*, **2003**, *2003*, 4216-4226.

(6) He, Y.; Hinklin, R. J.; Chang, J.; Kiessling, L. L. *Org. Lett.* **2004**, *6* (24), 4479–4482.

(7) Pötzsch, R.; Fleischmann, S.; Tock, C.; Komber, H.; Voit, B. I. *Macromolecules* **2011**, *44* (9), 3260–3269.

Compound **S1** ^{1}H NMR ($CDCl_3$)

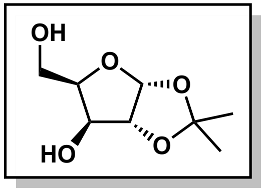

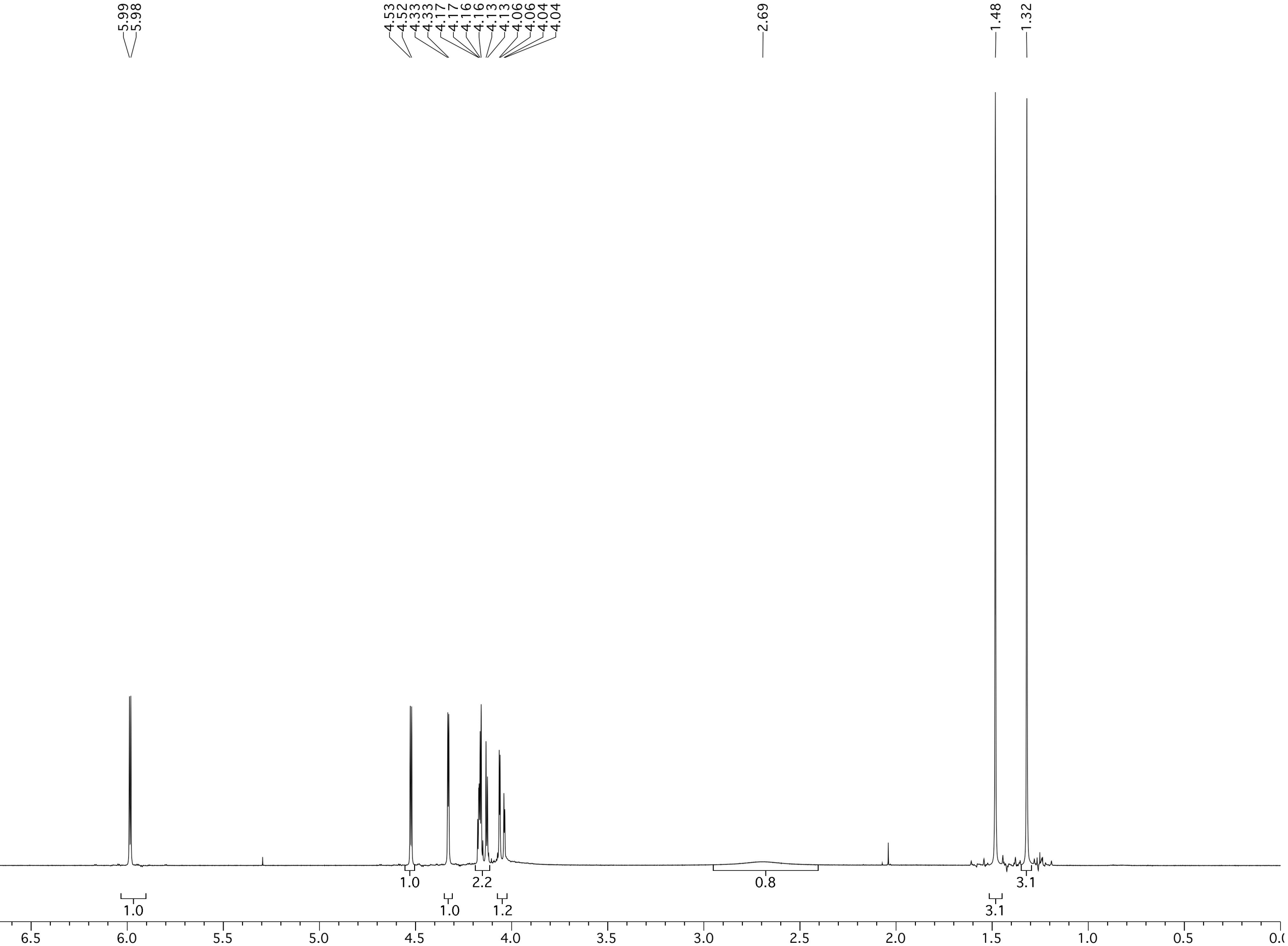

Compound **S2** ^{1}H NMR ($CDCl_3$)

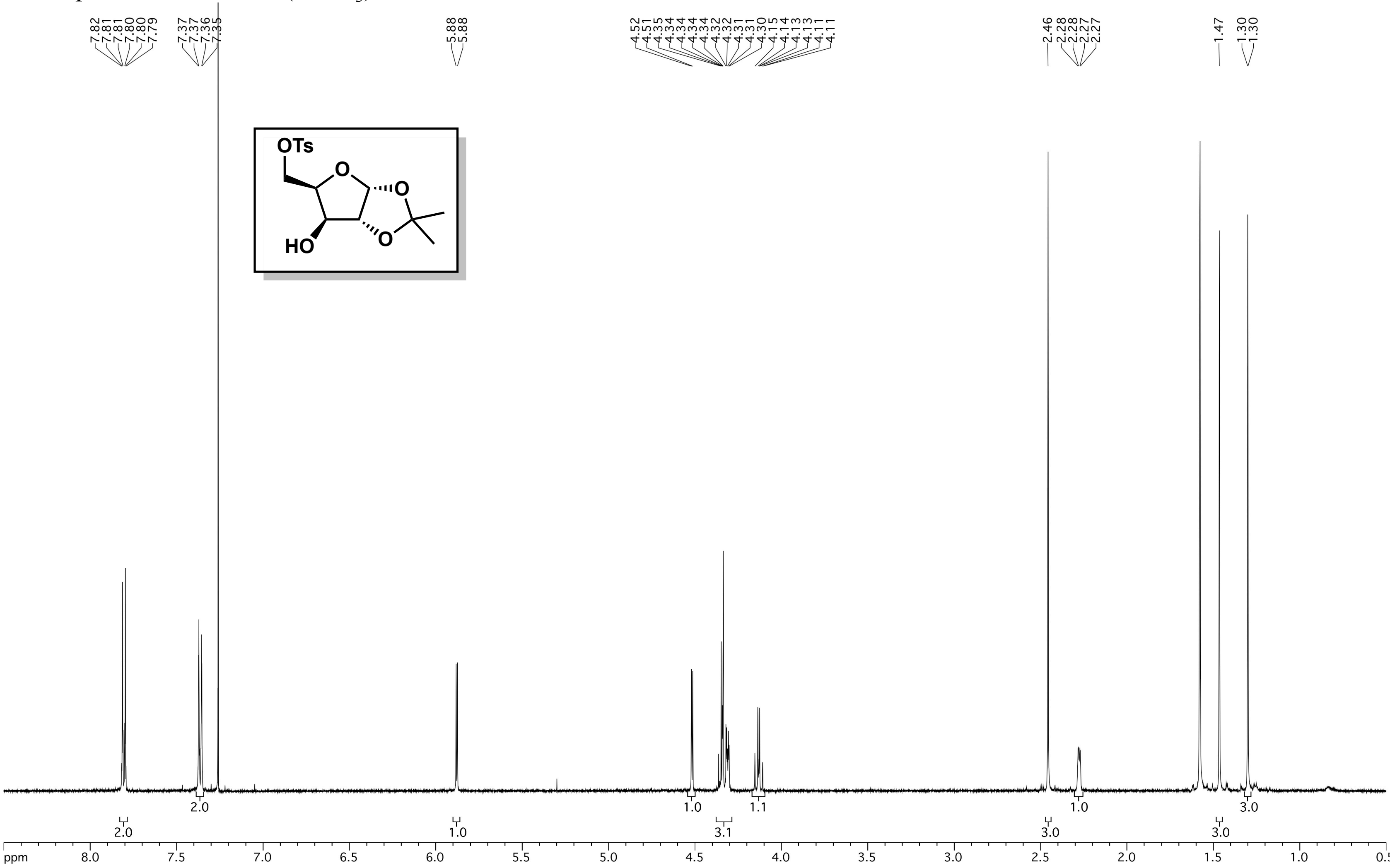

Compound **S3** ^{1}H NMR ($CDCl_3$)

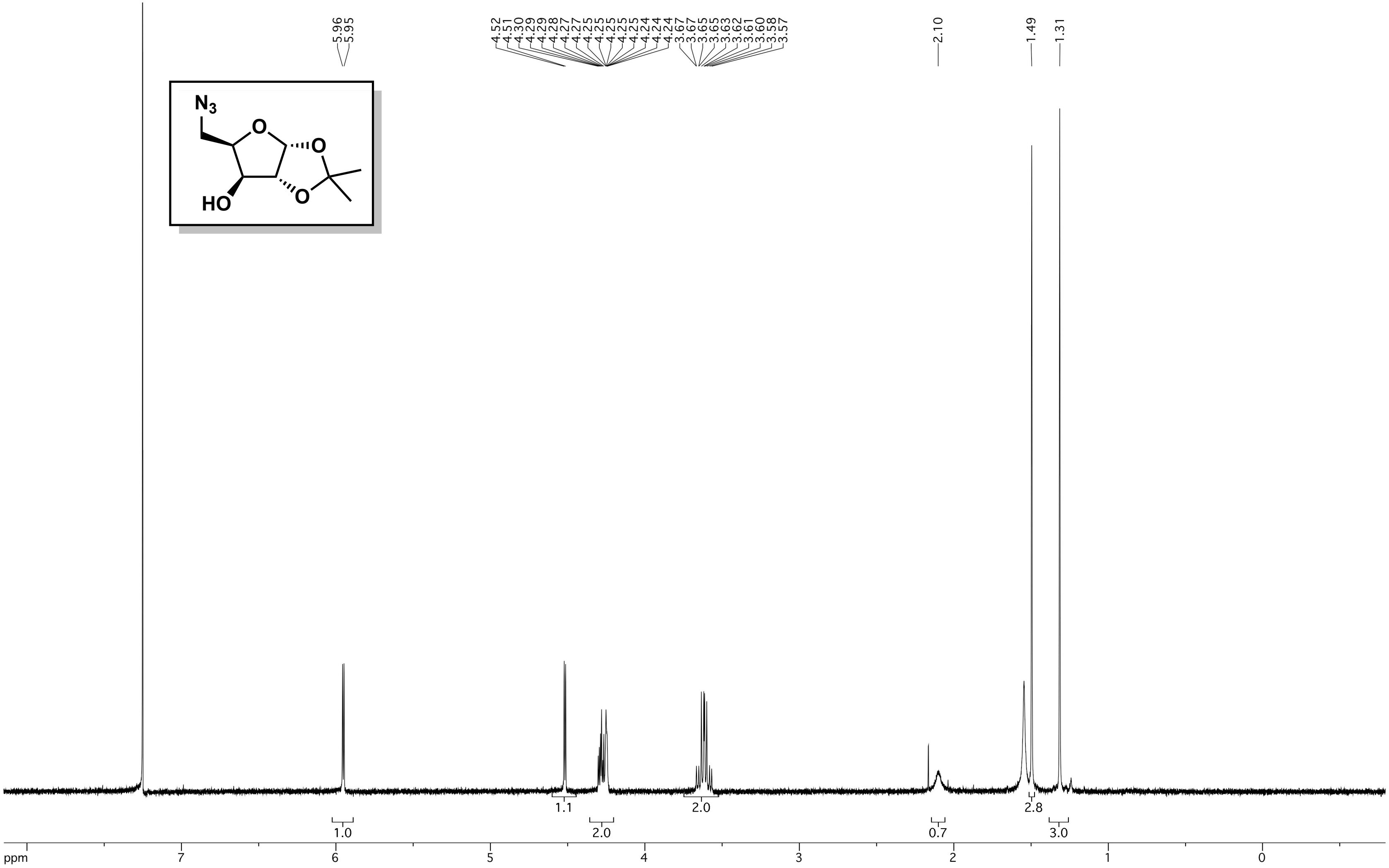

Compound **1** ^{1}H NMR ($CDCl_3$)

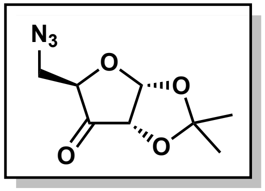

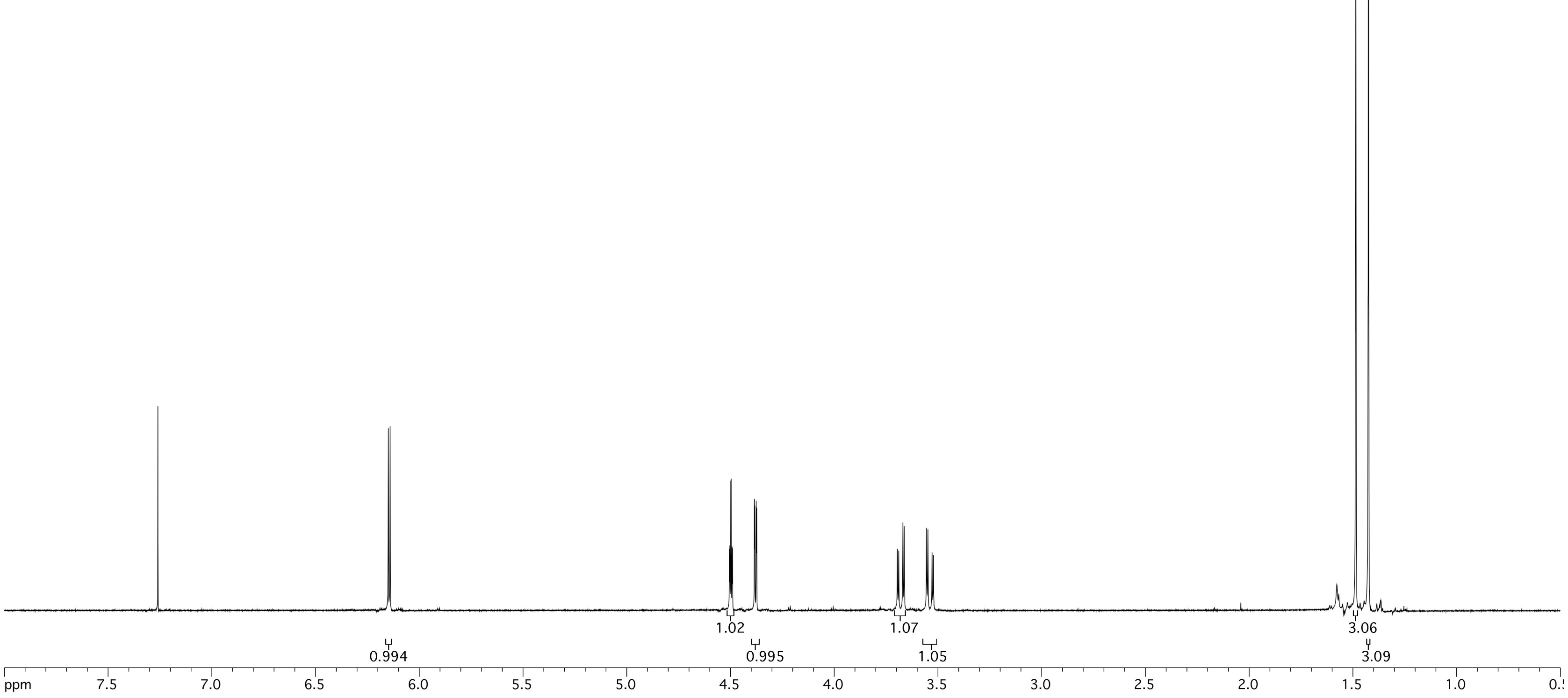

Compound **2** ^{1}H NMR ($CDCl_3$)

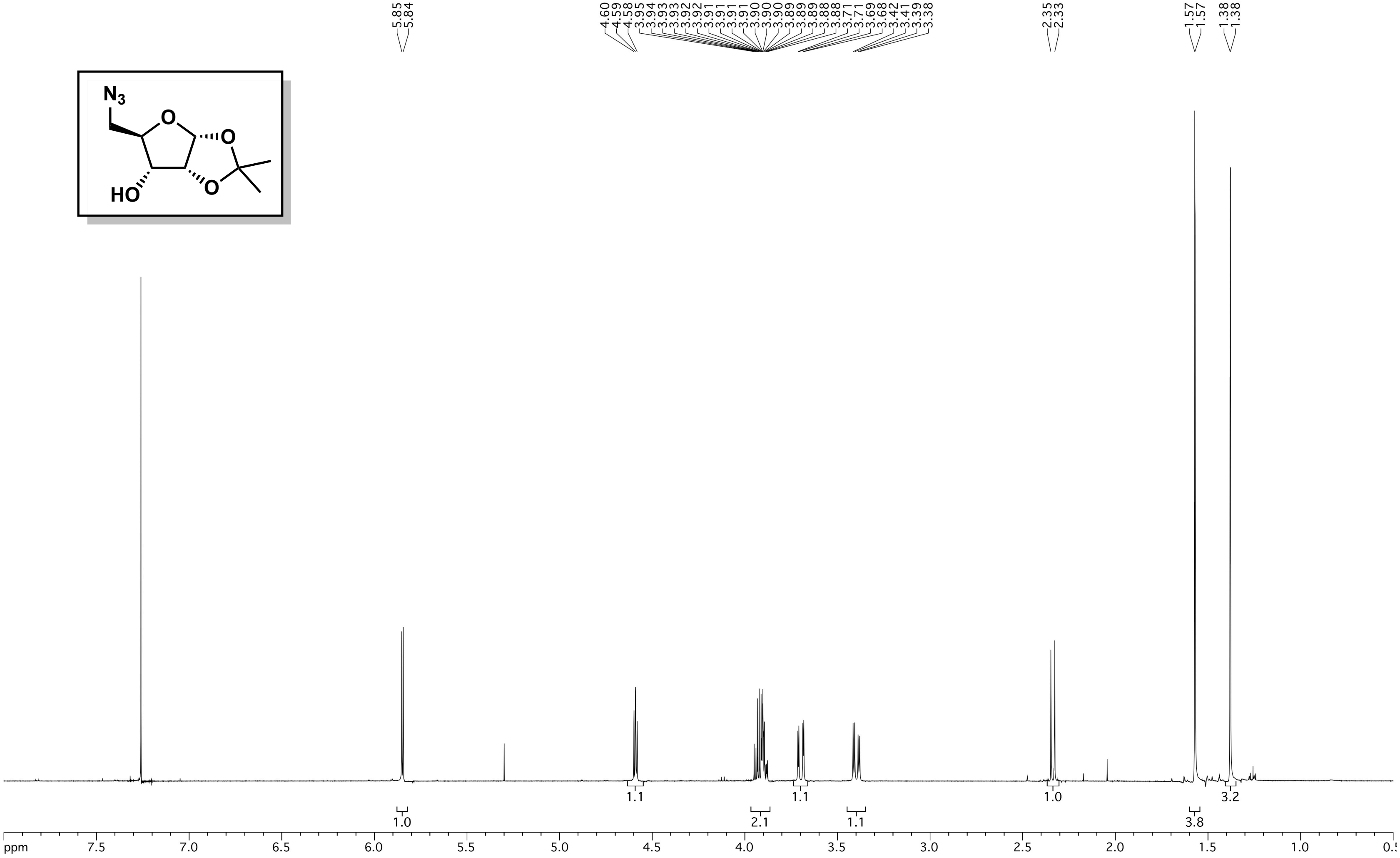

Compound **3** ^{1}H NMR($CDCl_3$)

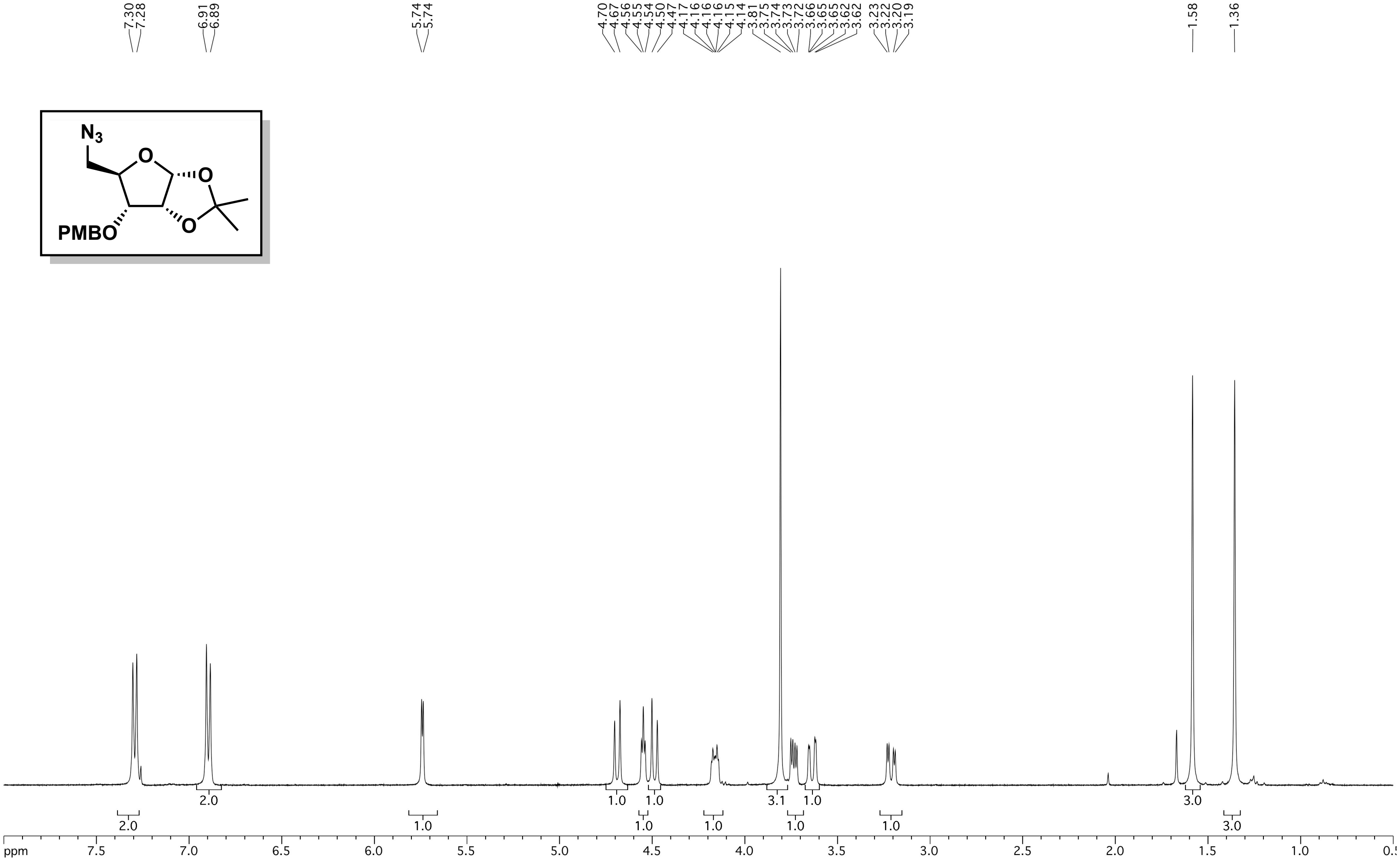

Compound **3** ^{13}C NMR ($CDCl_3$)

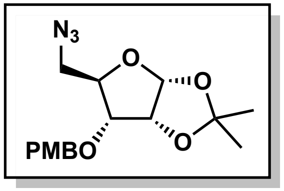

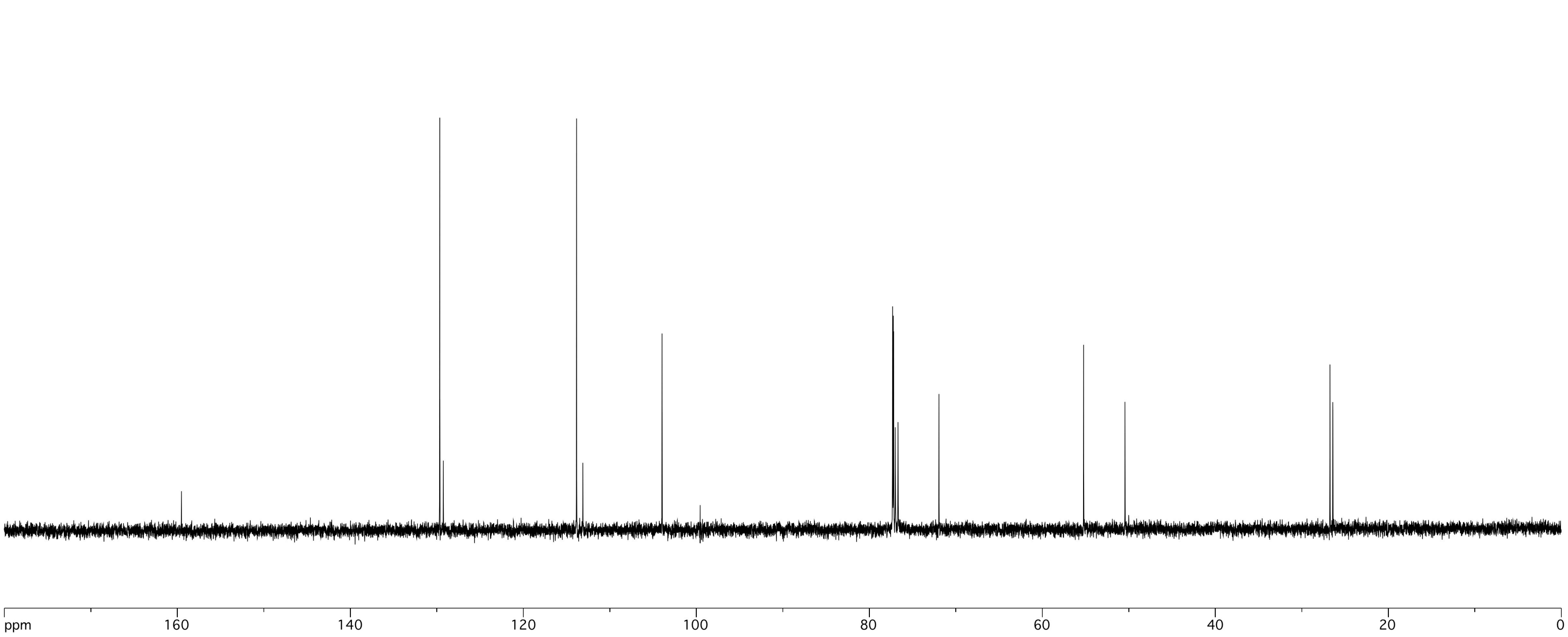

Compound **3** FTIR

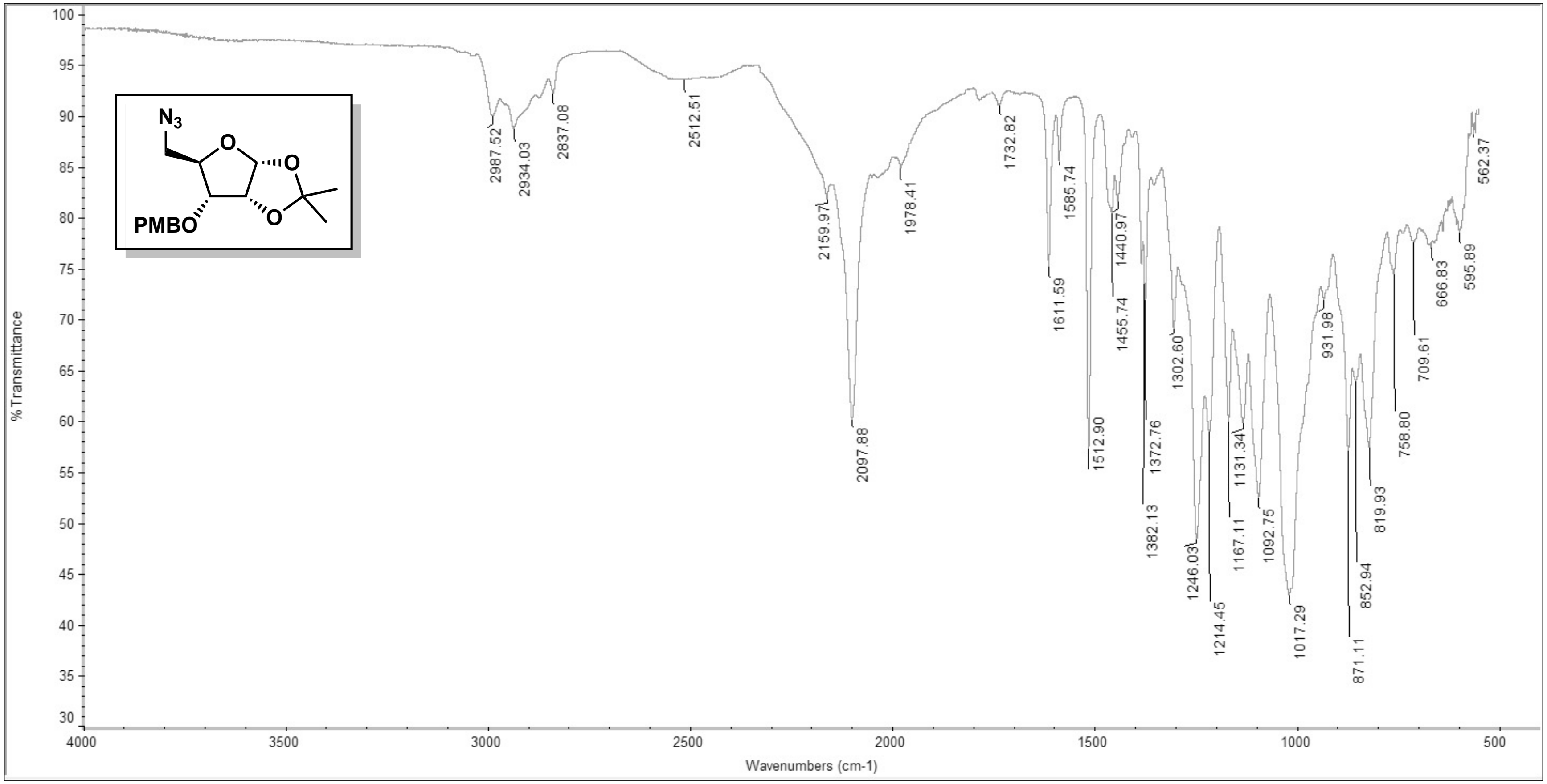

Compound **4** ^{1}H NMR ($CDCl_3$)

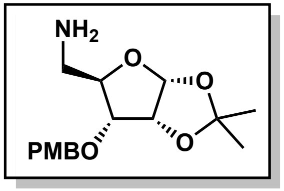

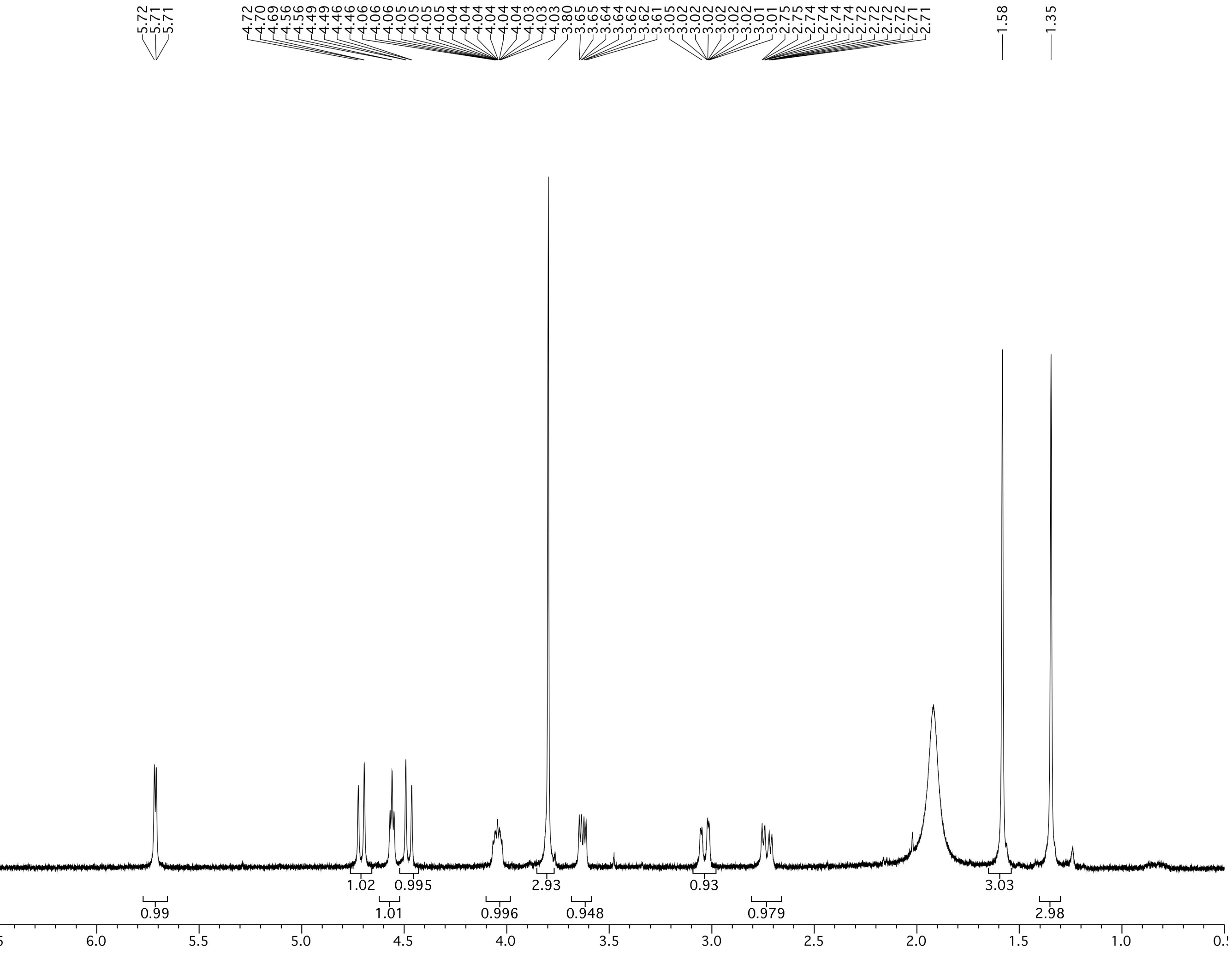

Compound **4** ^{13}C NMR ($CDCl_3$)

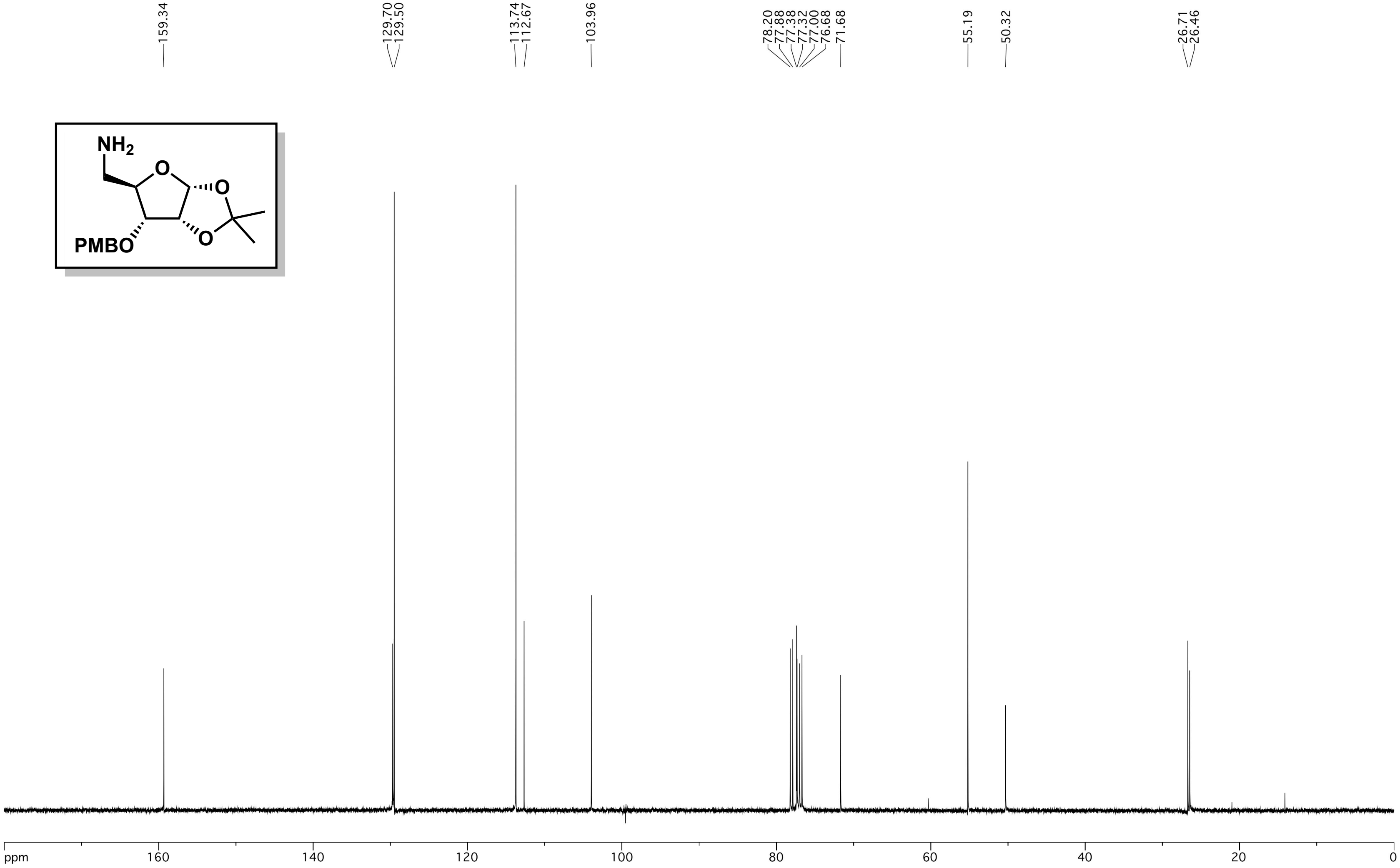

Compound **4** FTIR

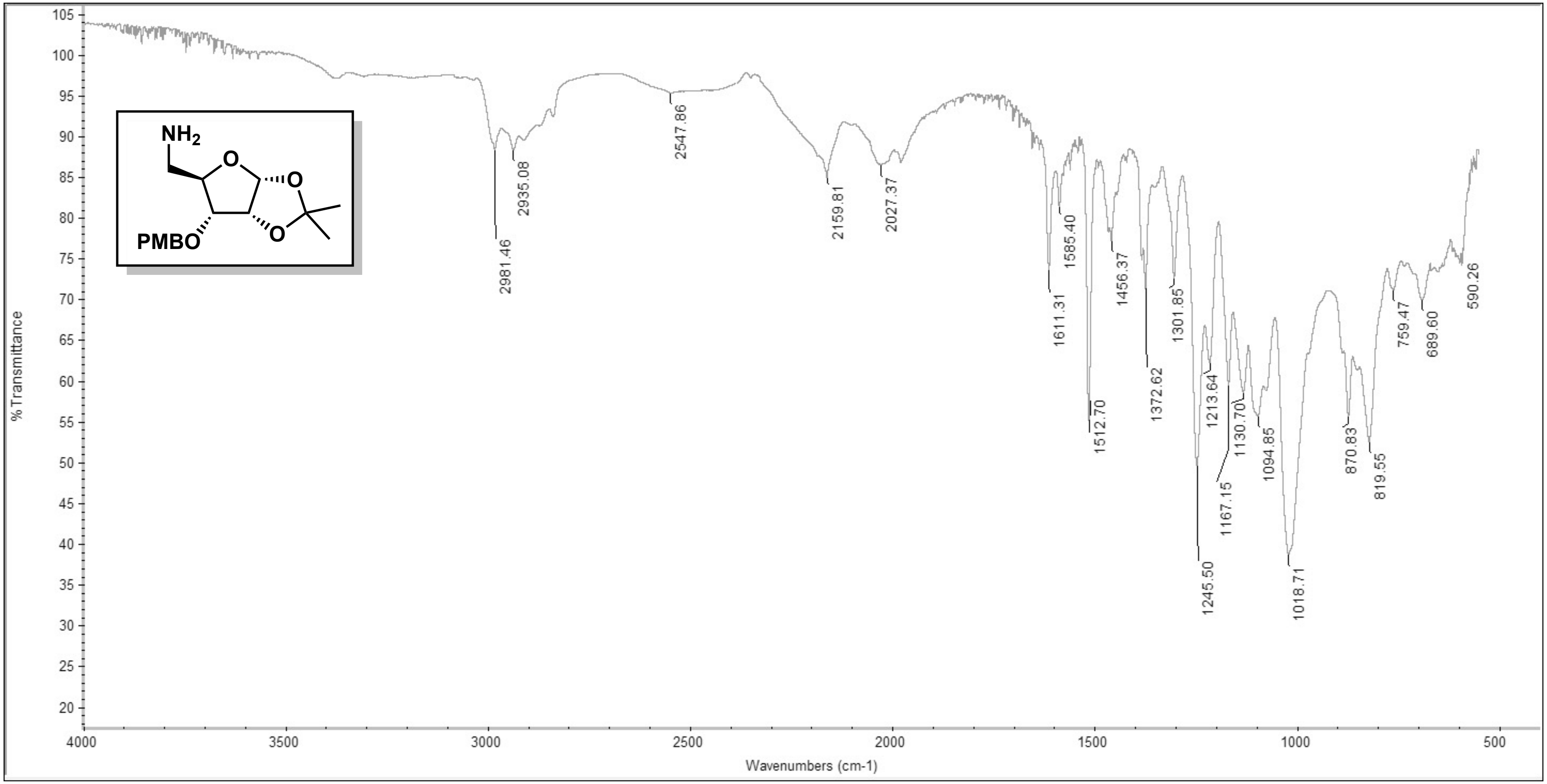

Compound **8a** ^{1}H NMR ($CDCl_3$)

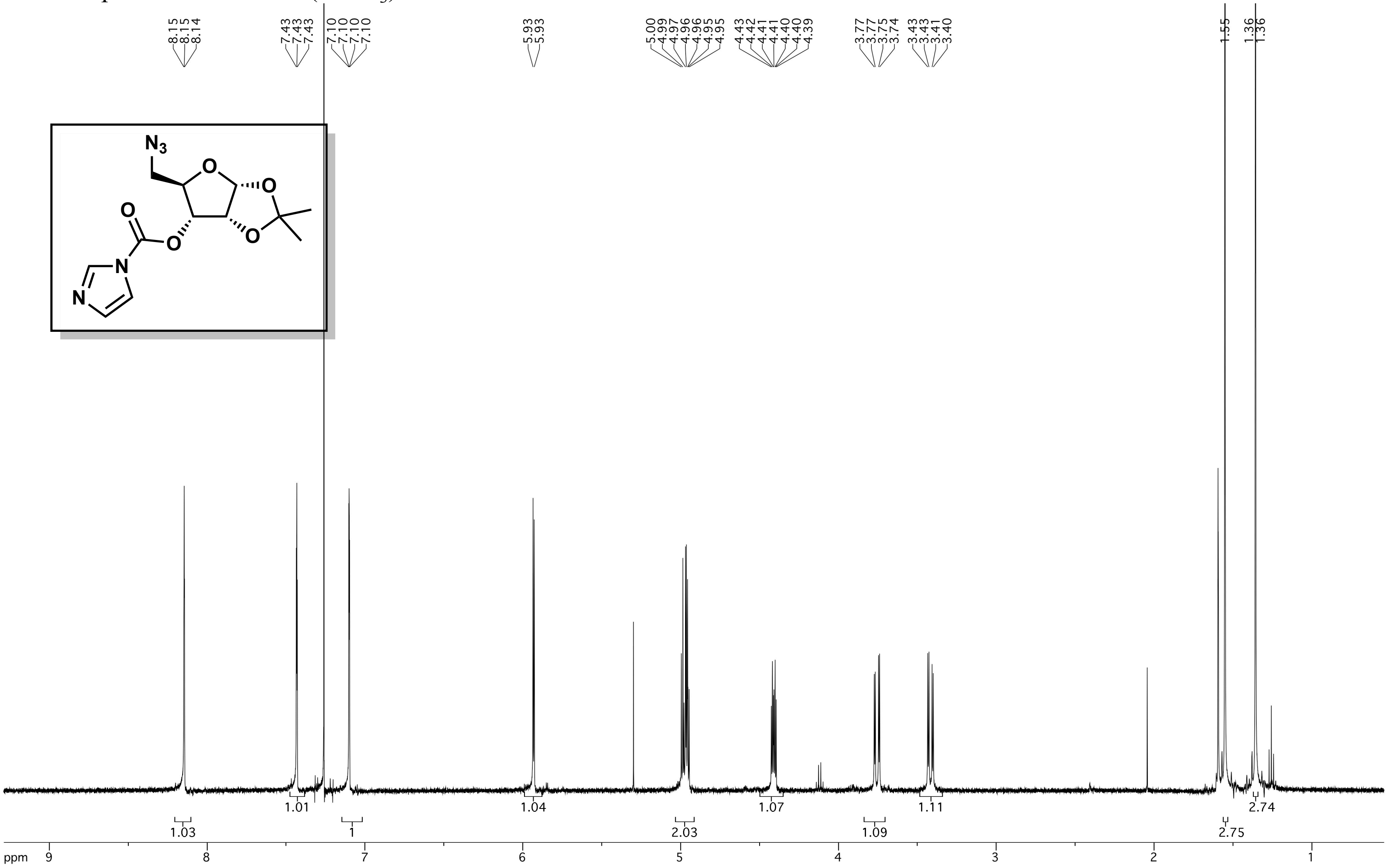

Compound **8a** ^{13}C NMR ($CDCl_3$)

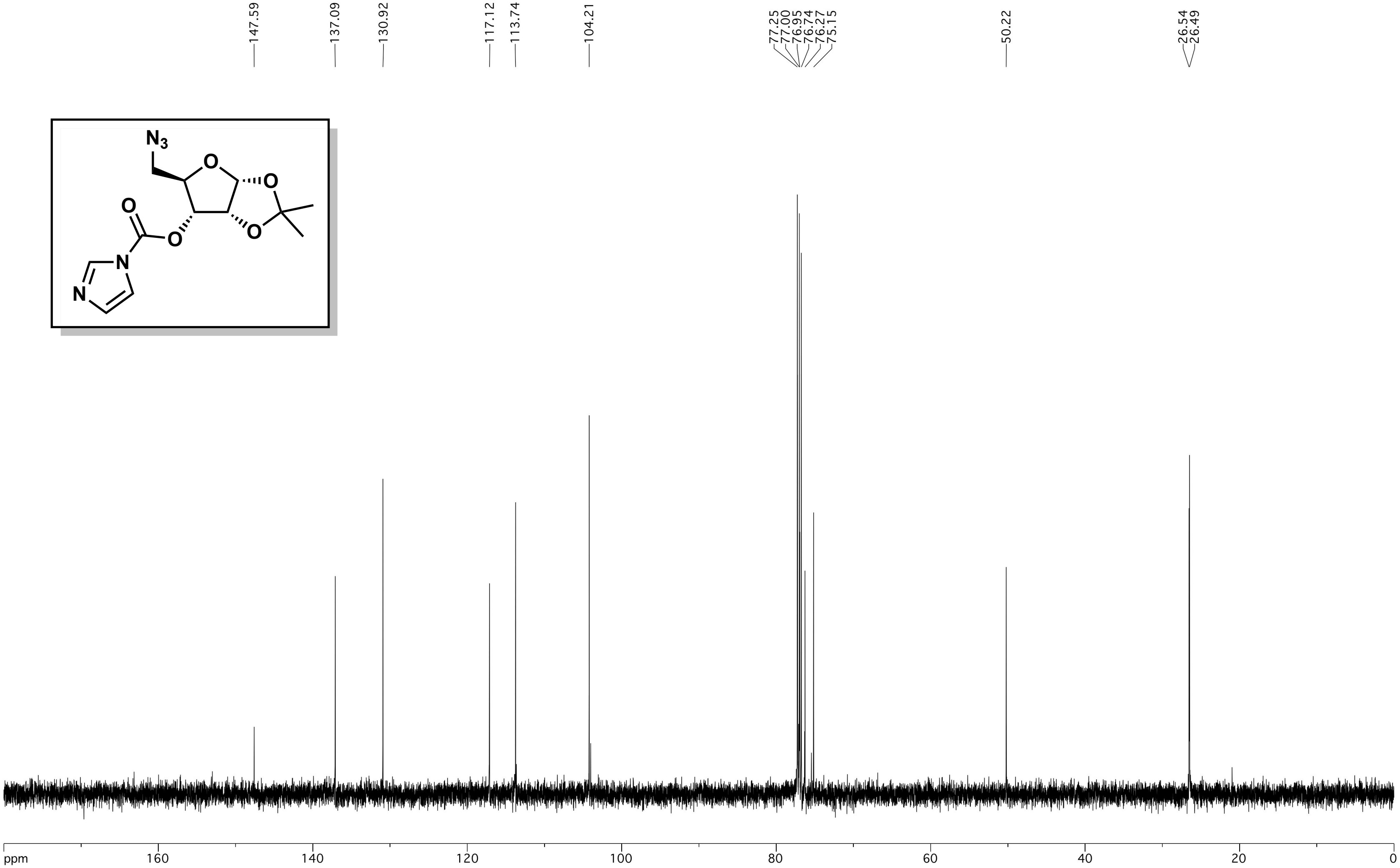

Compound **8a** FTIR

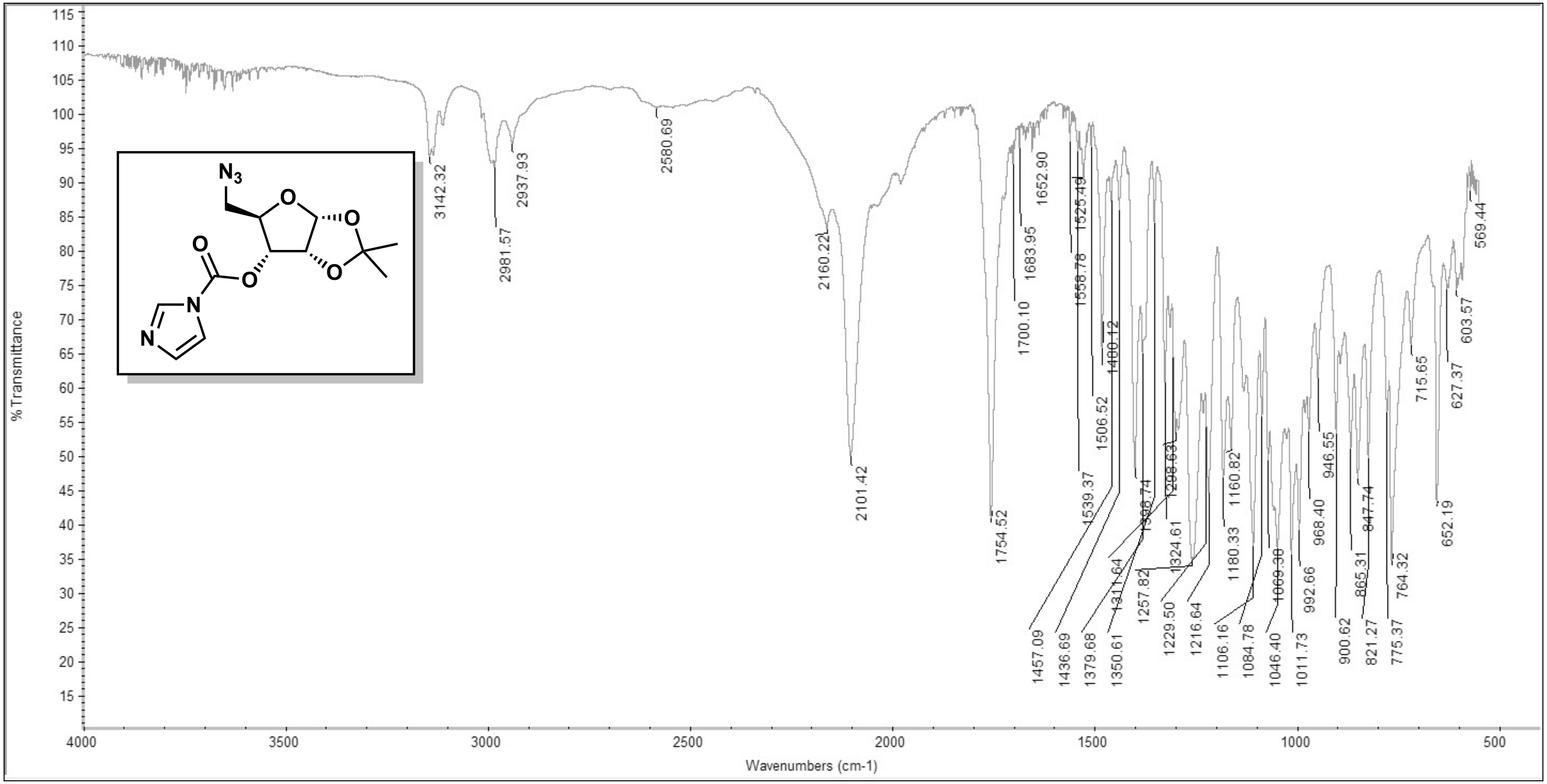

Compound **9a** ^{1}H NMR ($CDCl_3$)

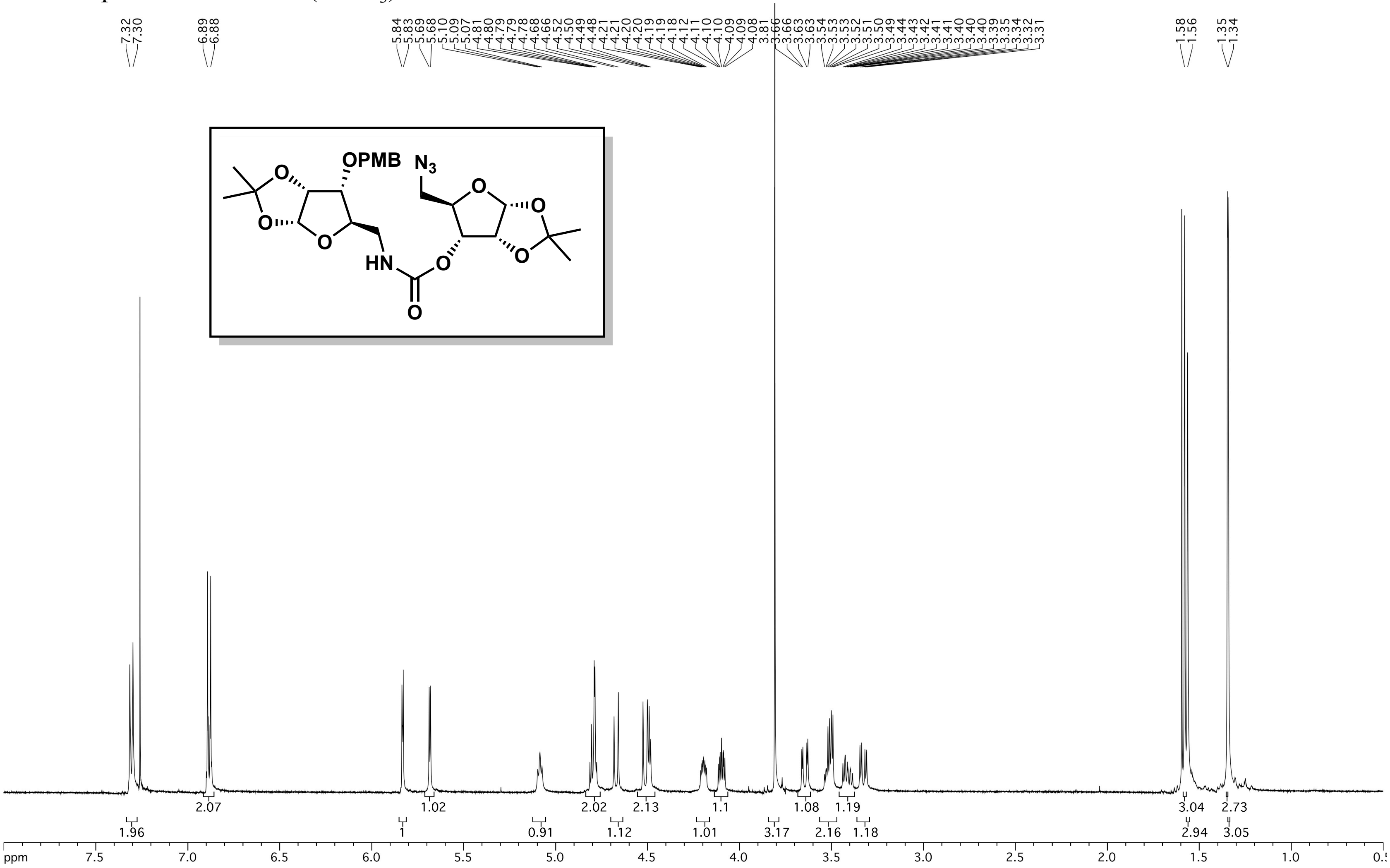

Compound **9a** ^{13}C NMR ($CDCl_3$)

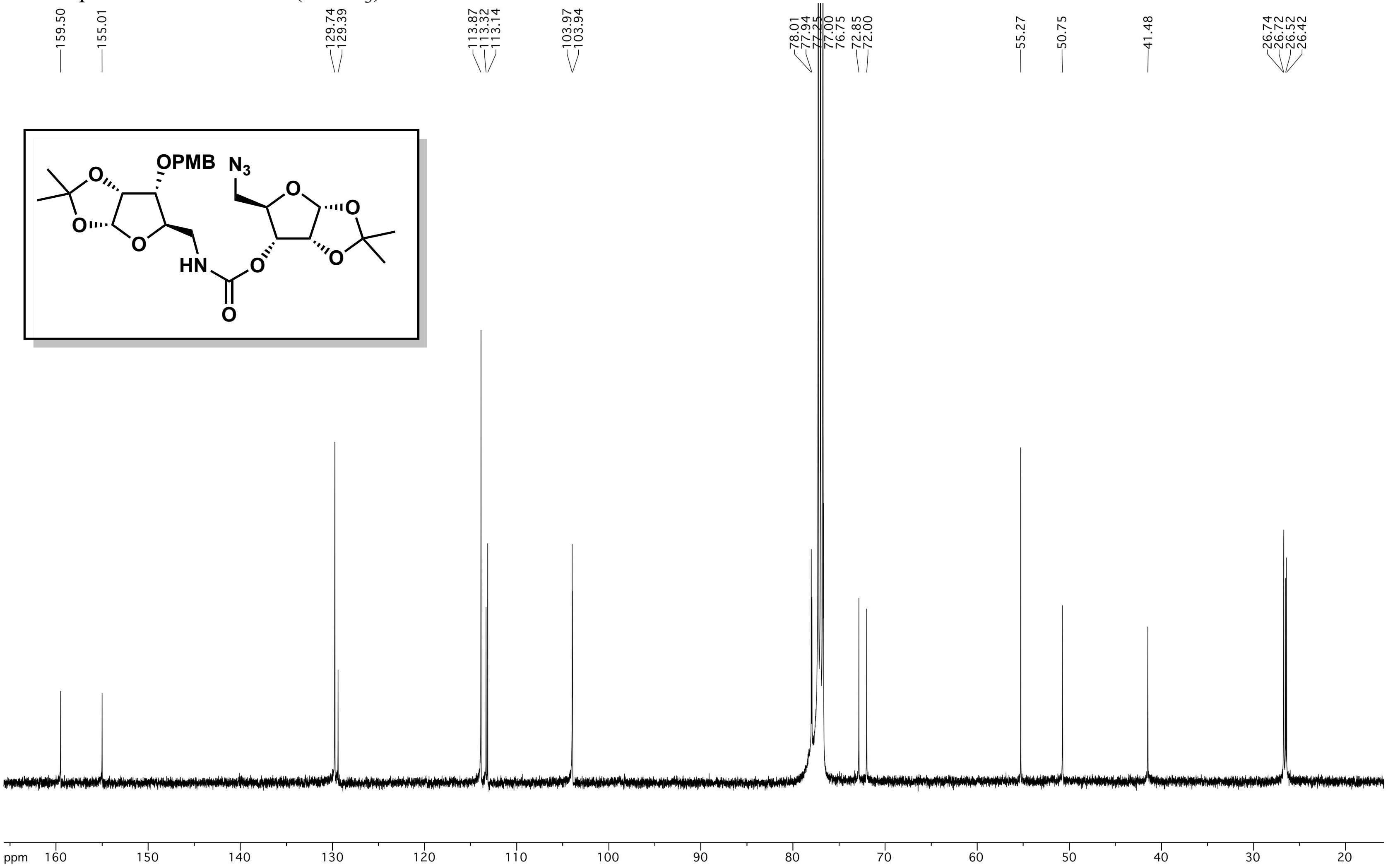

Compound **9a** FTIR

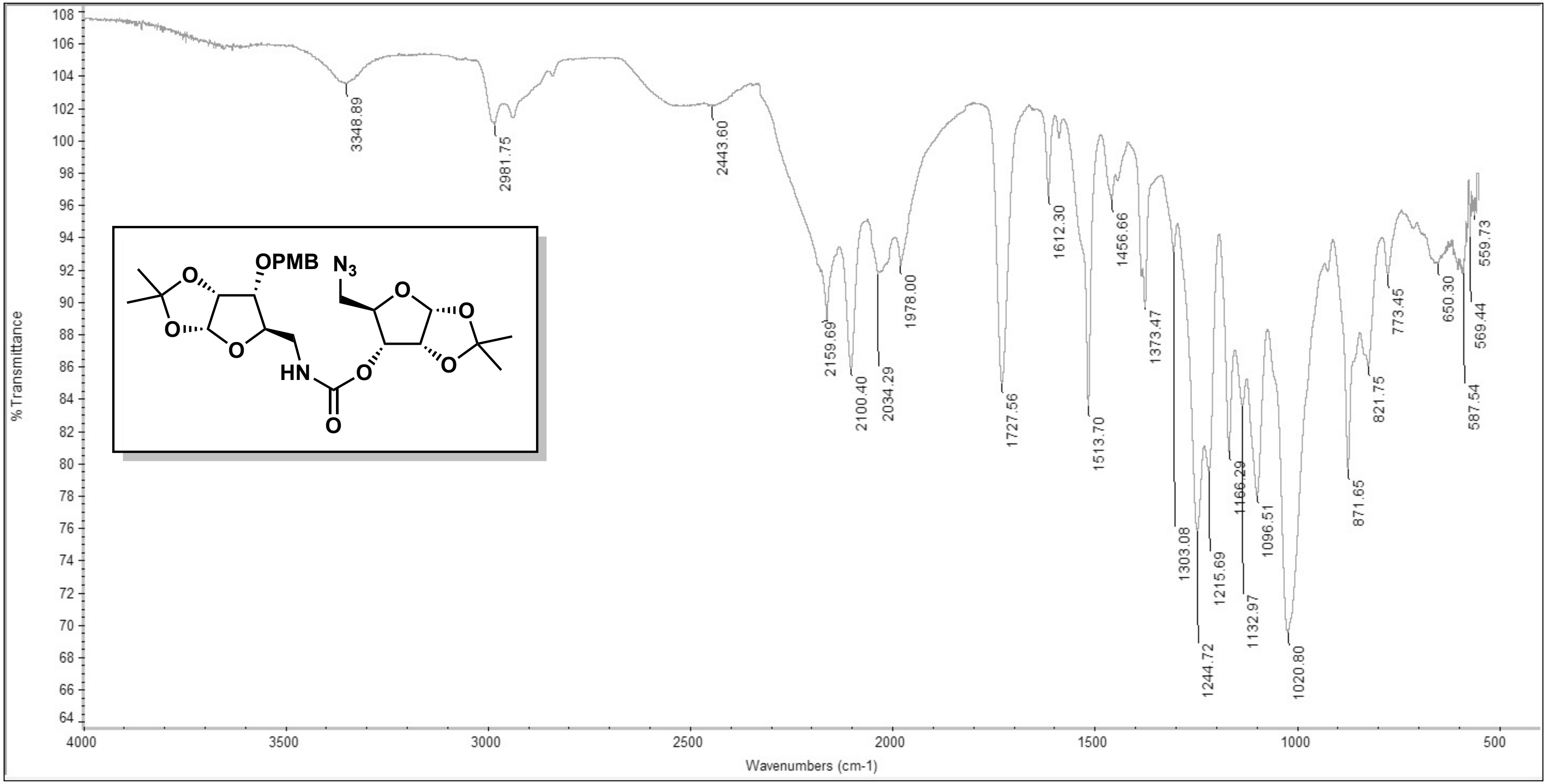

Compound **10a** ^{1}H NMR ($CDCl_3$)

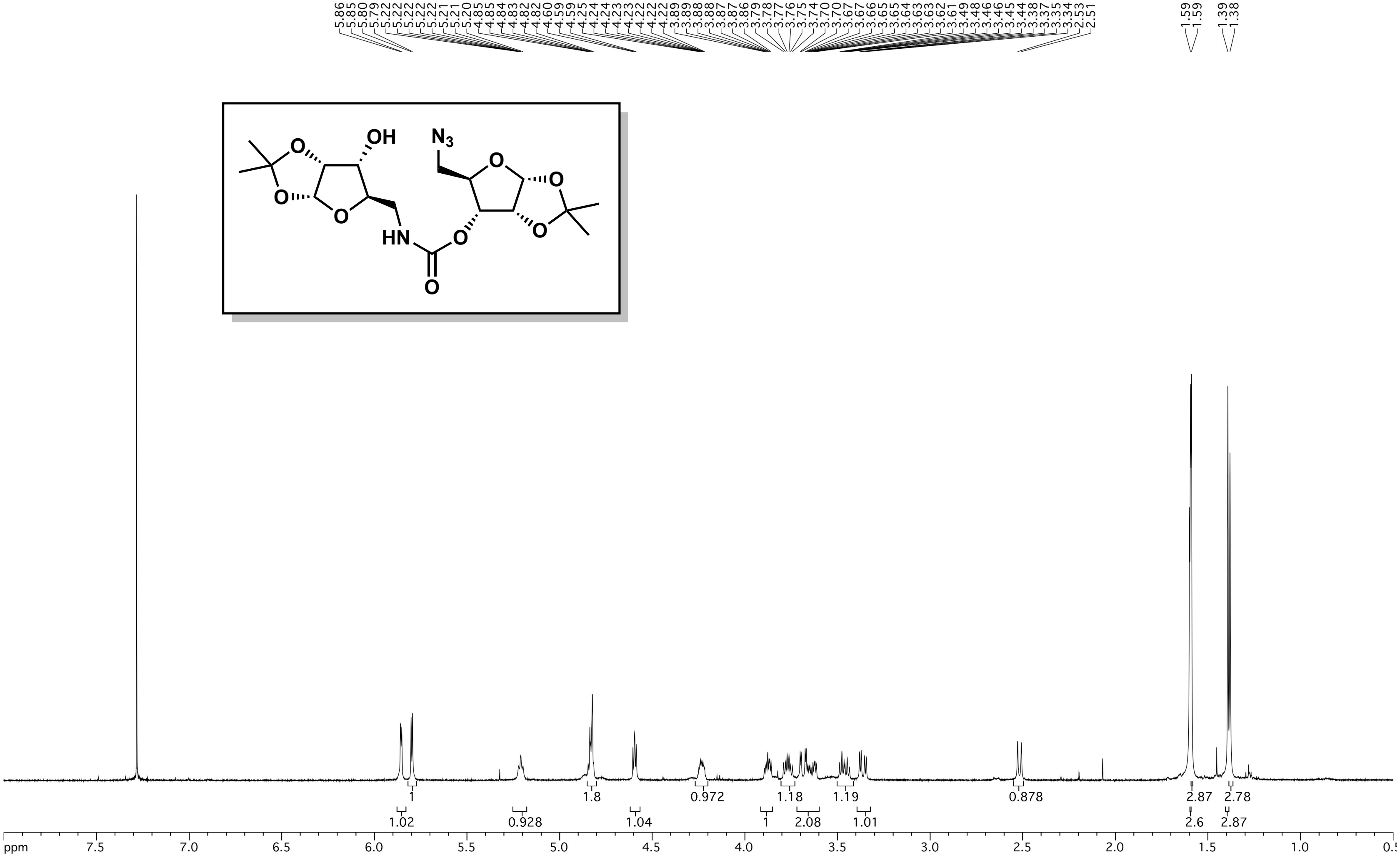

Compound **10a** ^{13}C NMR ($CDCl_3$)

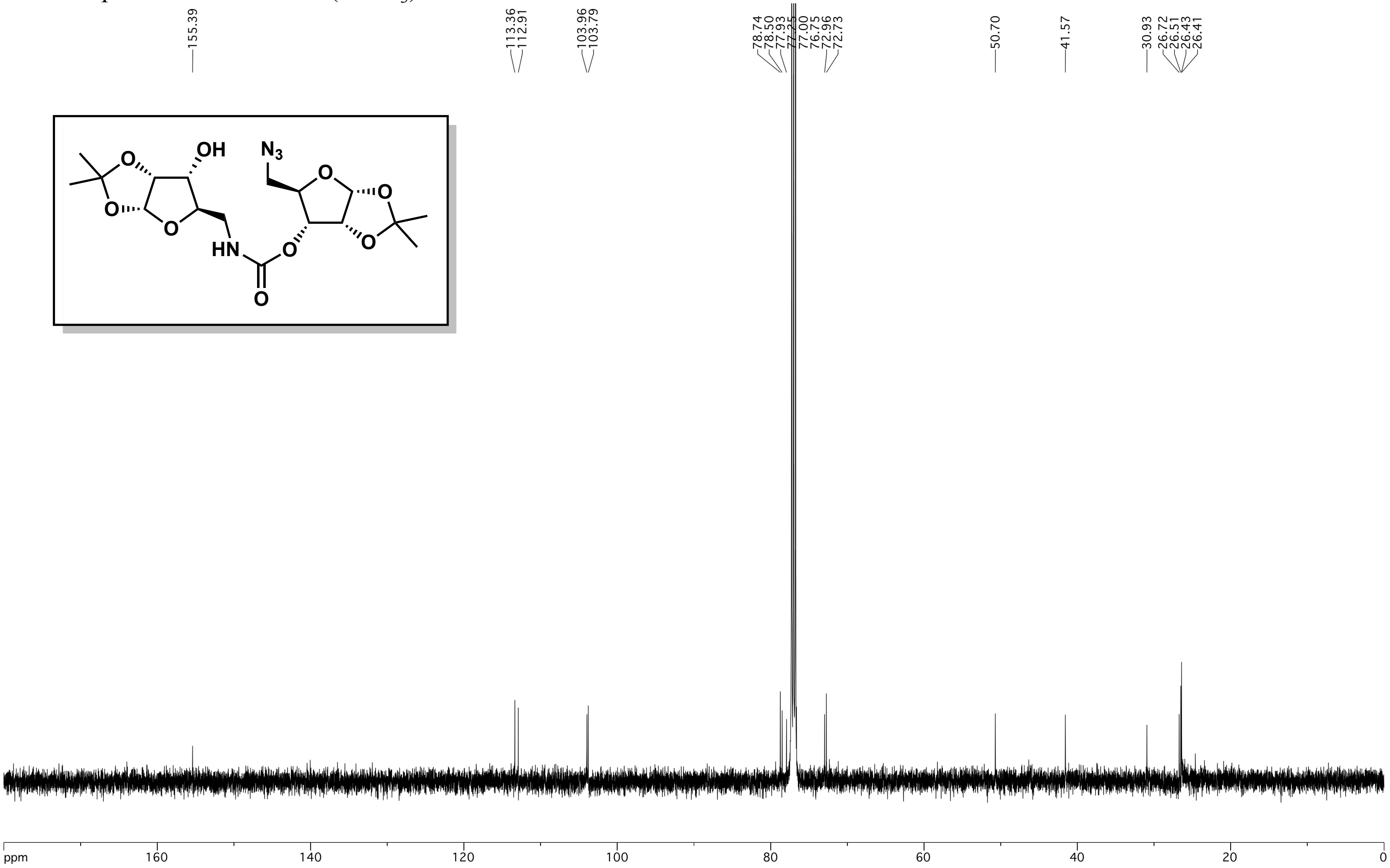

Compound **10a** FTIR

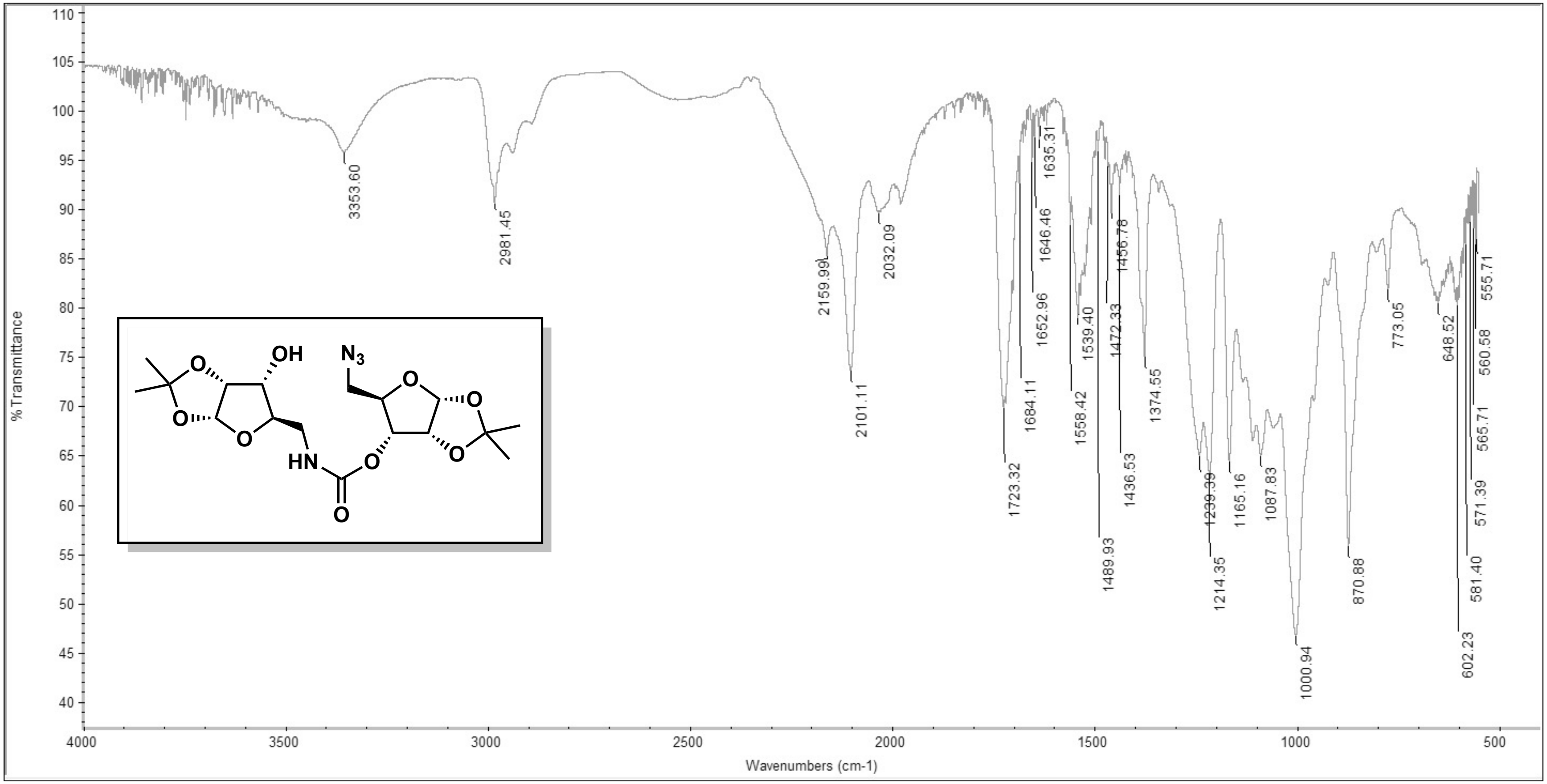

Compound **11a** ^{1}H NMR ($CDCl_3$)

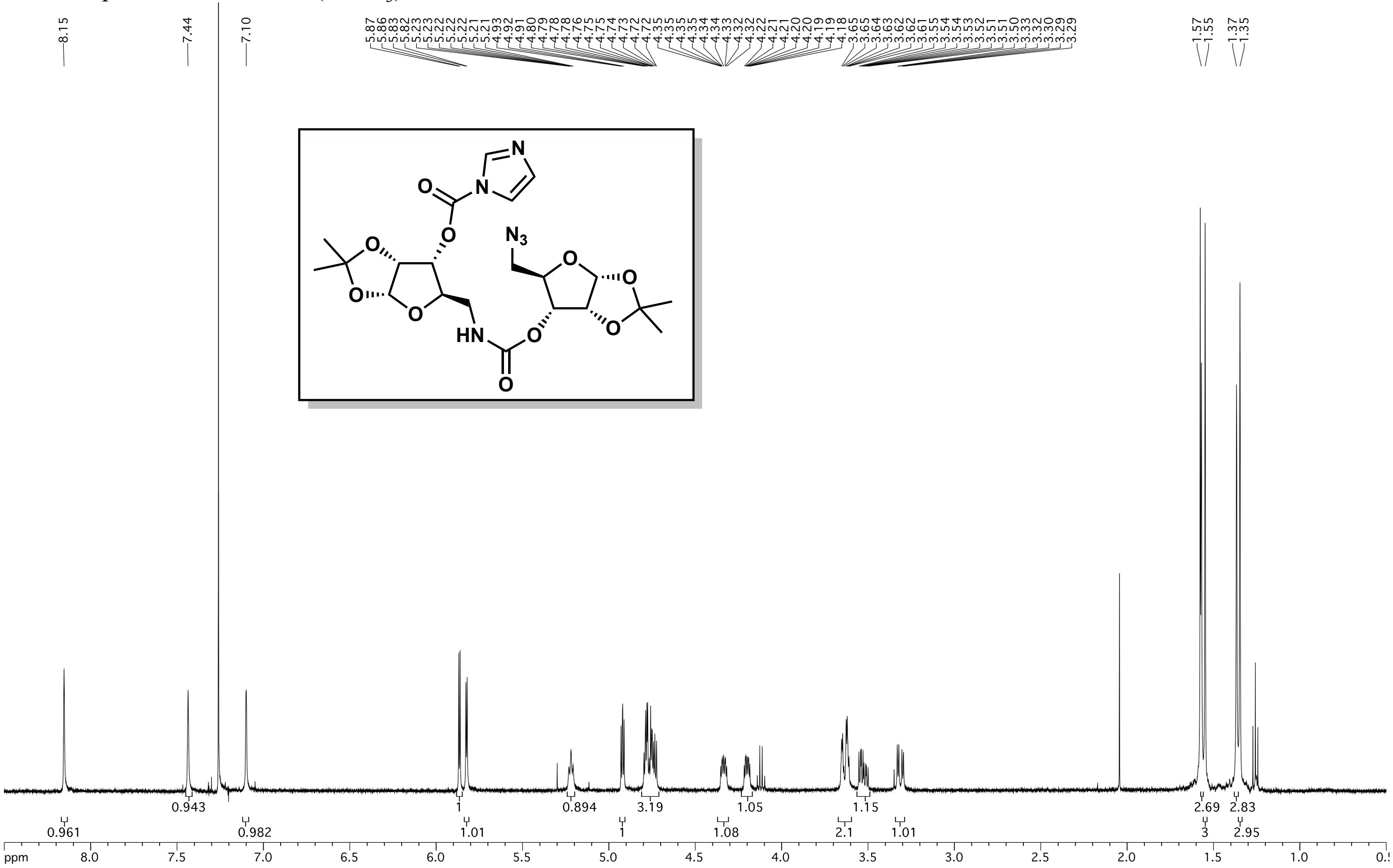

Compound **11a** ^{13}C NMR ($CDCl_3$)

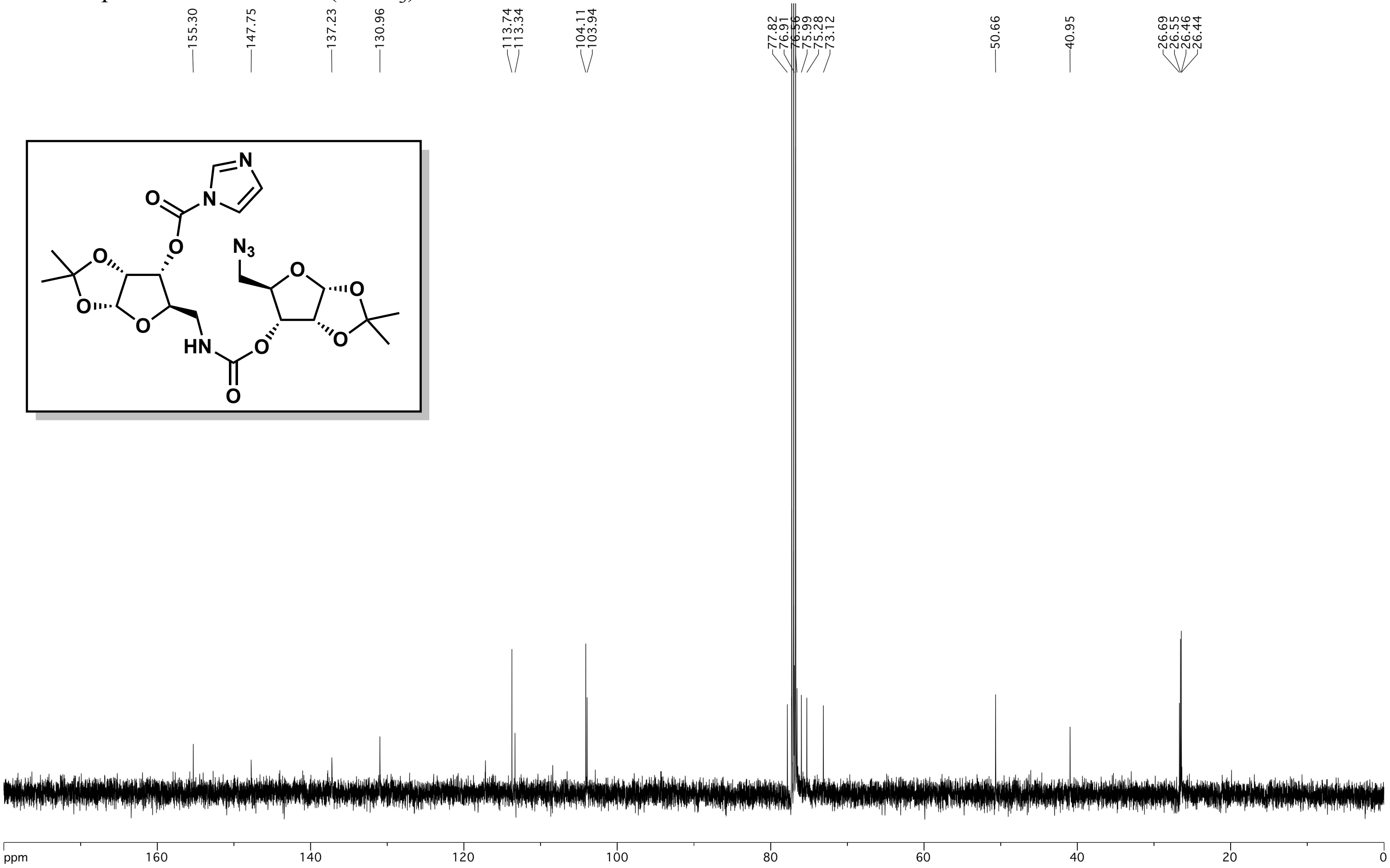

Compound **11a** FTIR

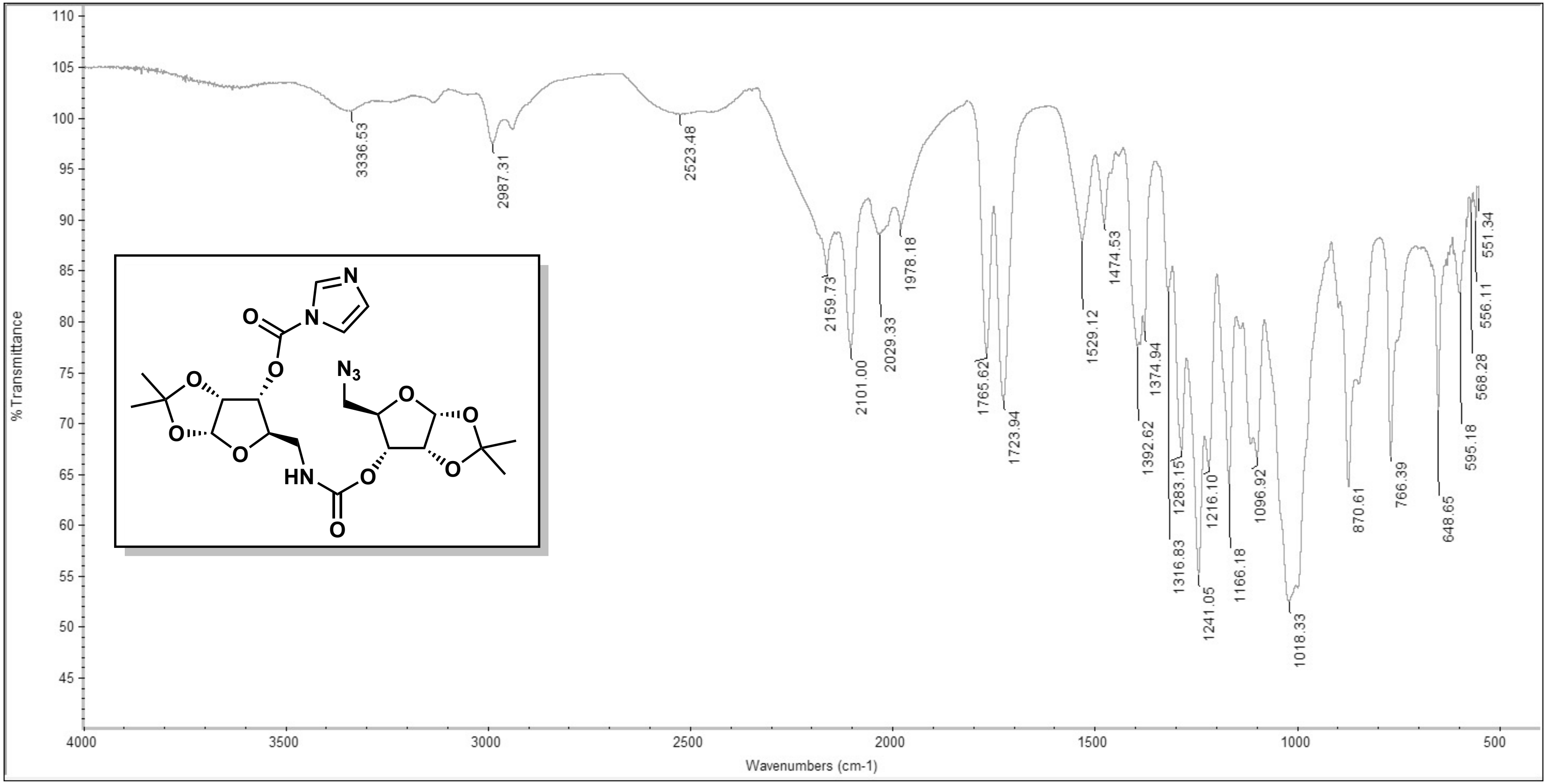

Compound **13a** ^{1}H NMR ($CDCl_3$)

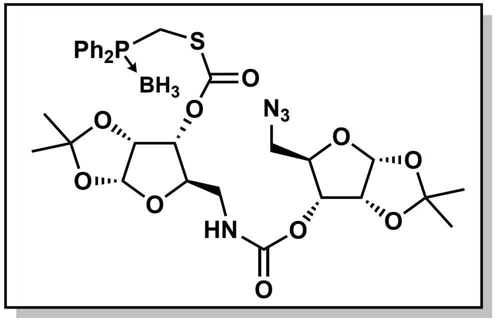

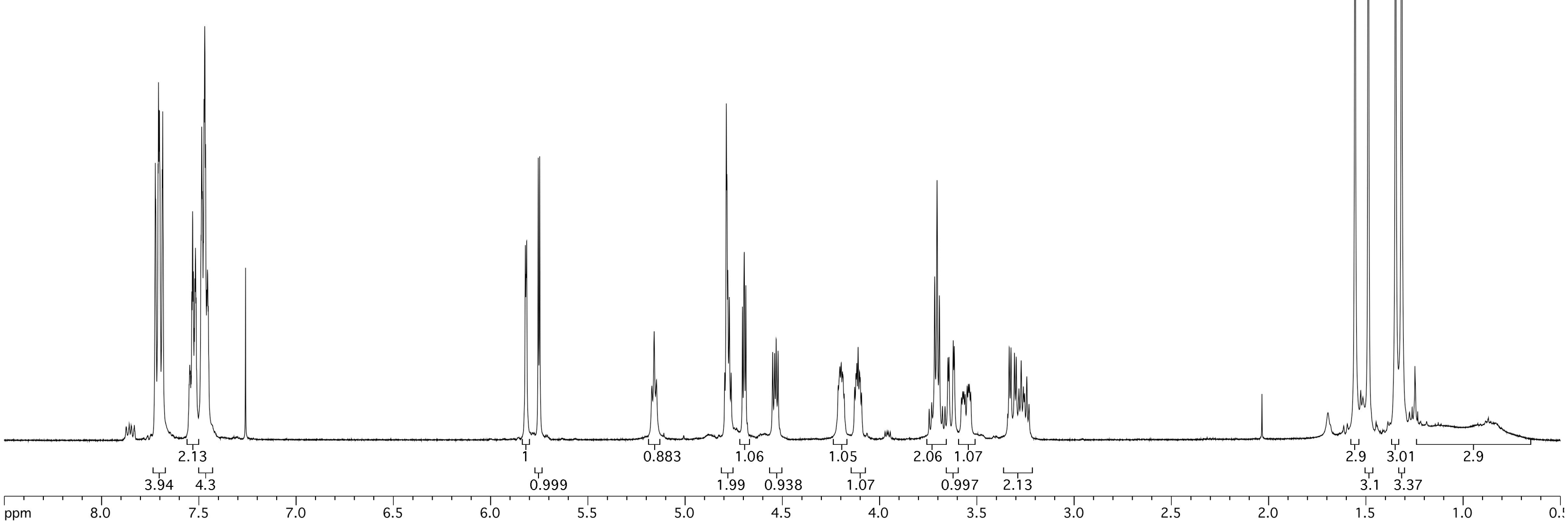

Compound **13a** ^{13}C NMR ($CDCl_3$)

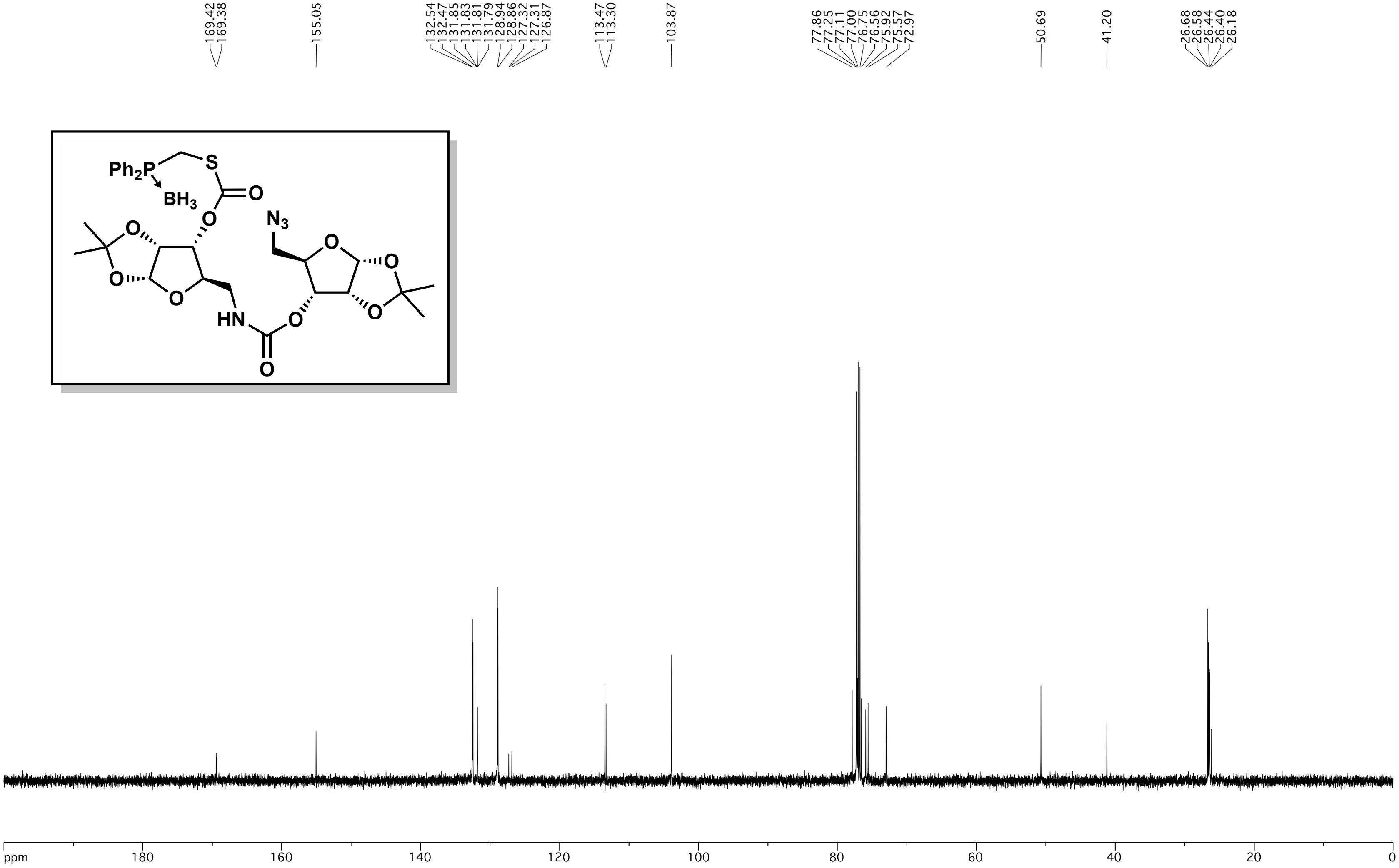

Compound **13a** FTIR

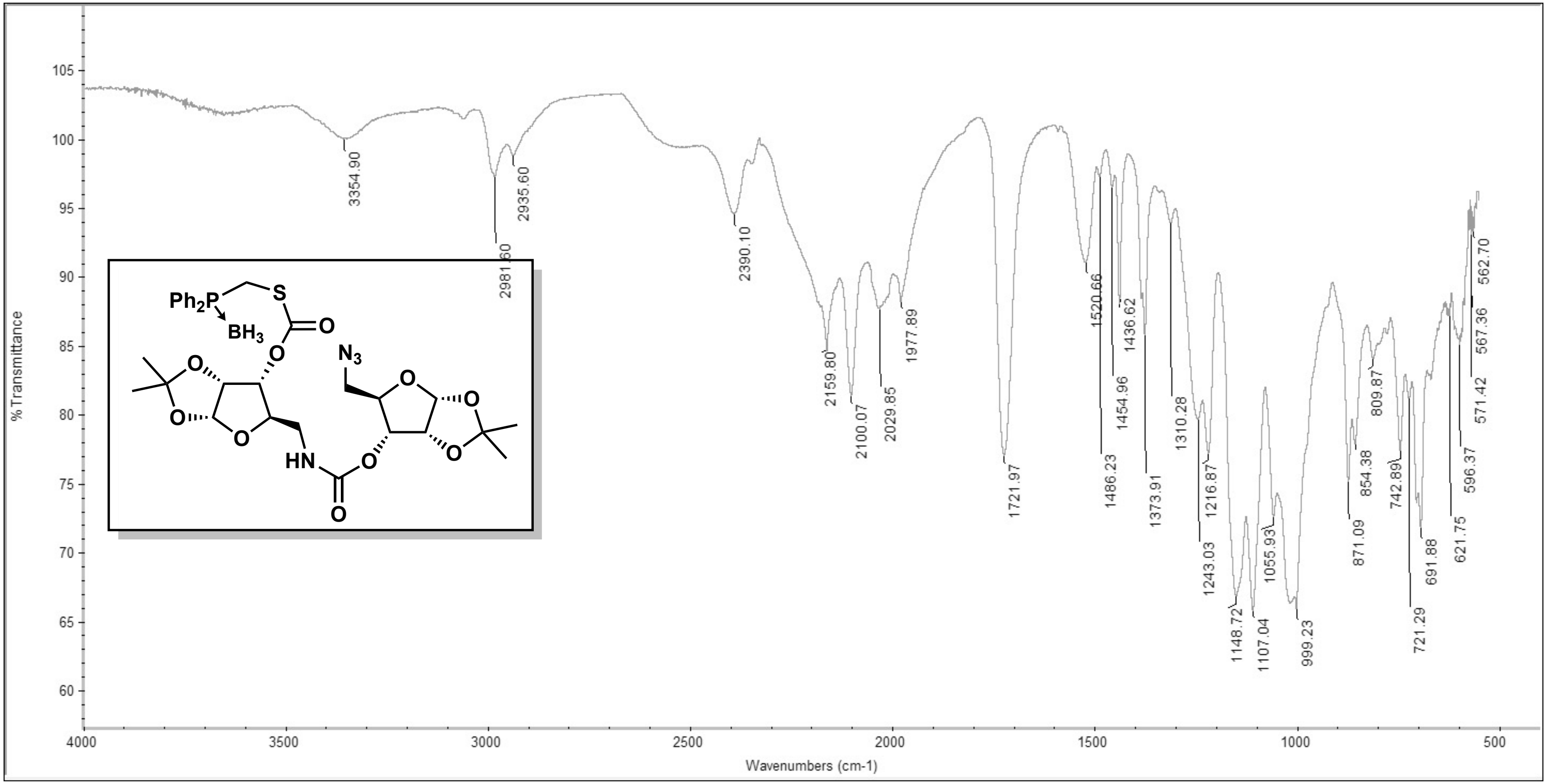

Compound **15a** ^{1}H NMR ($CDCl_3$)

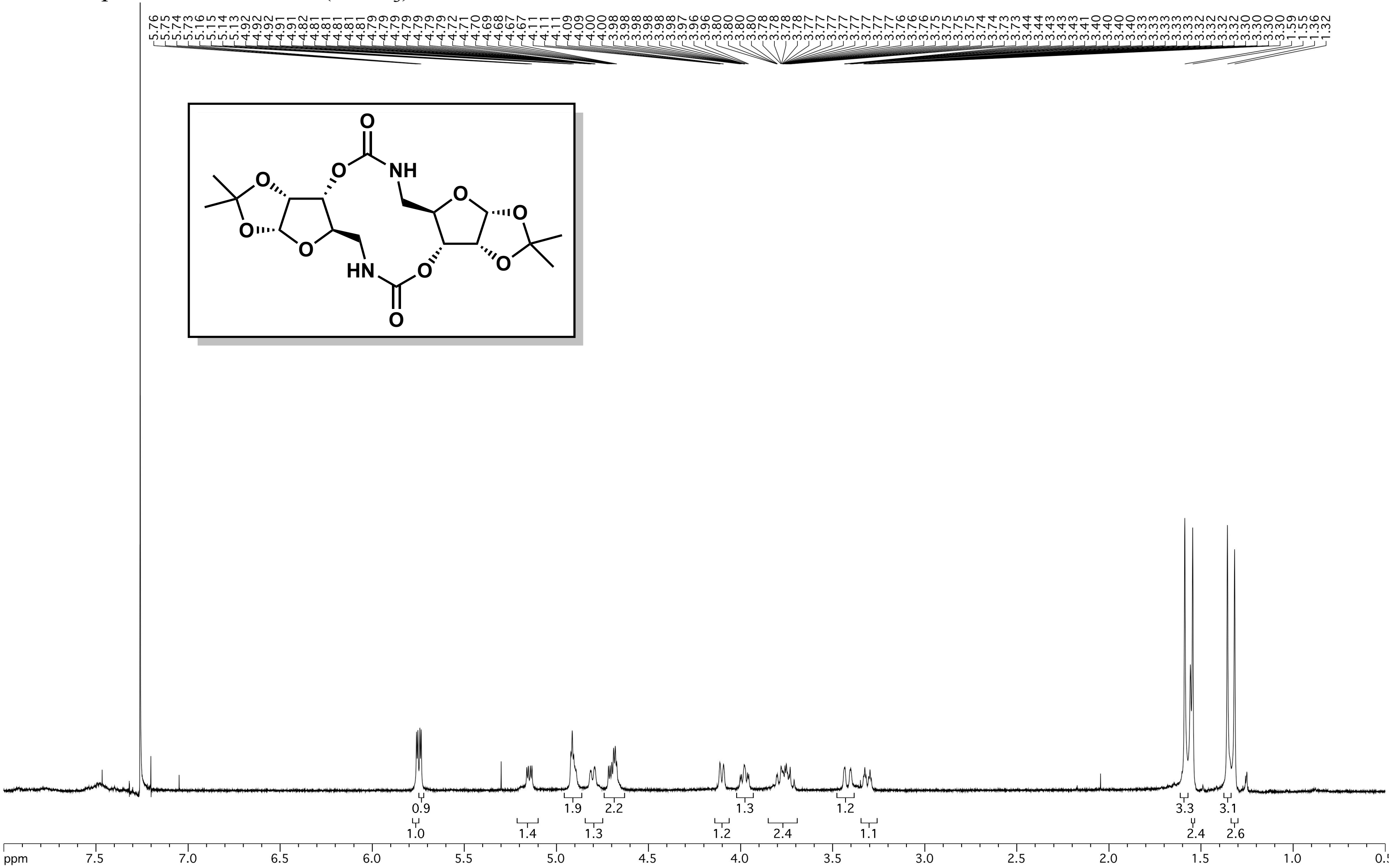

Compound **15a** ^{13}C NMR ($CDCl_3$)

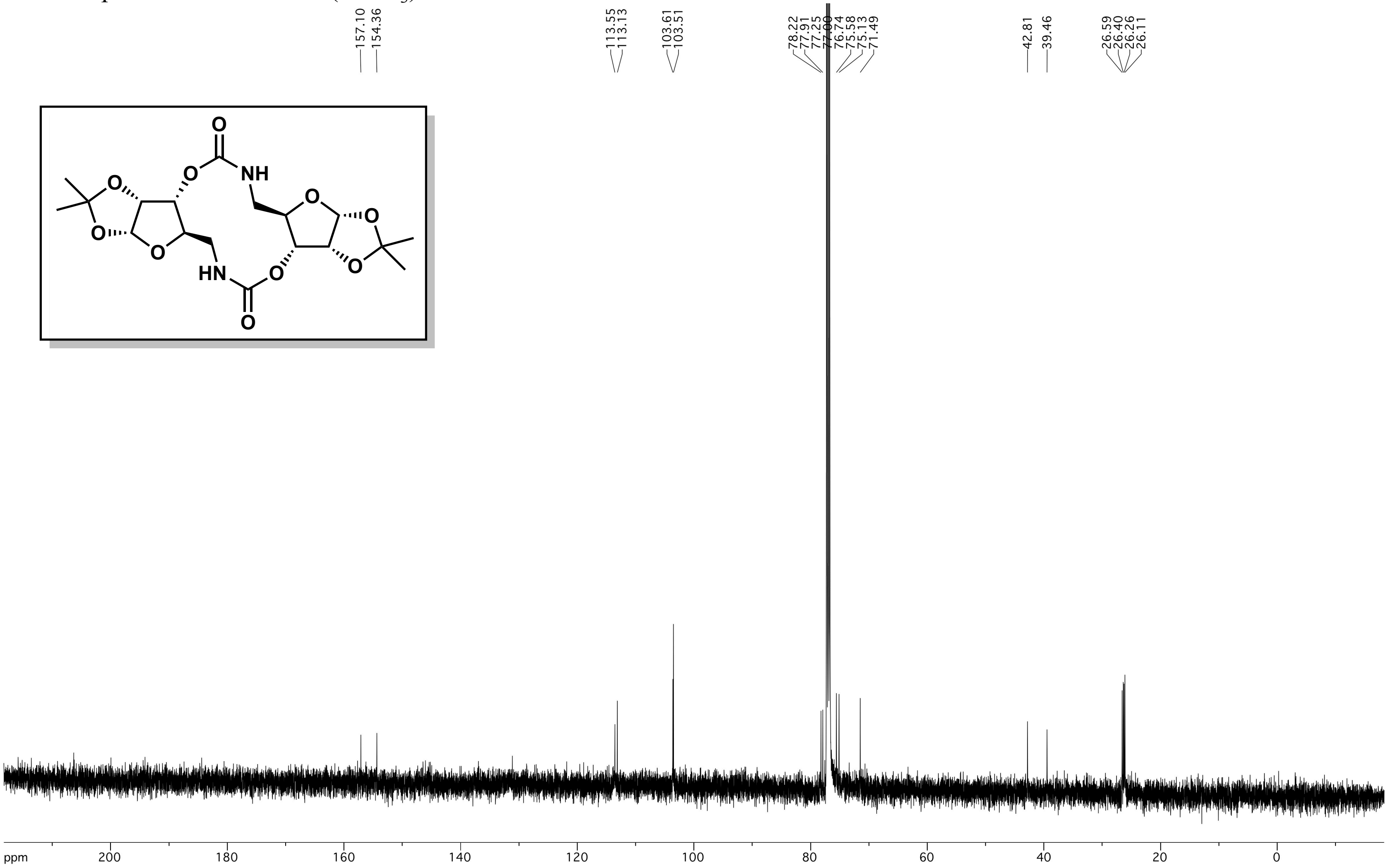

Compound **15a** FTIR

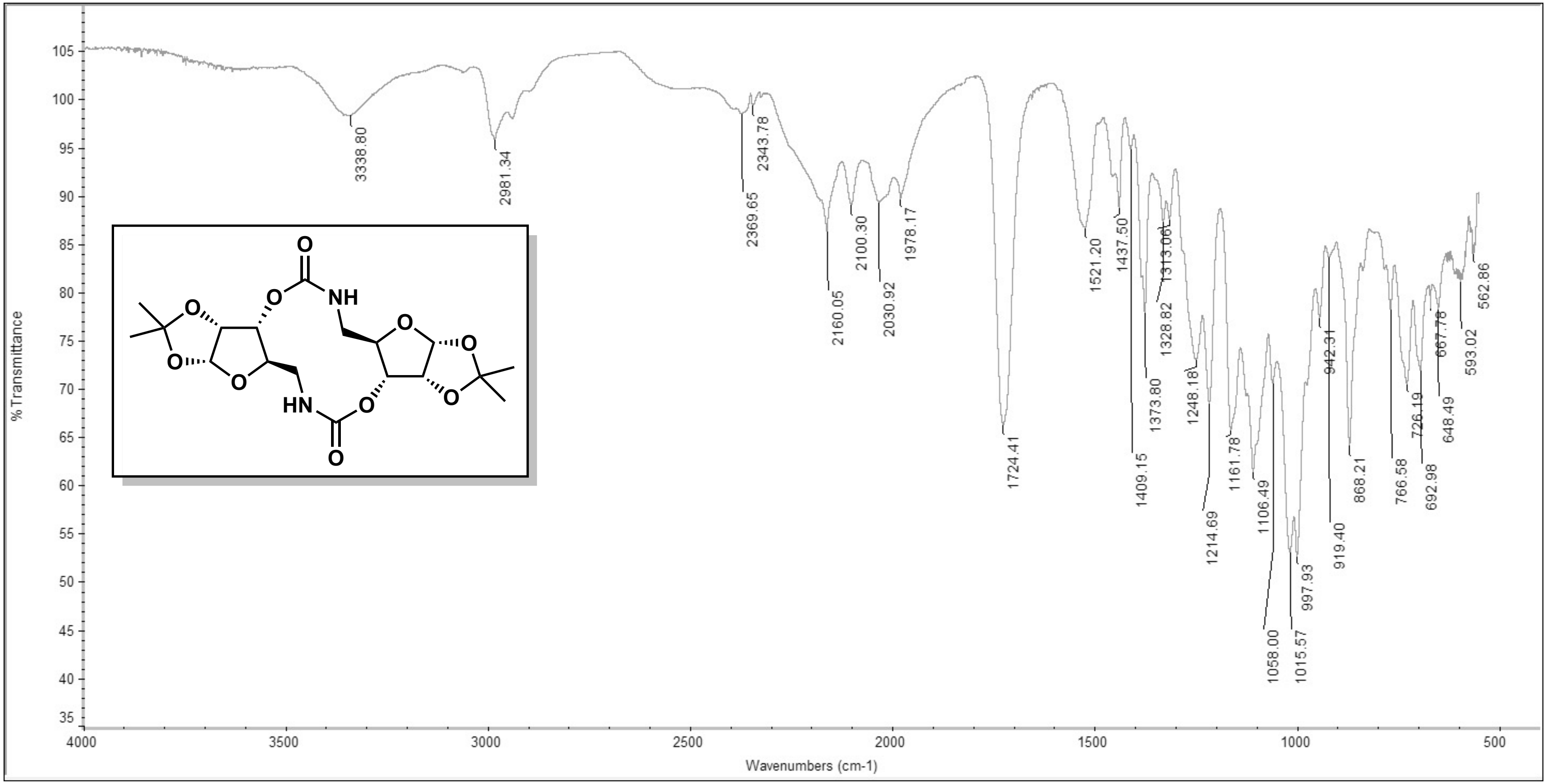

Compound **5a** ^{1}H NMR ($CDCl_3$)

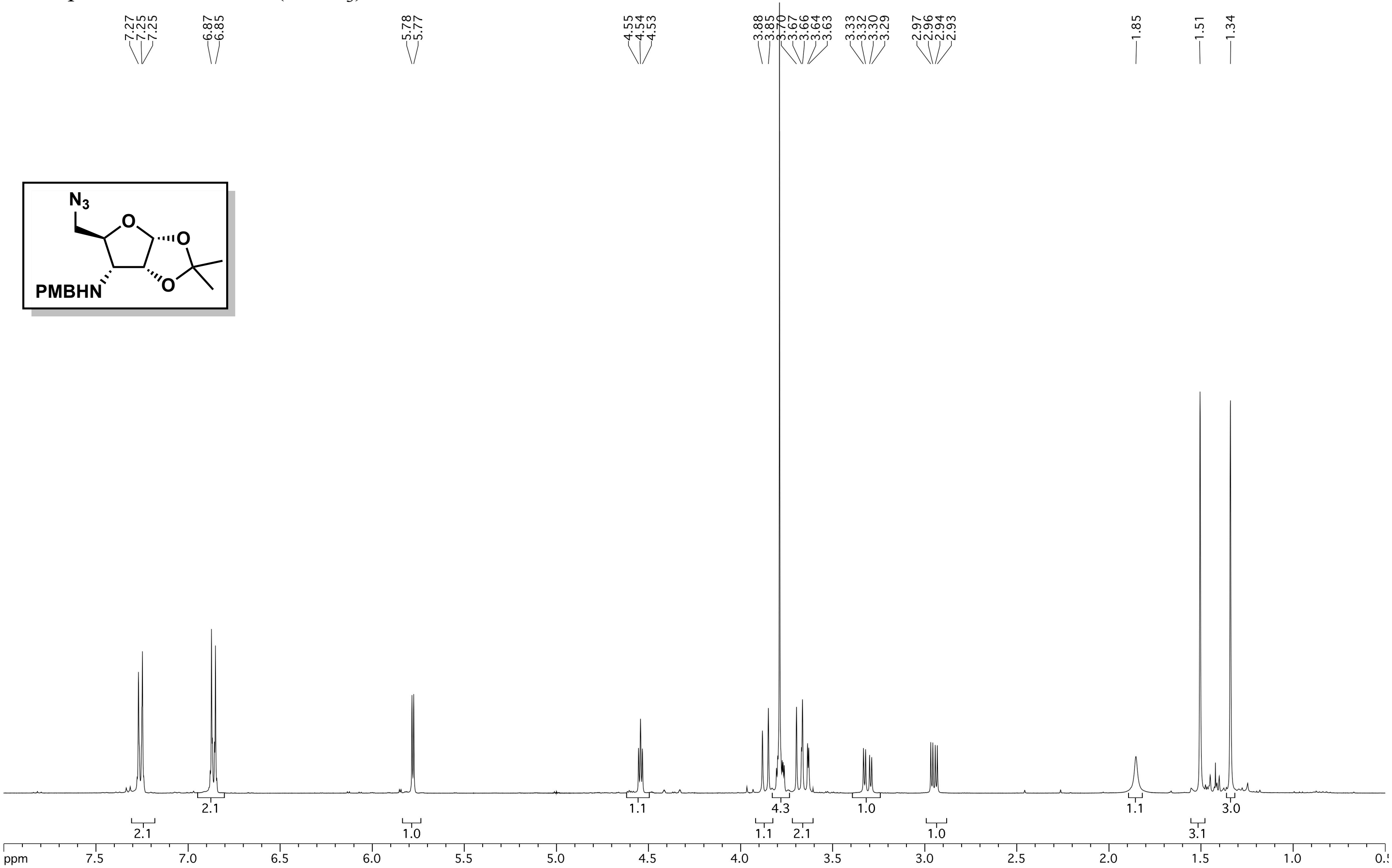

Compound **5a** ^{13}C NMR ($CDCl_3$)

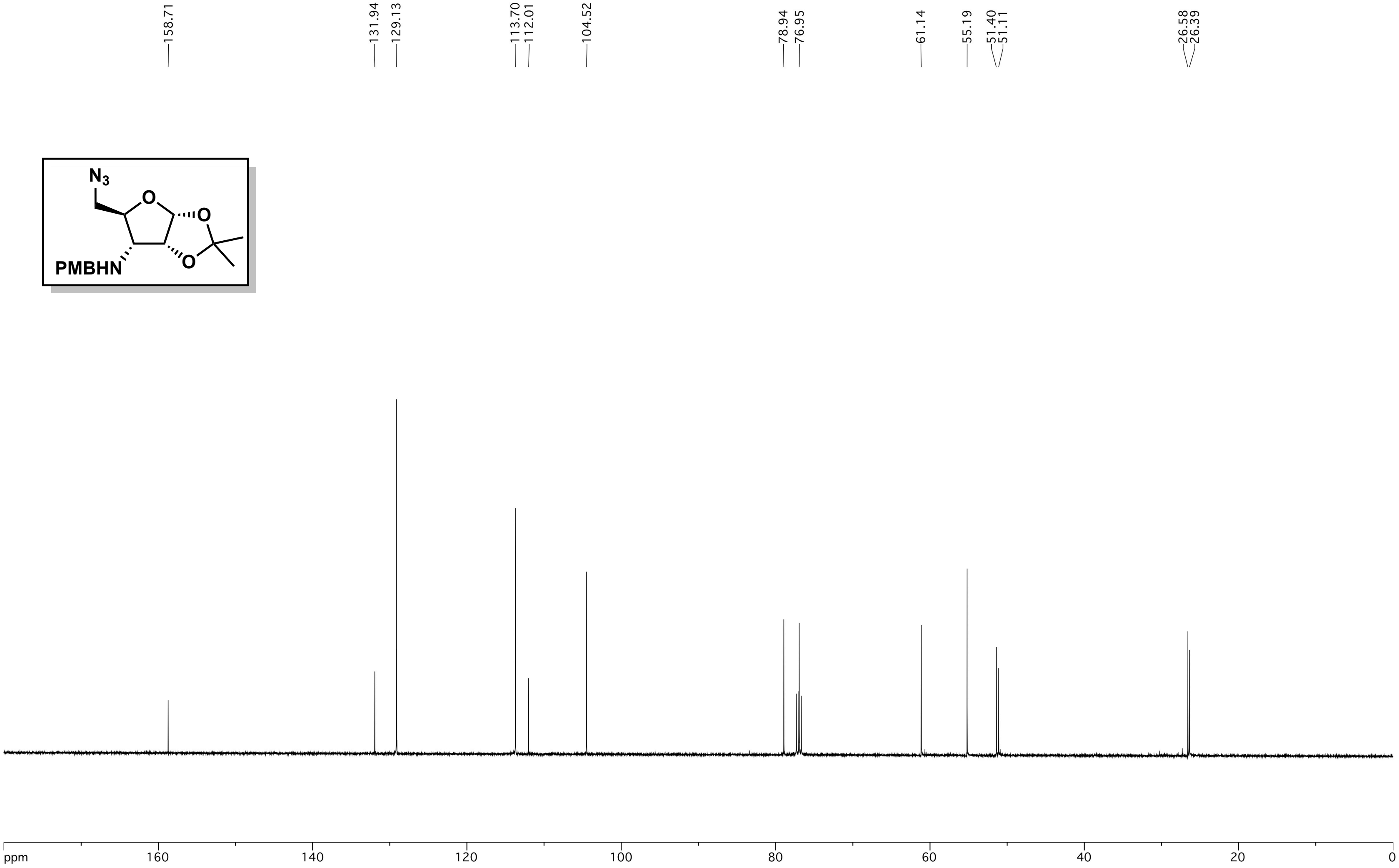

Compound **5a** FTIR

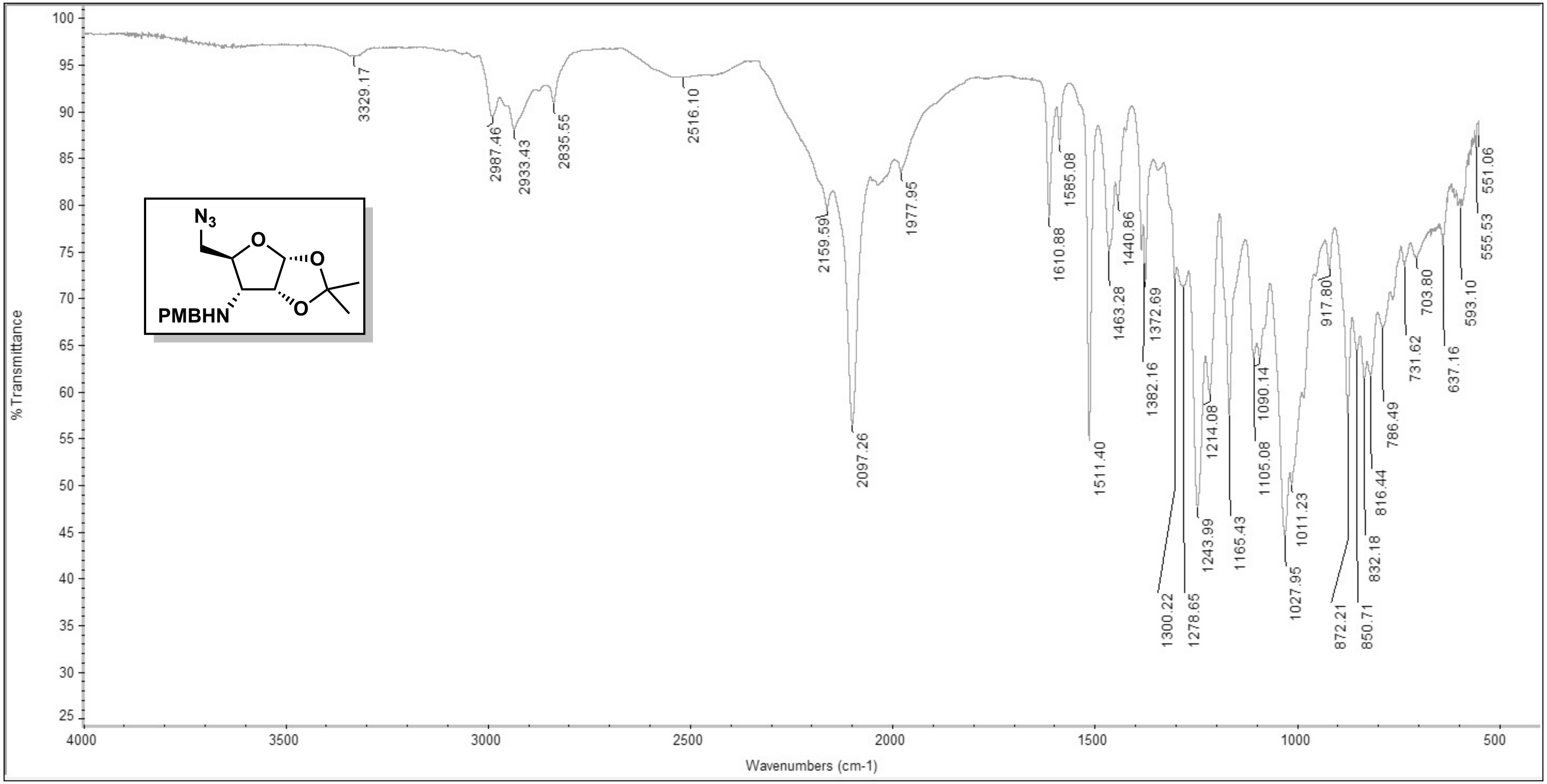

Compound **5b** ^{1}H NMR ($CDCl_3$)

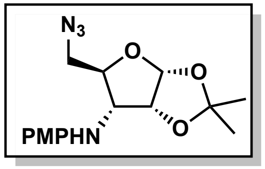

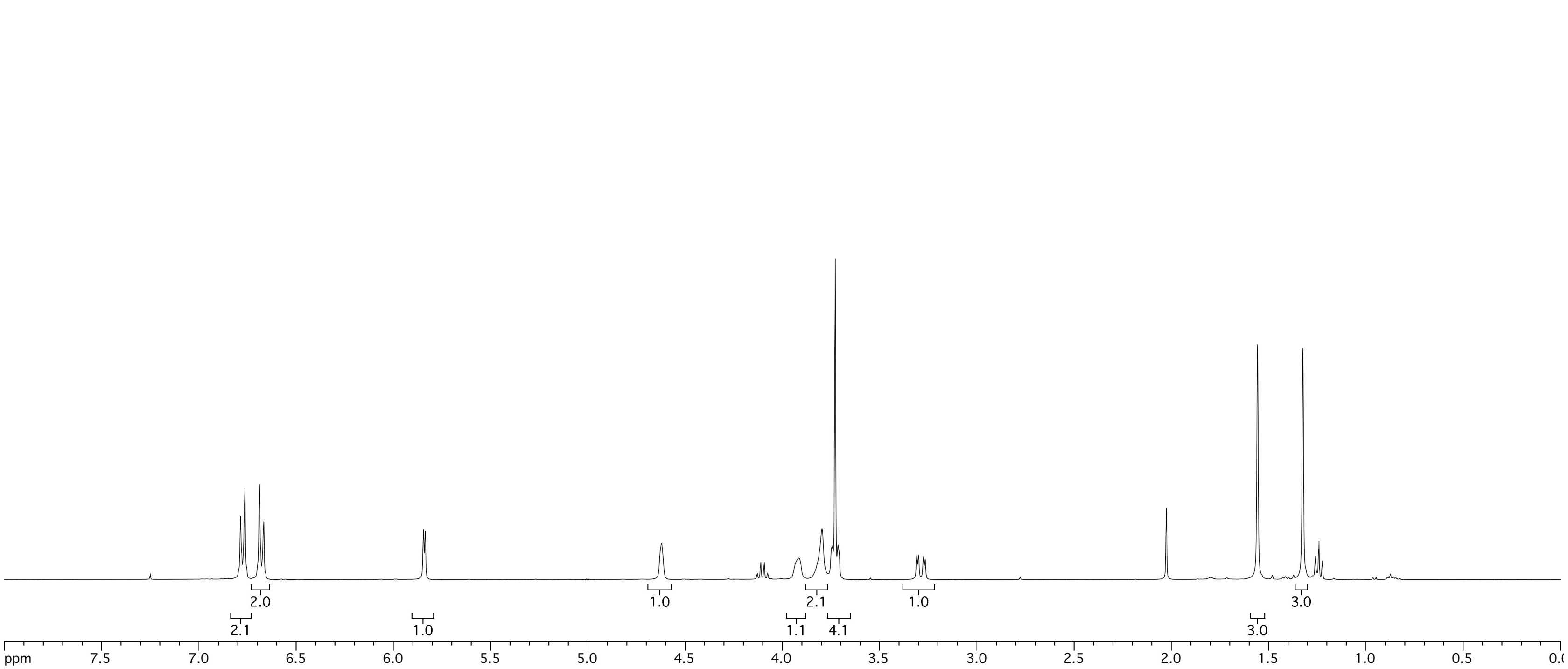

Compound **5b** ^{13}C NMR ($CDCl_3$)

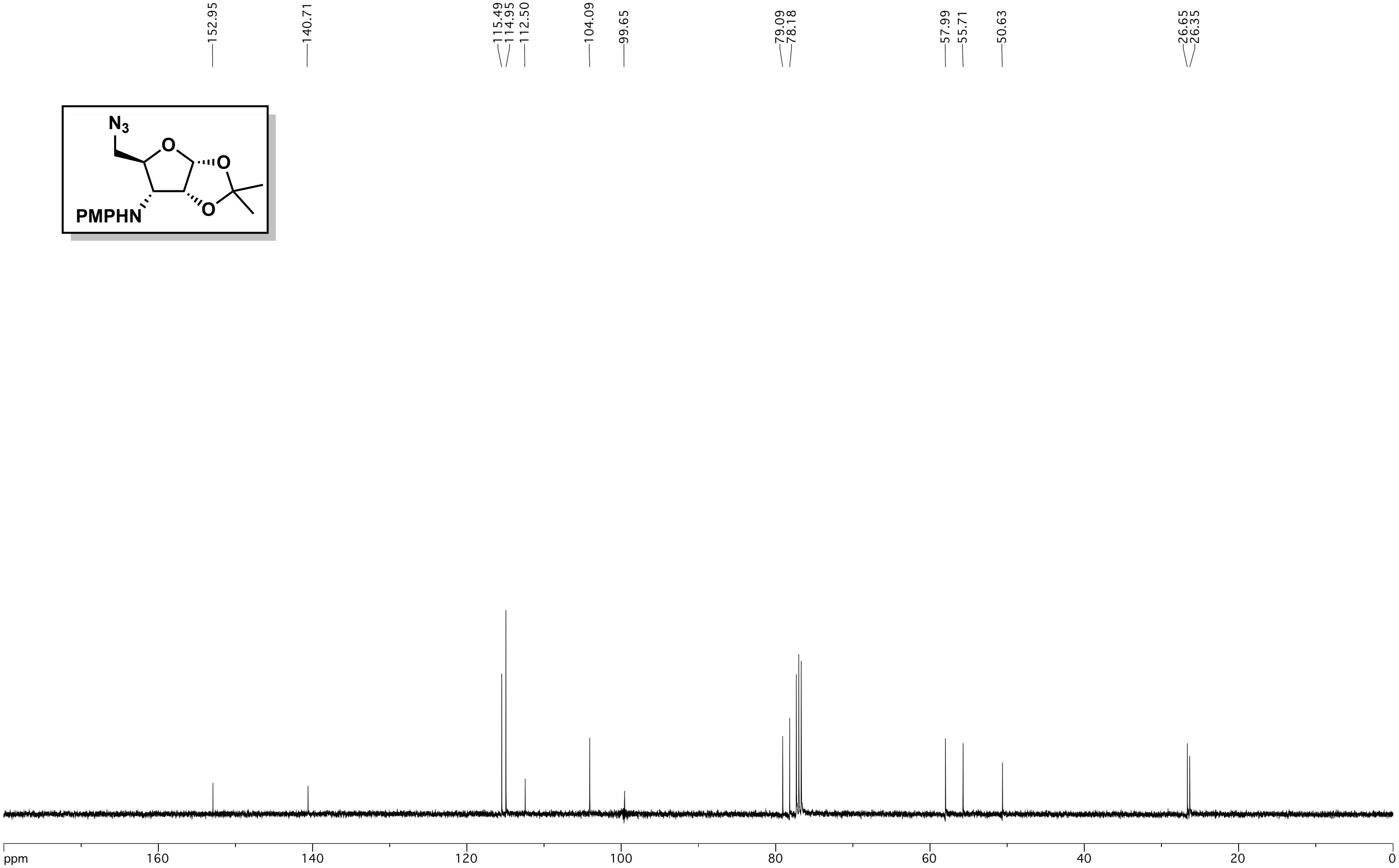

Compound **5b** FTIR

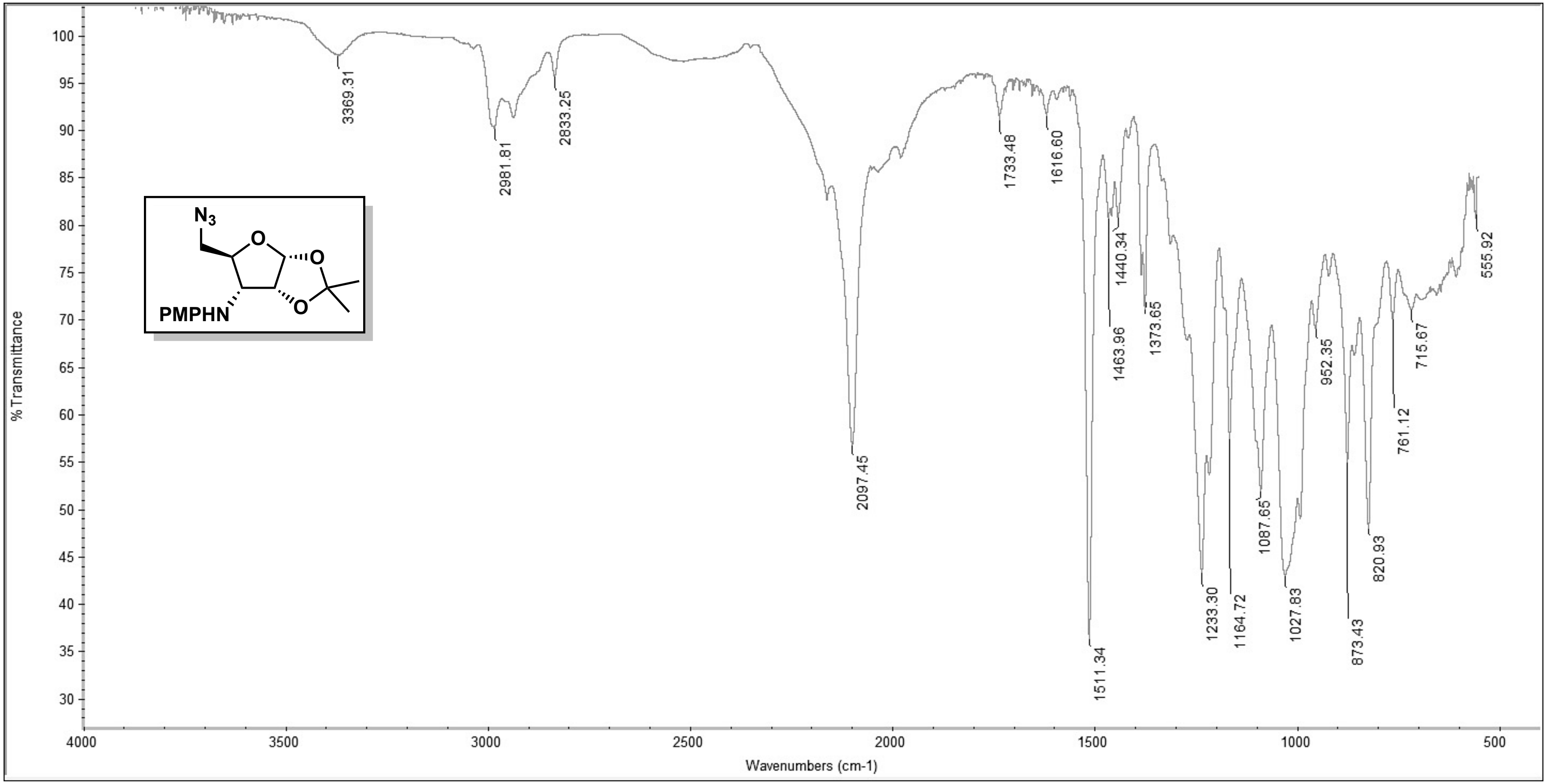

Compound **6** ^{1}H NMR ($CDCl_3$)

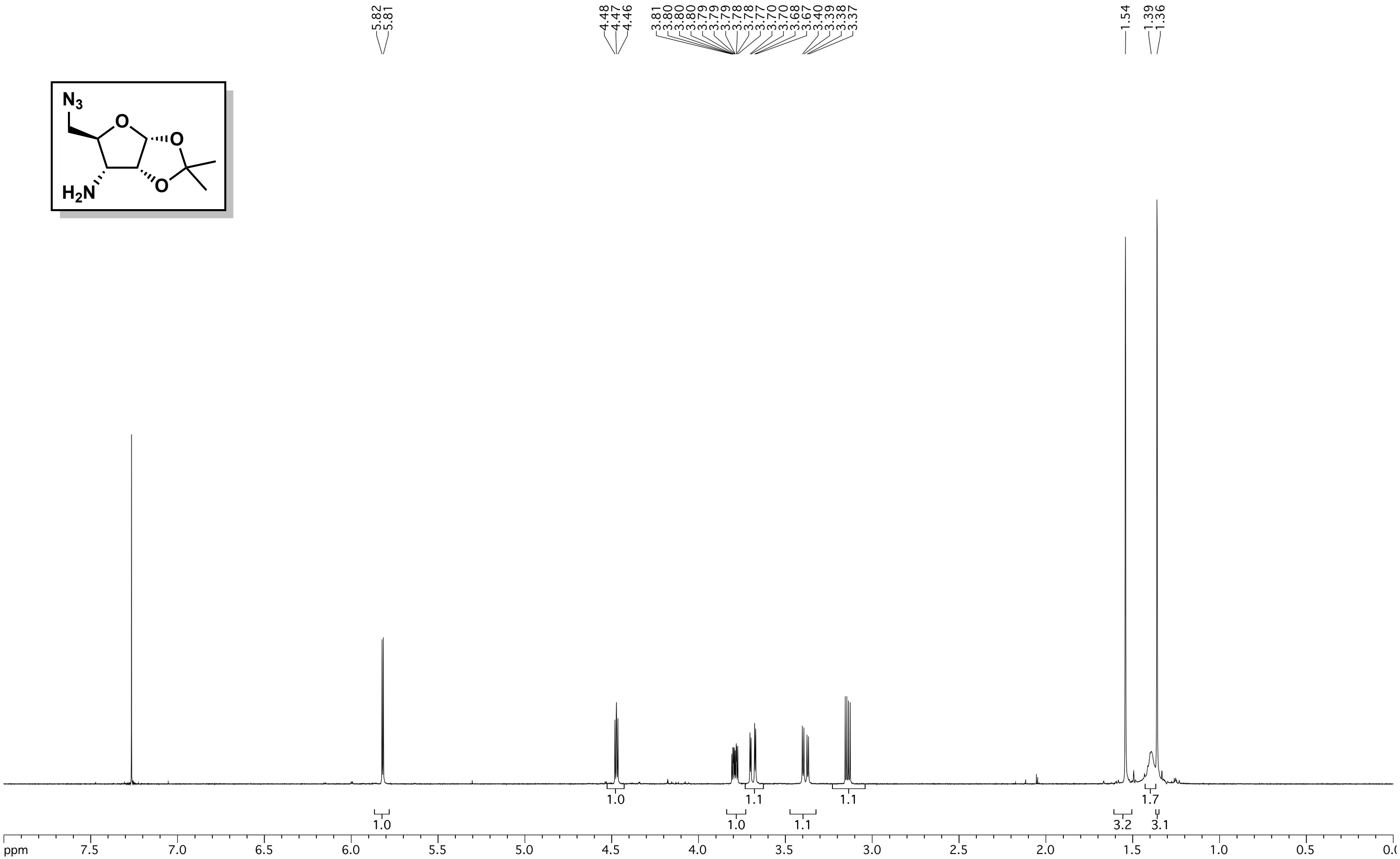

Compound **6** ^{13}C NMR ($CDCl_3$)

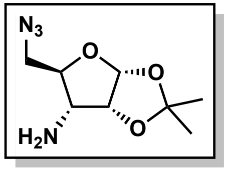

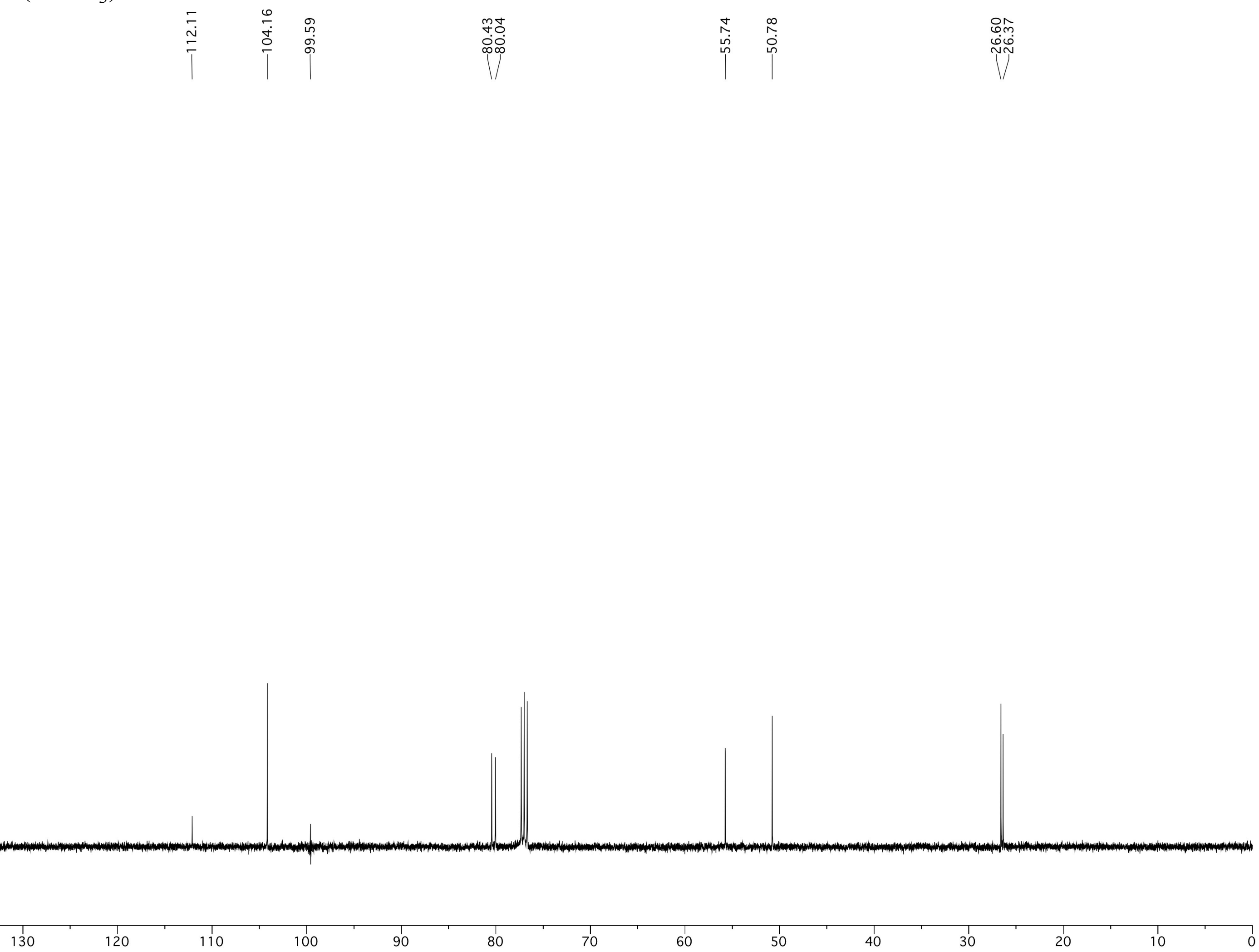

Compound **6** FTIR

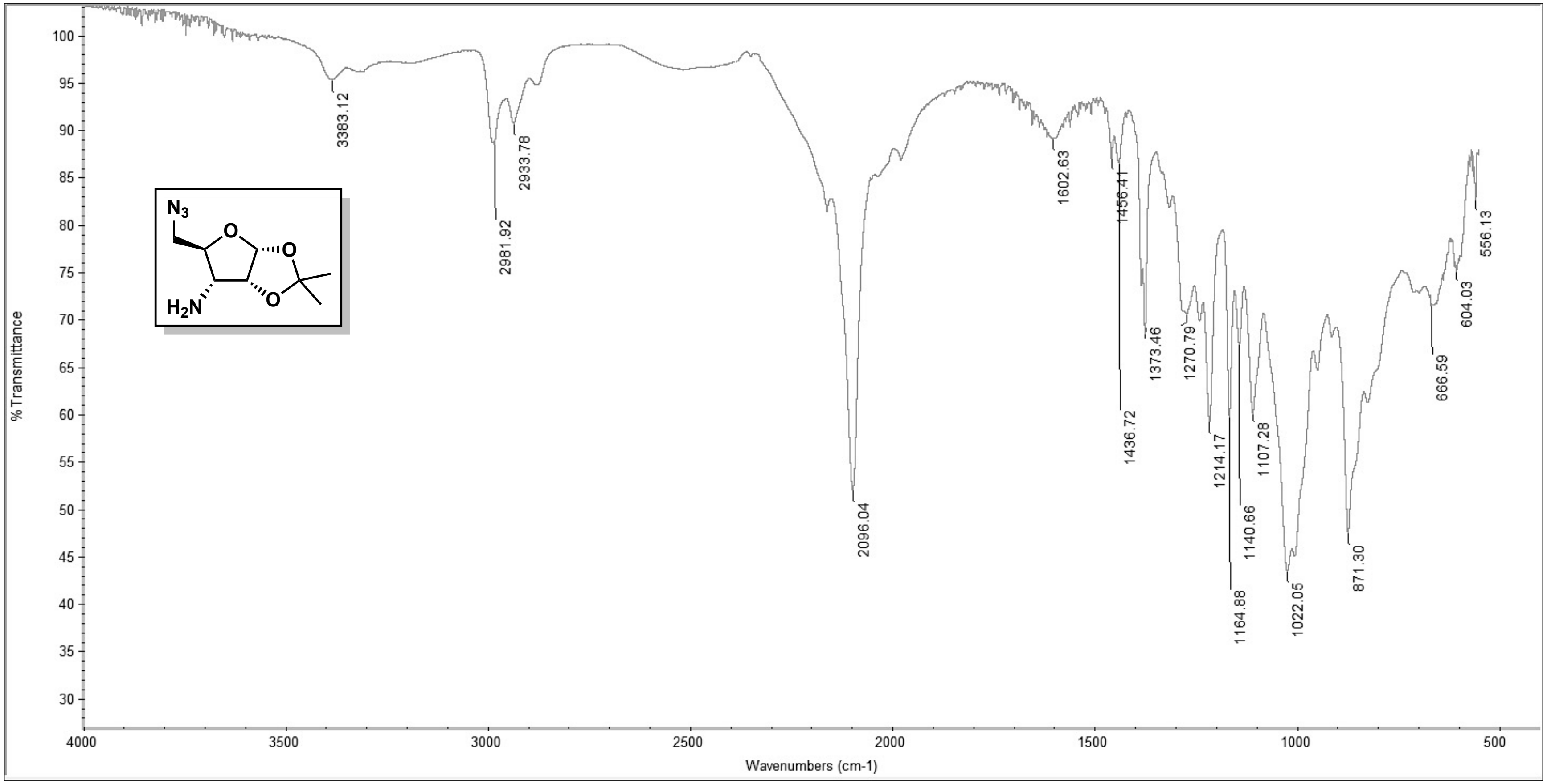

Compound **7** ^{1}H NMR ($CDCl_3$)

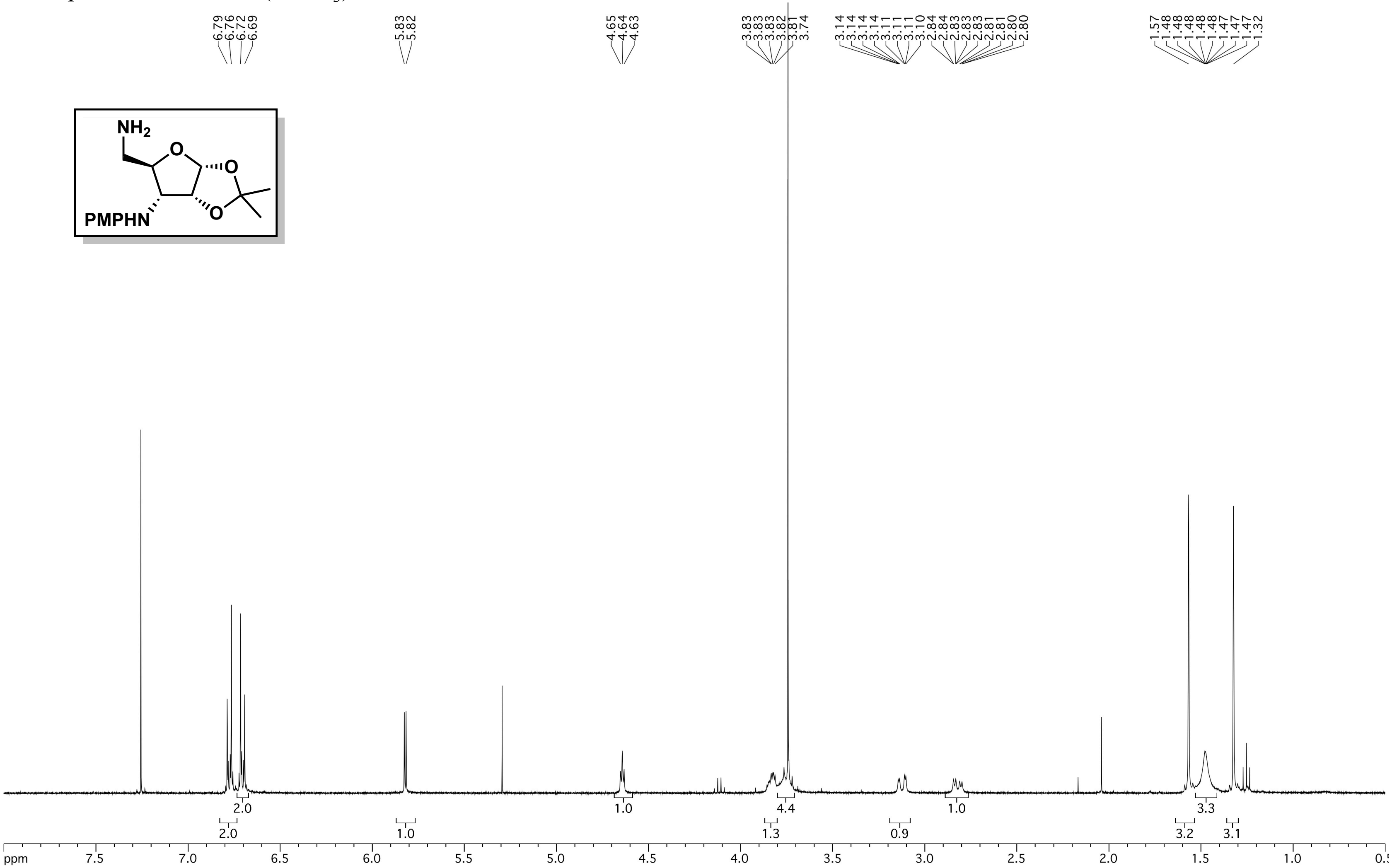

Compound **7** ^{13}C NMR ($CDCl_3$)

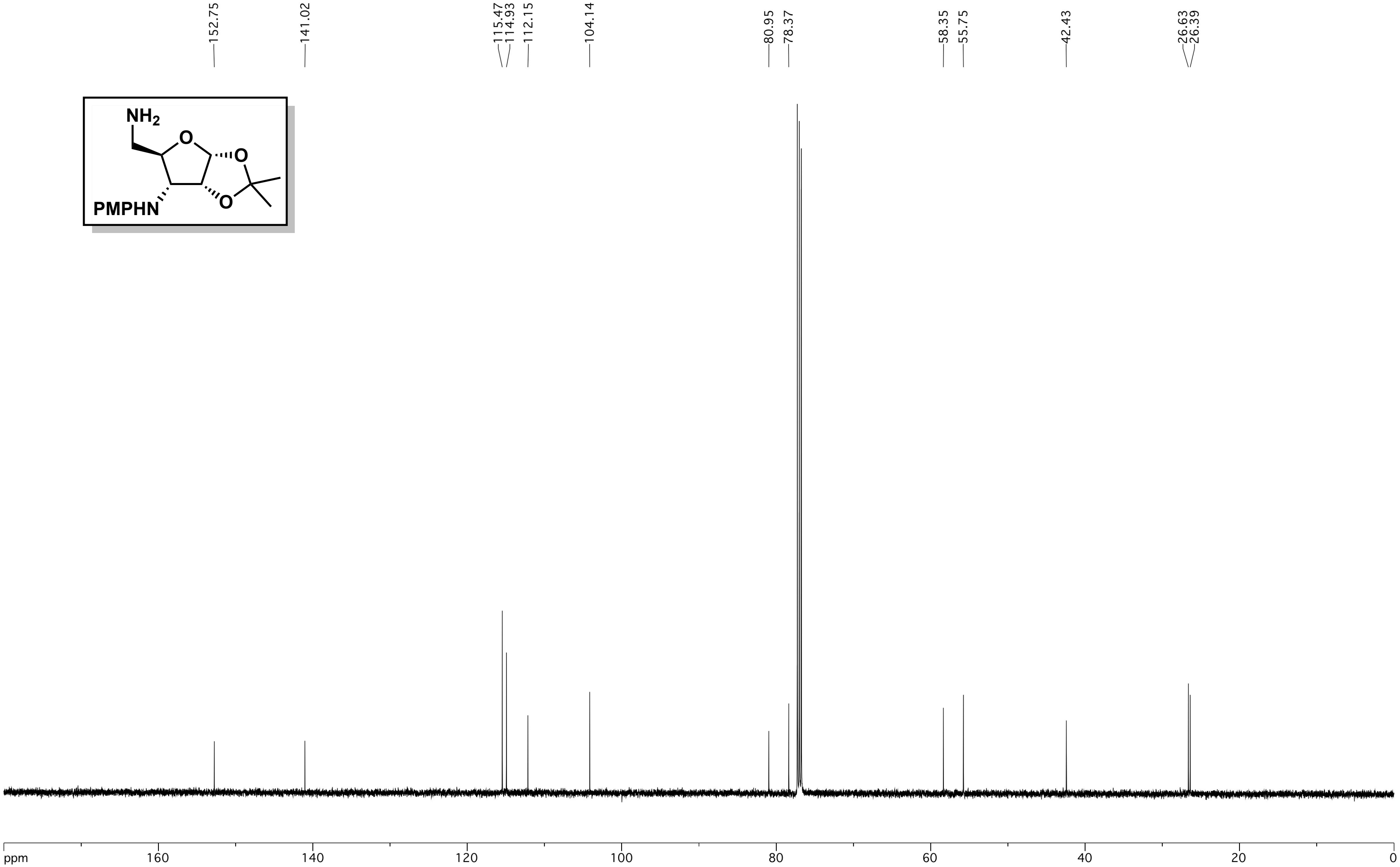

Compound **7** FTIR

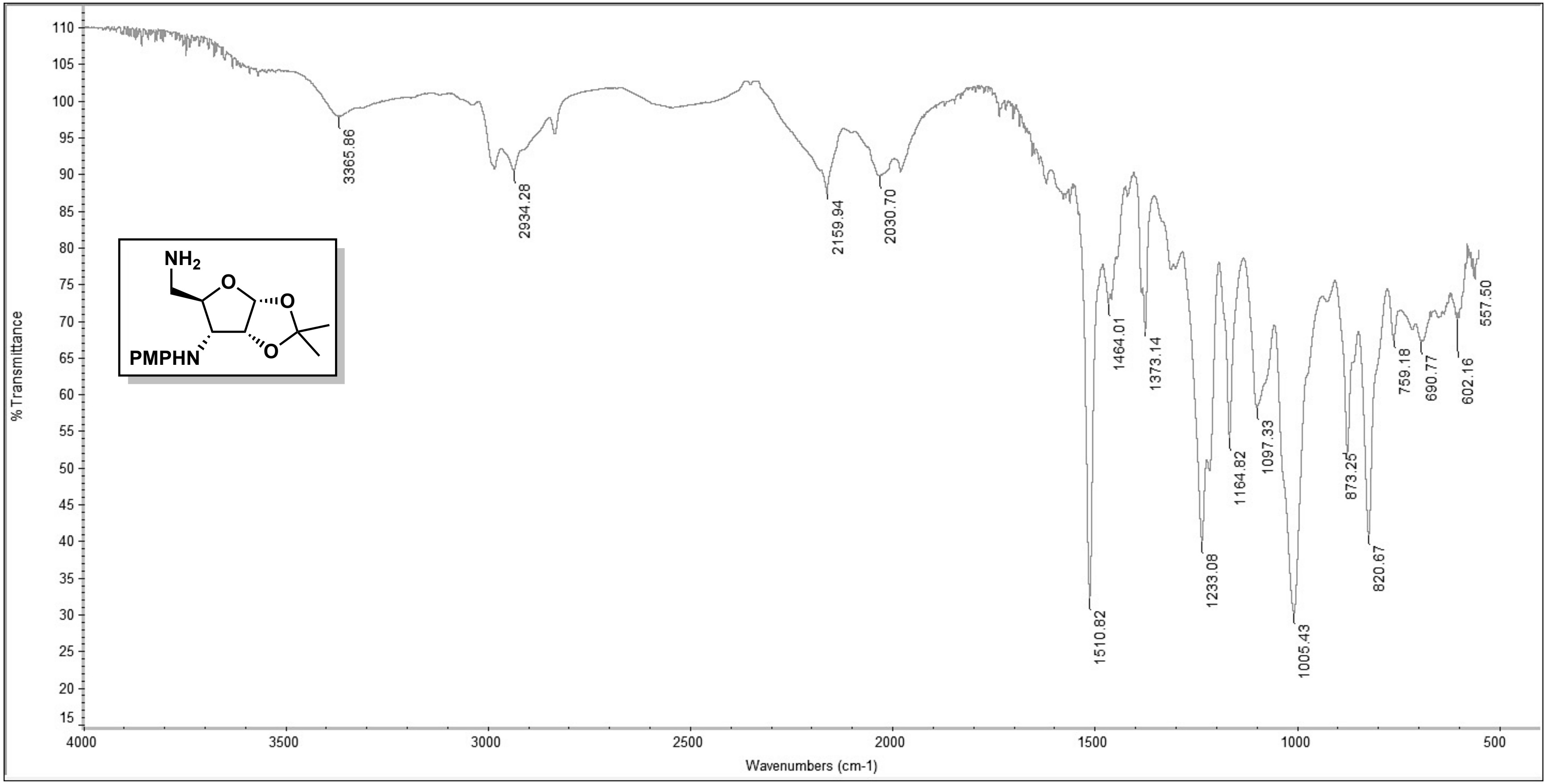

Compound **8b** ^{1}H NMR ($CDCl_3$)

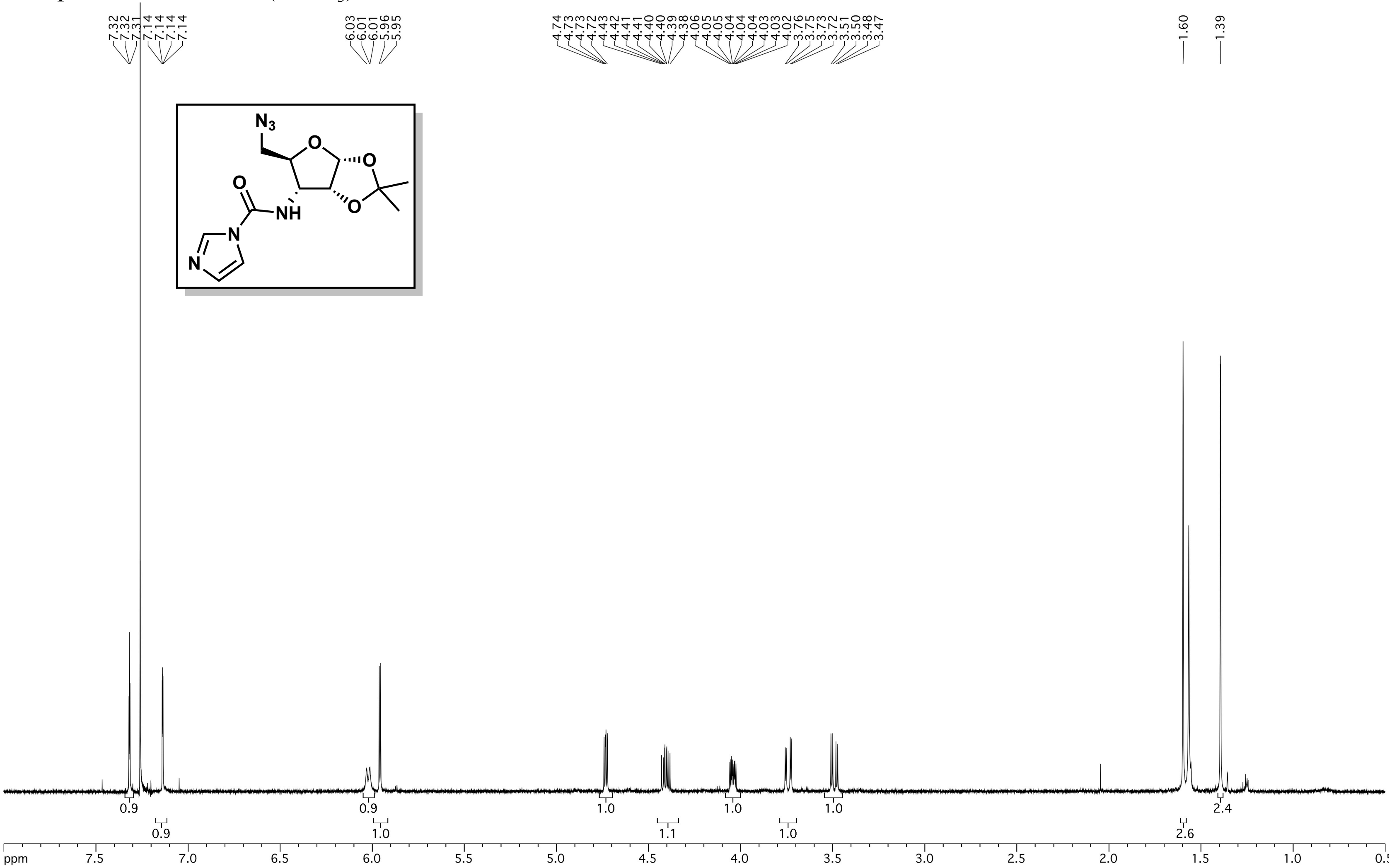

Compound **8b** ^{13}C NMR ($CDCl_3$)

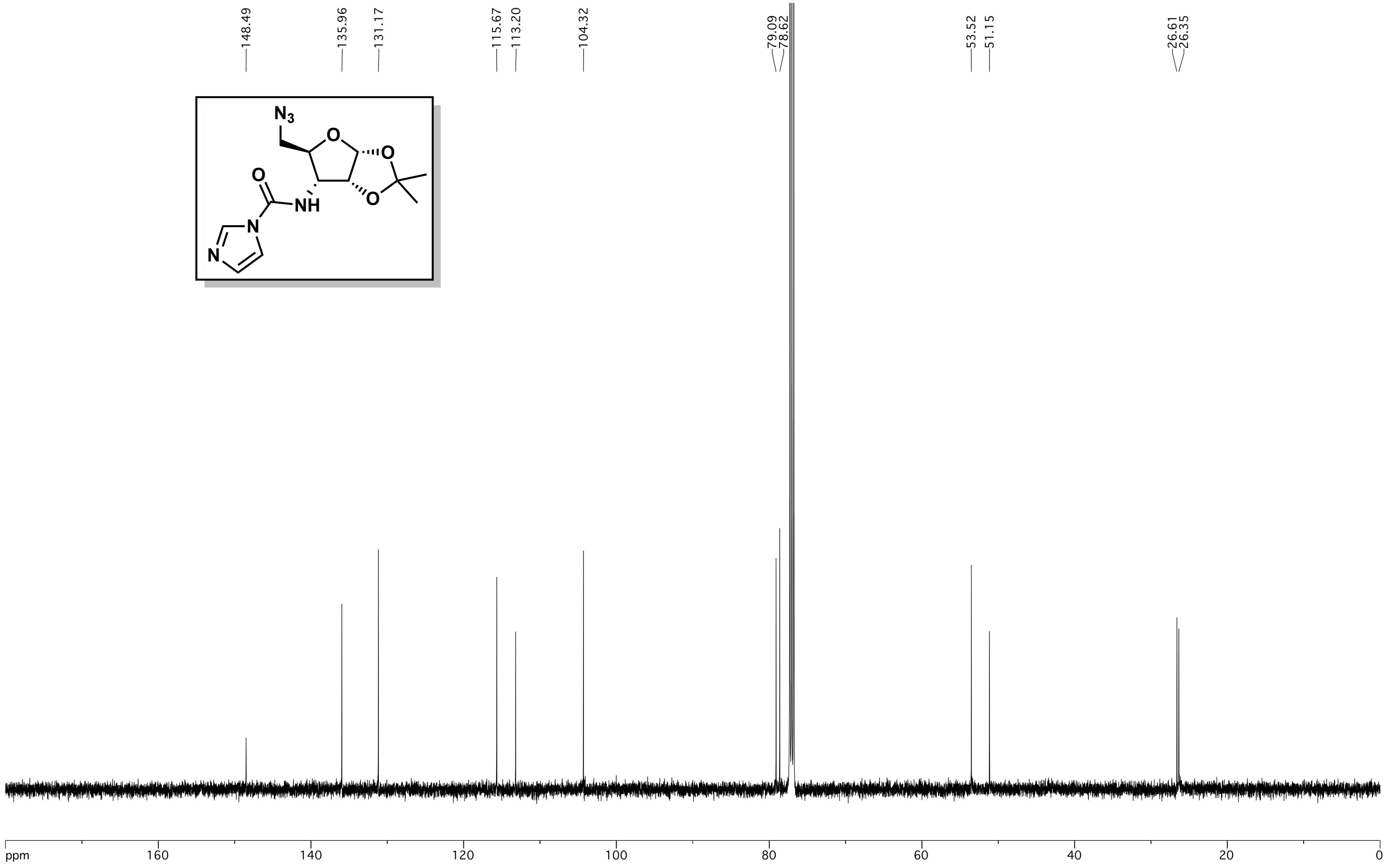

Compound **8b** FTIR

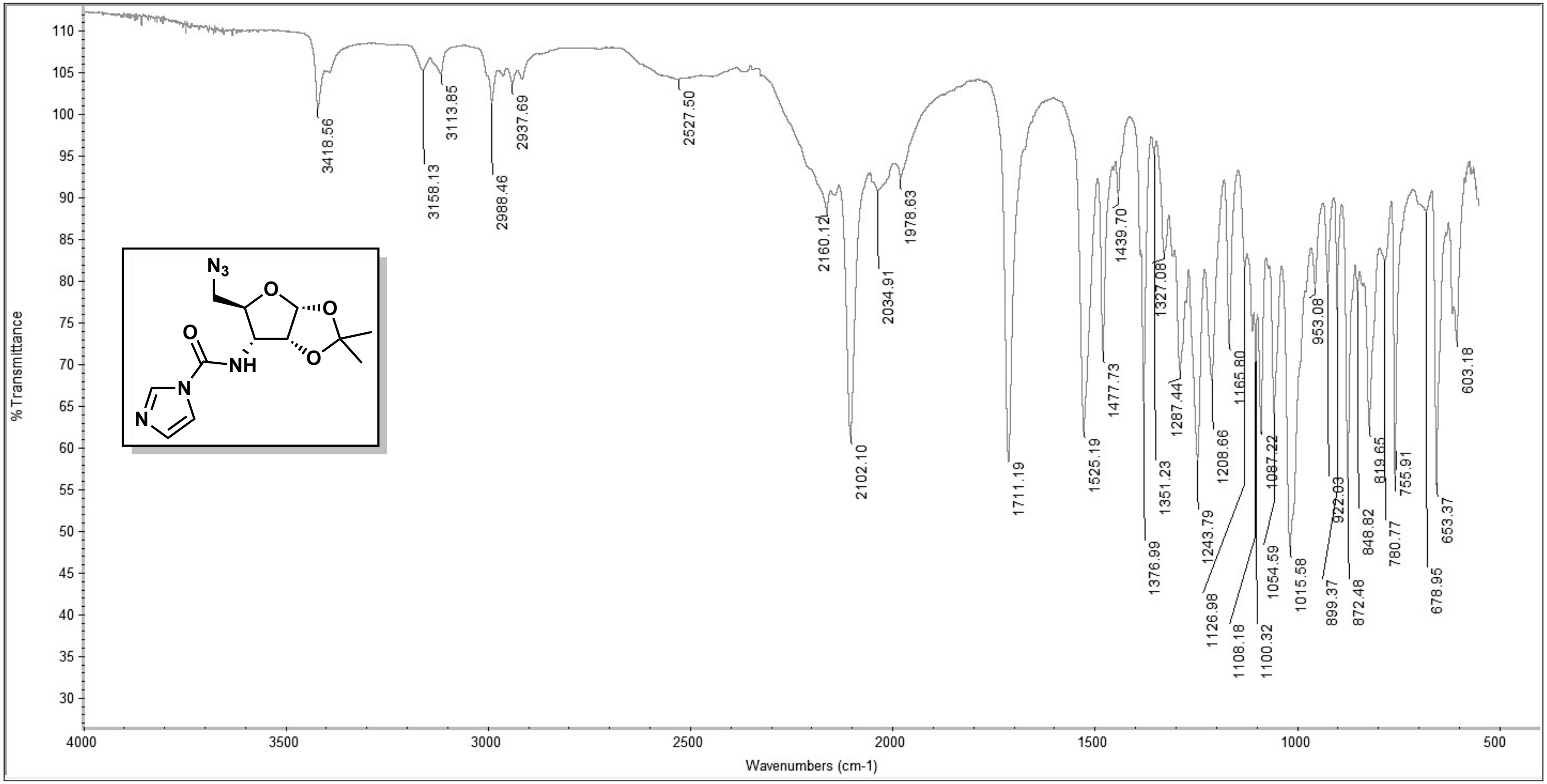

Compound **9b** ^{1}H NMR ($CDCl_3$)

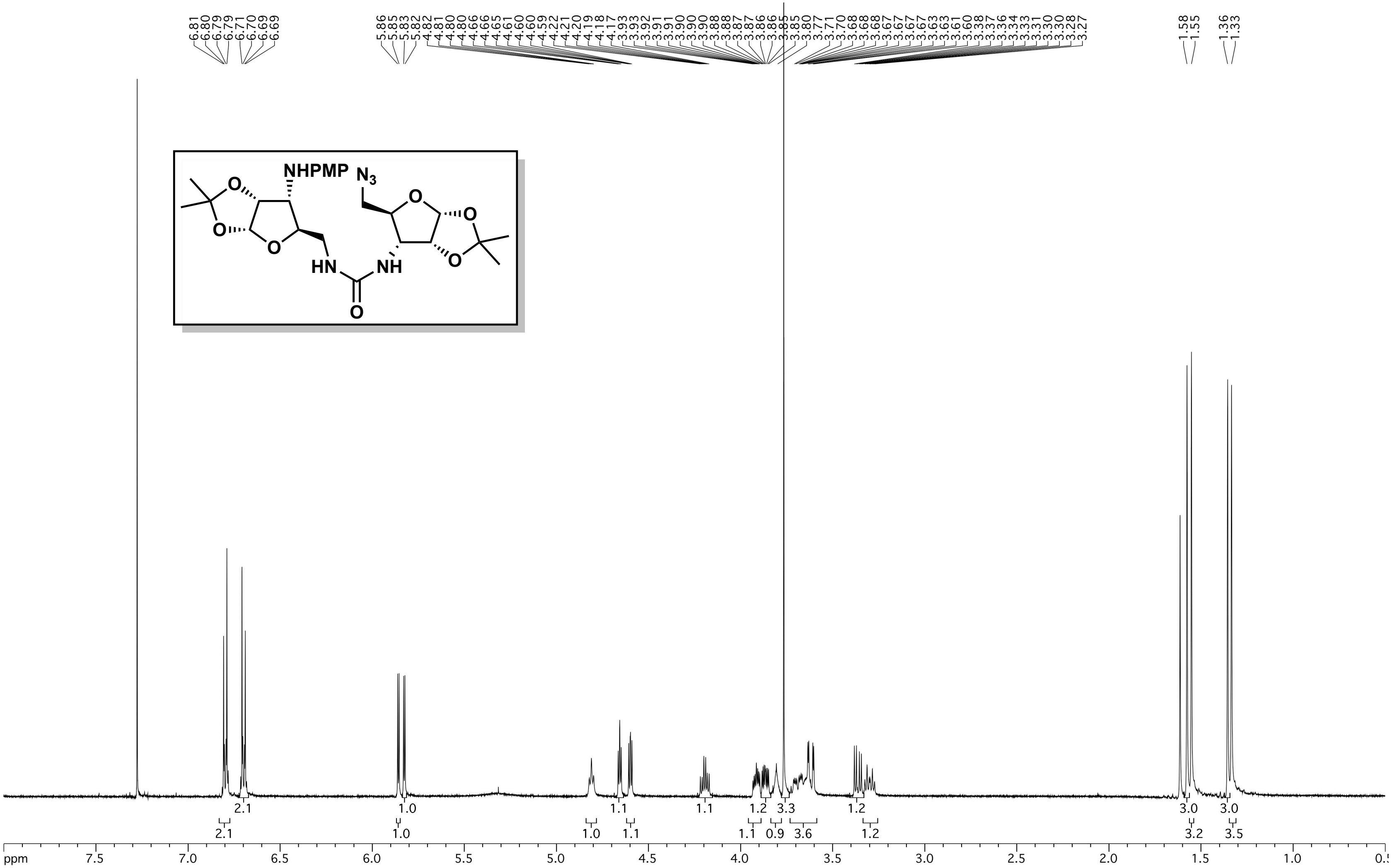

Compound **9b** ^{13}C NMR ($CDCl_3$)

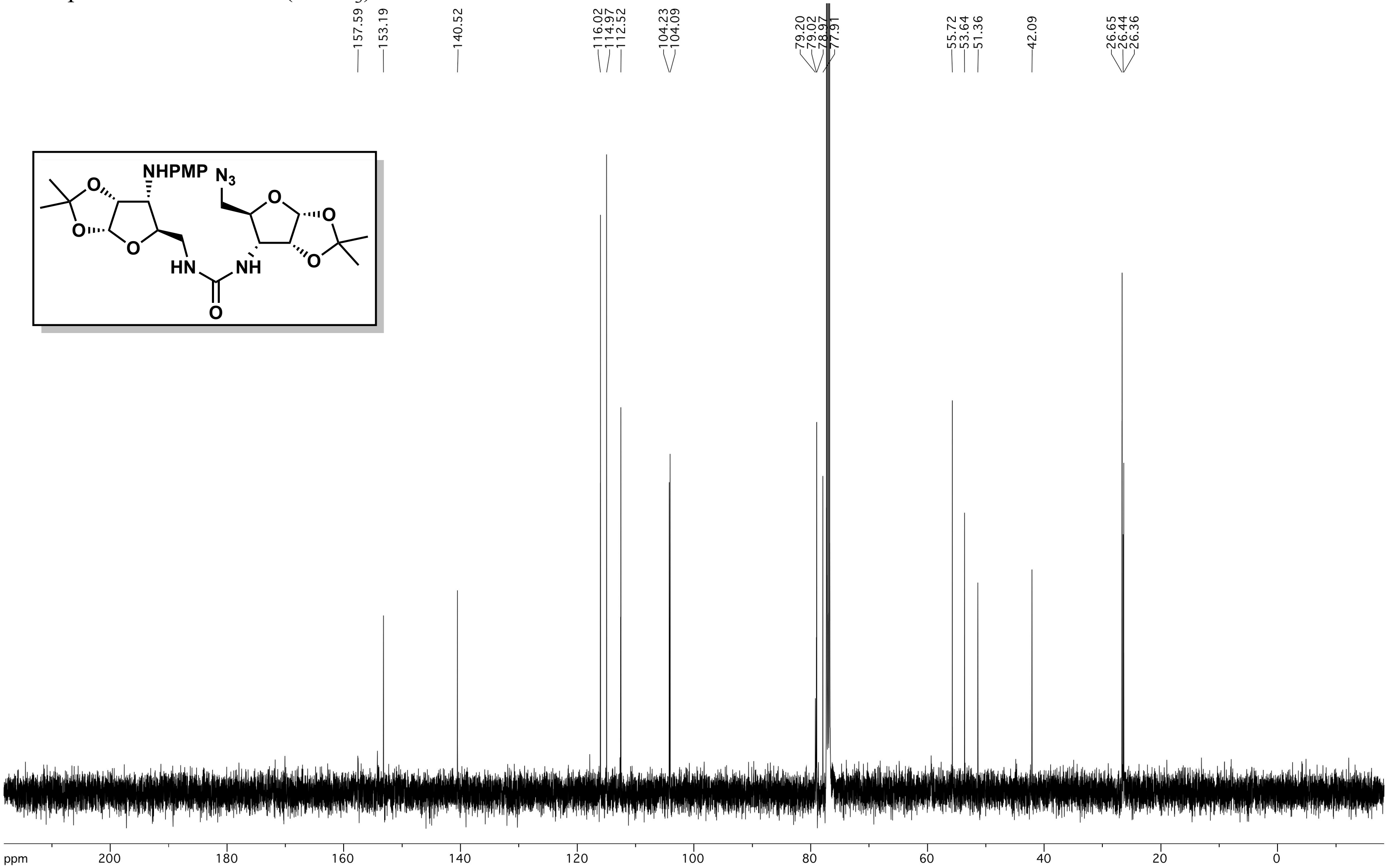

Compound **9b** FTIR

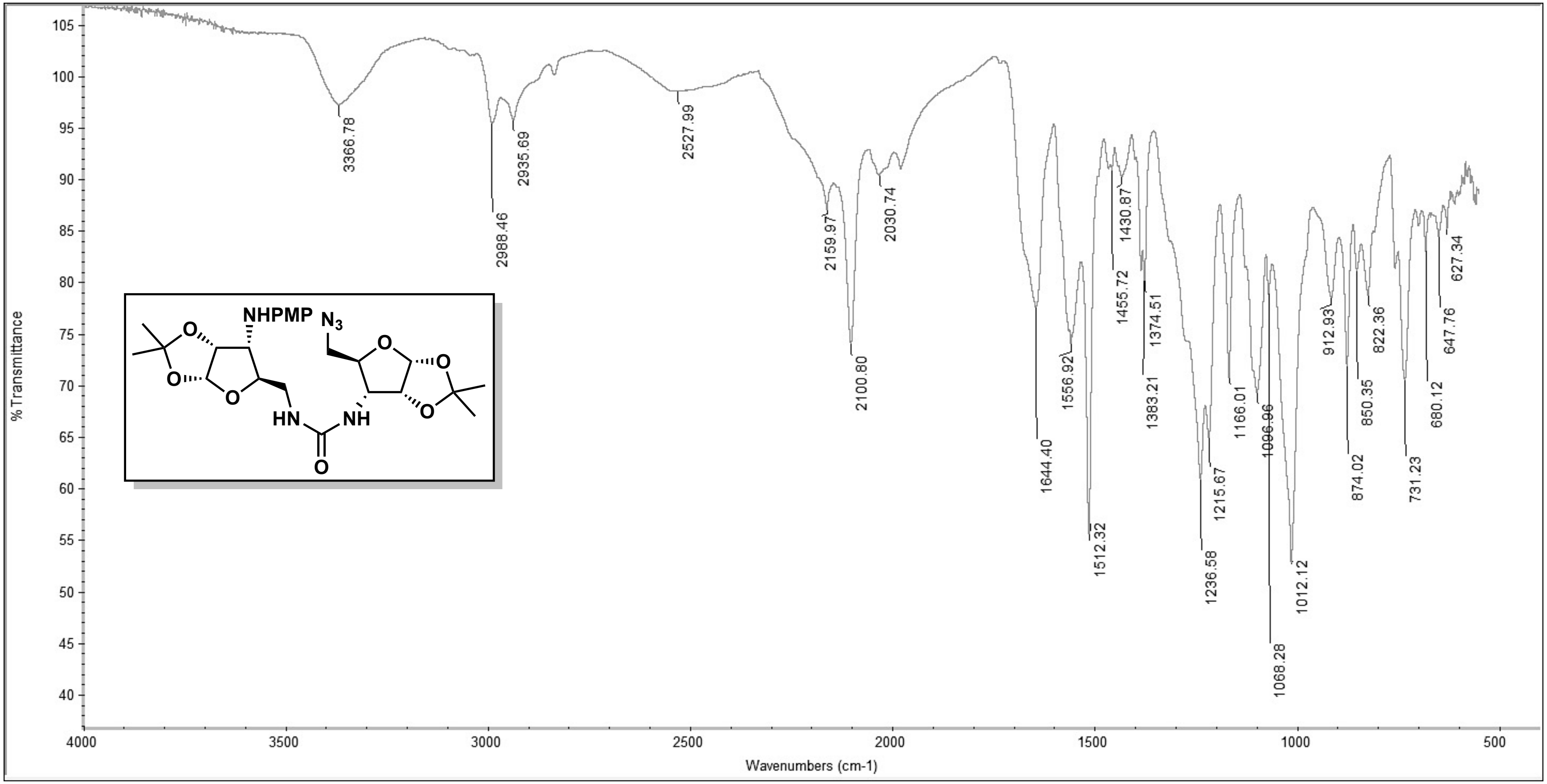

Compound **10b** ^{1}H NMR ($CDCl_3$)

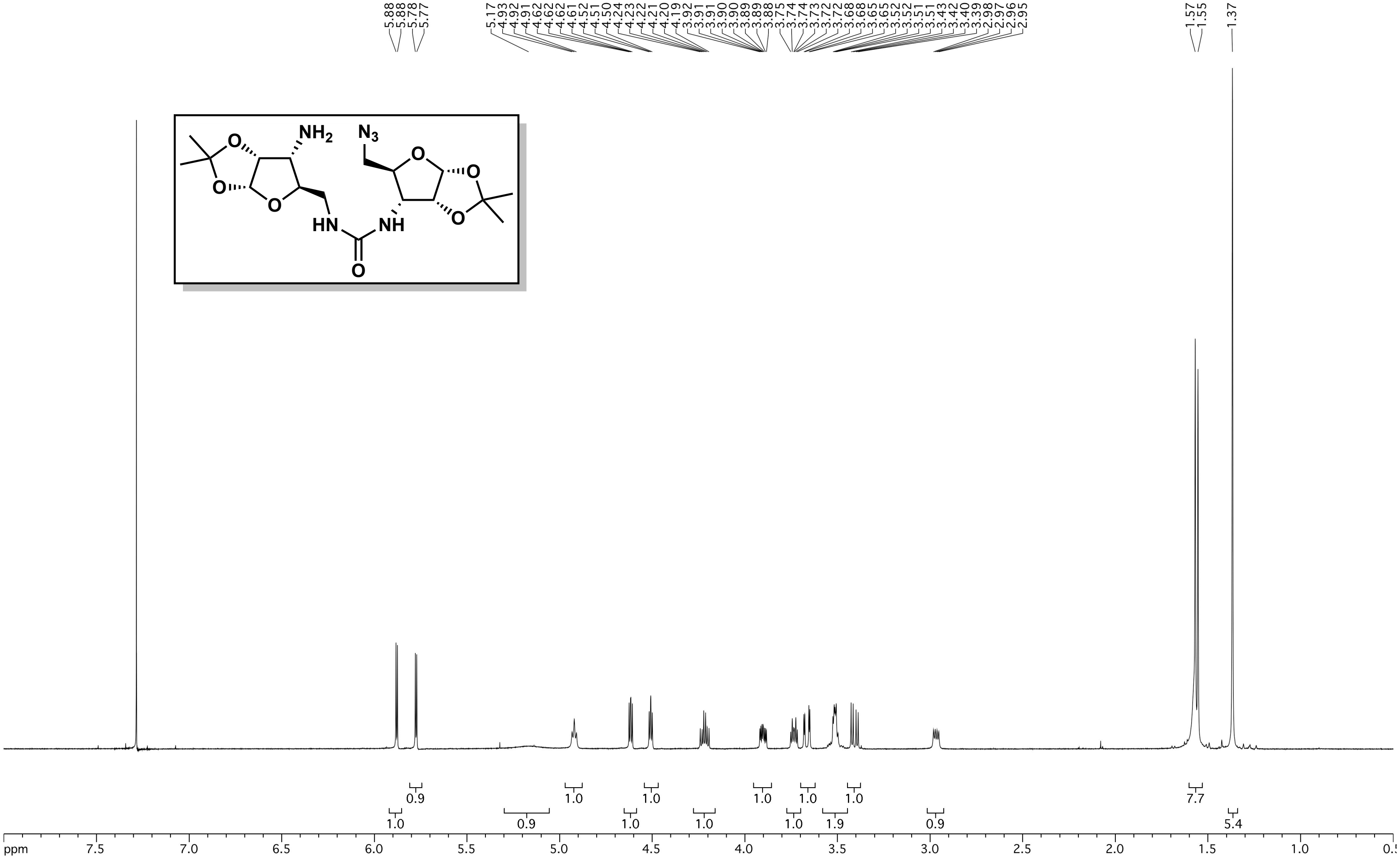

Compound **10b** ^{13}C NMR ($CDCl_3$)

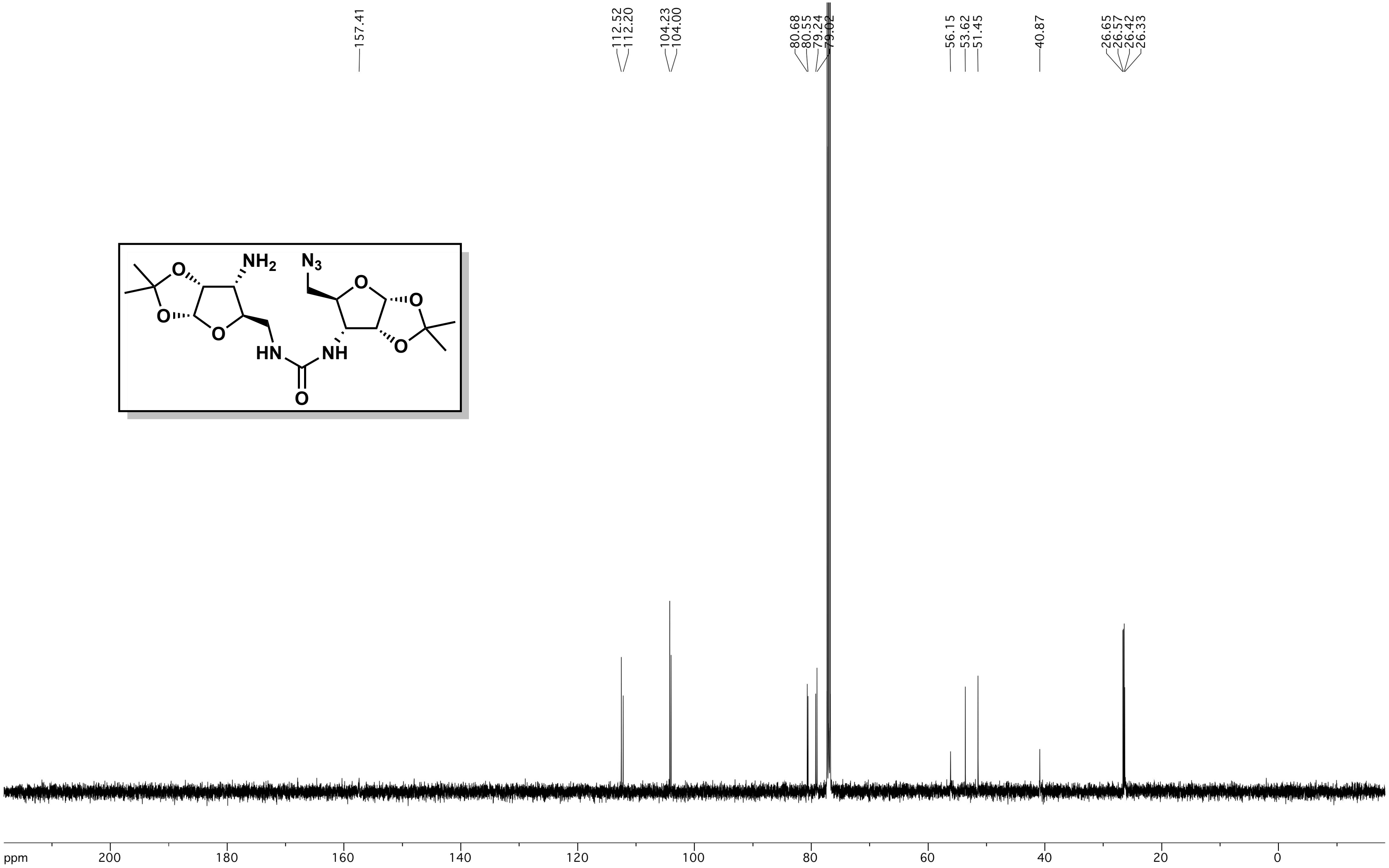

Compound **10b** FTIR

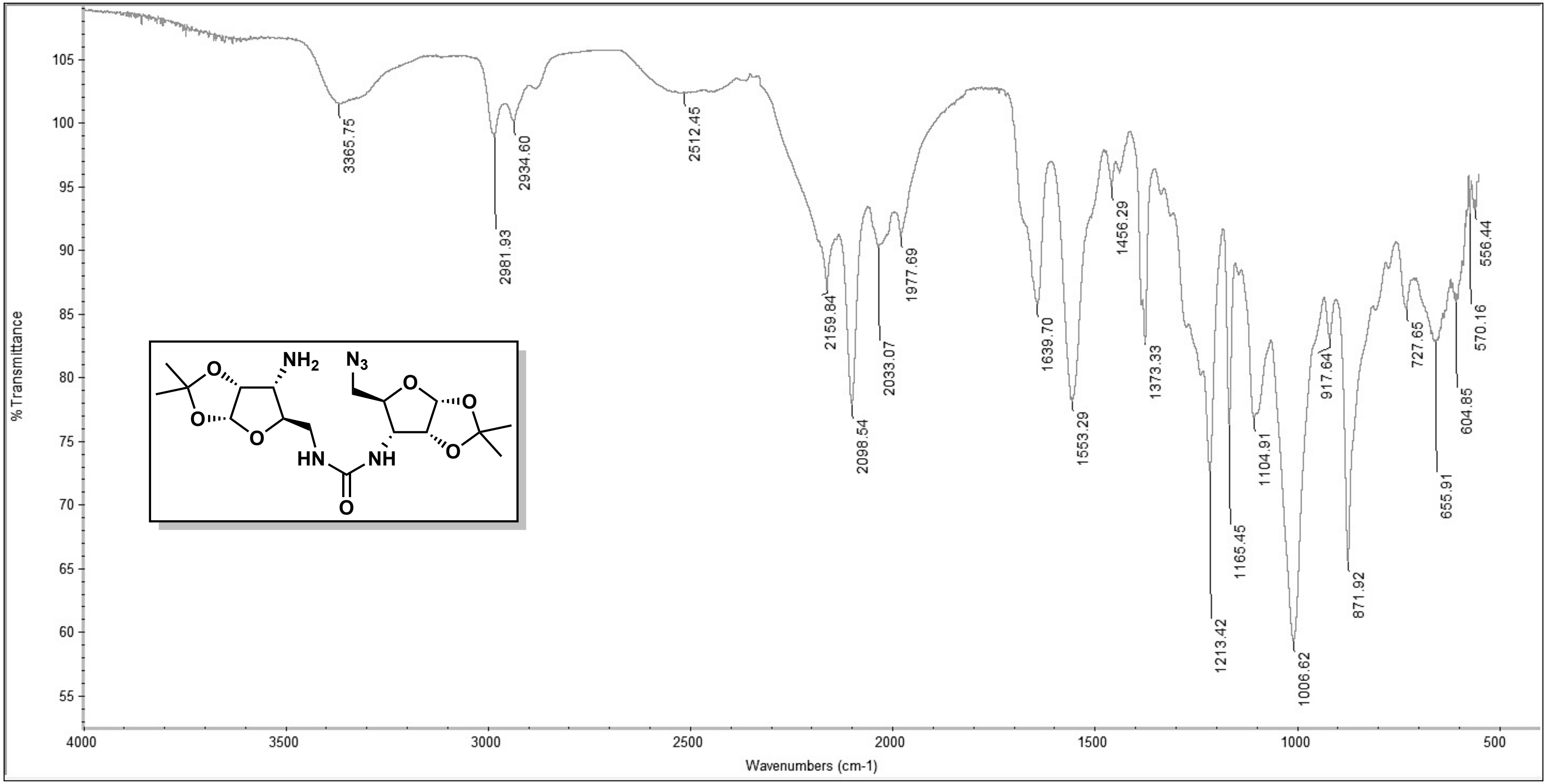

Compound **11b** ^{1}H NMR ($CDCl_3$)

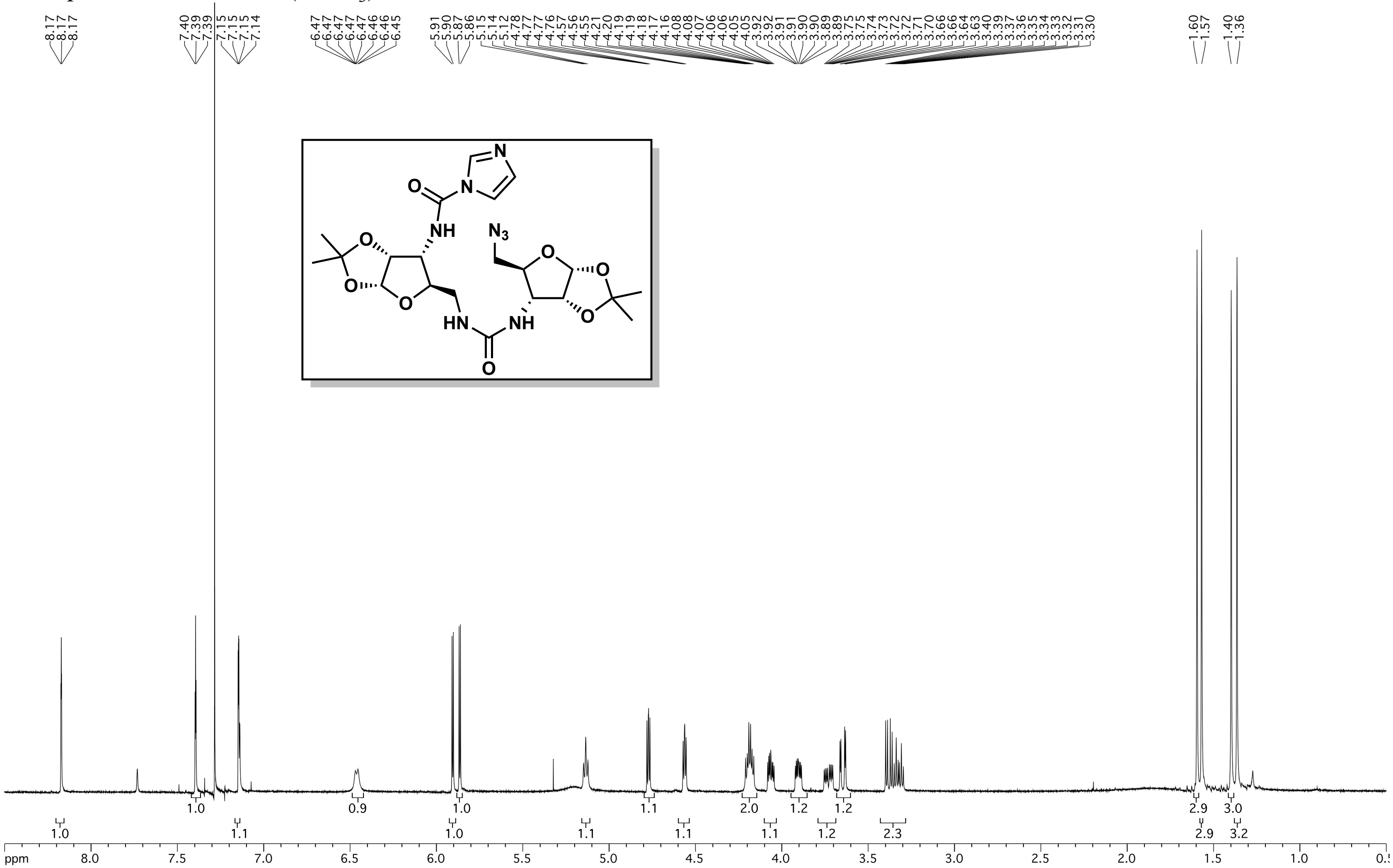

Compound **11b** ^{13}C NMR ($CDCl_3$)

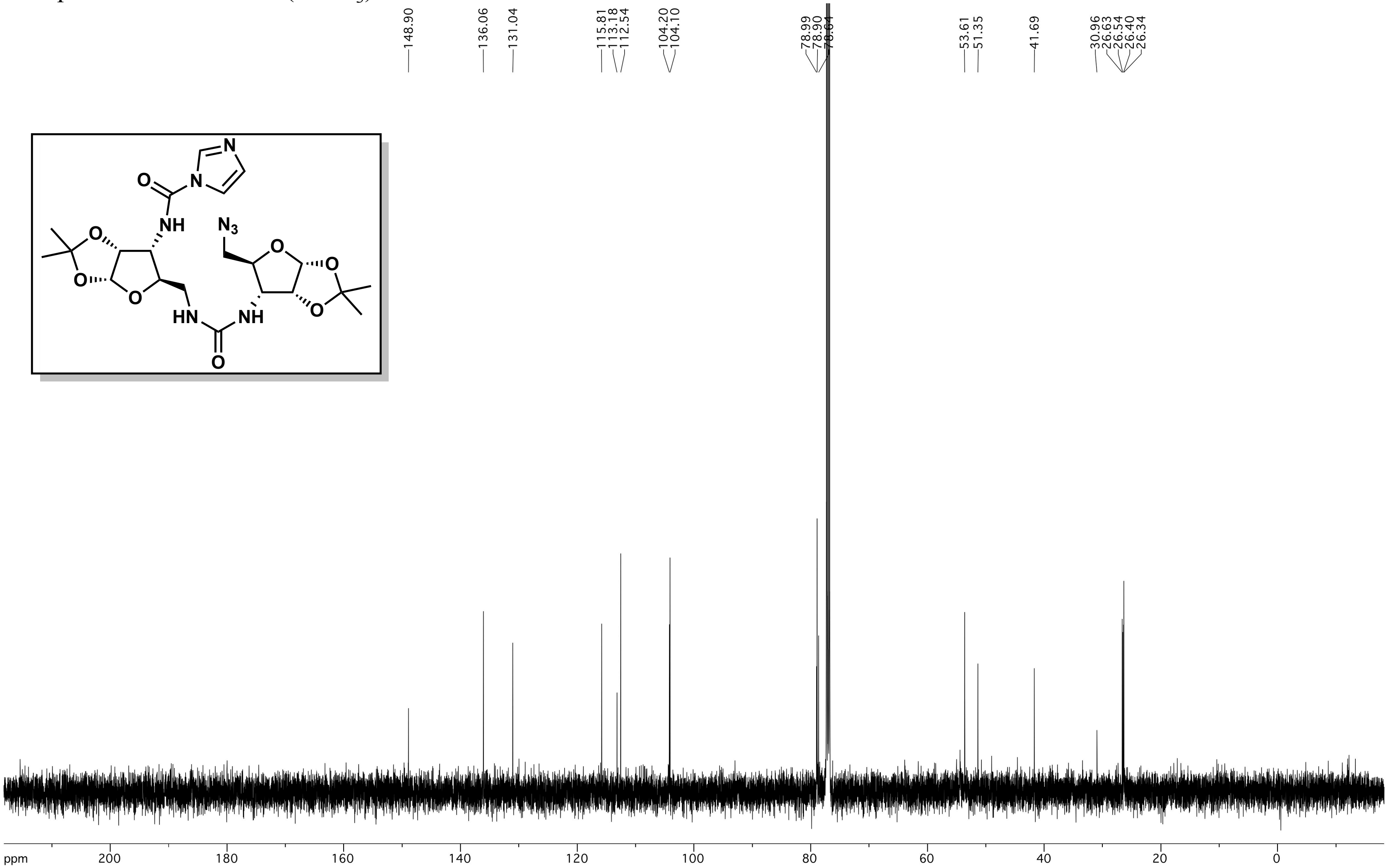

Compound **11b** FTIR

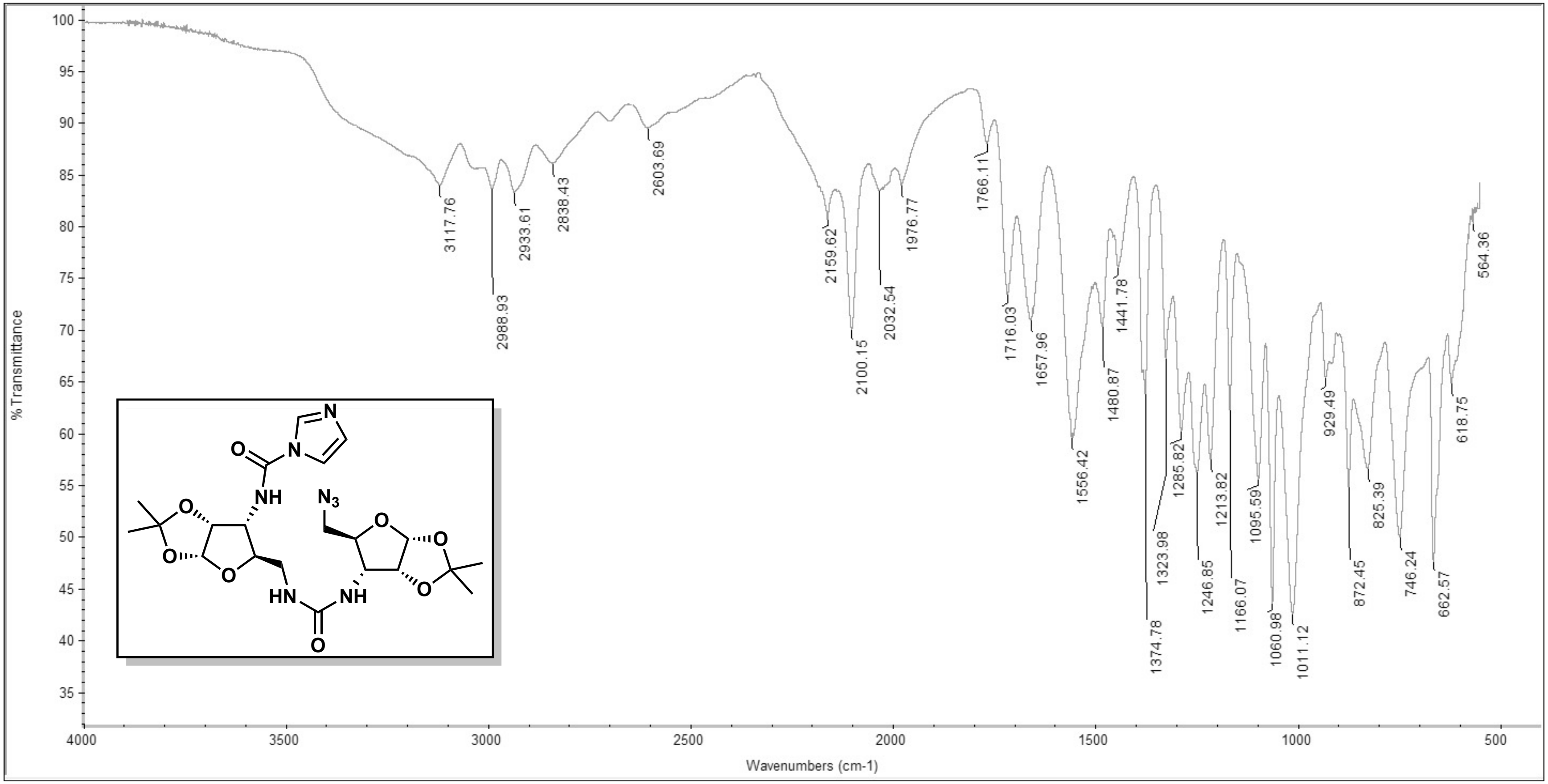

Compound **13b** ^{1}H NMR ($CDCl_3$)

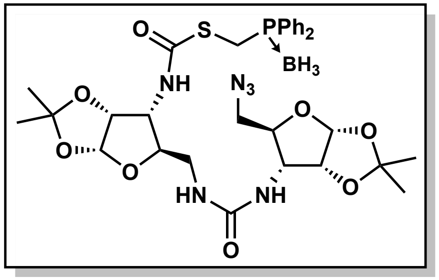

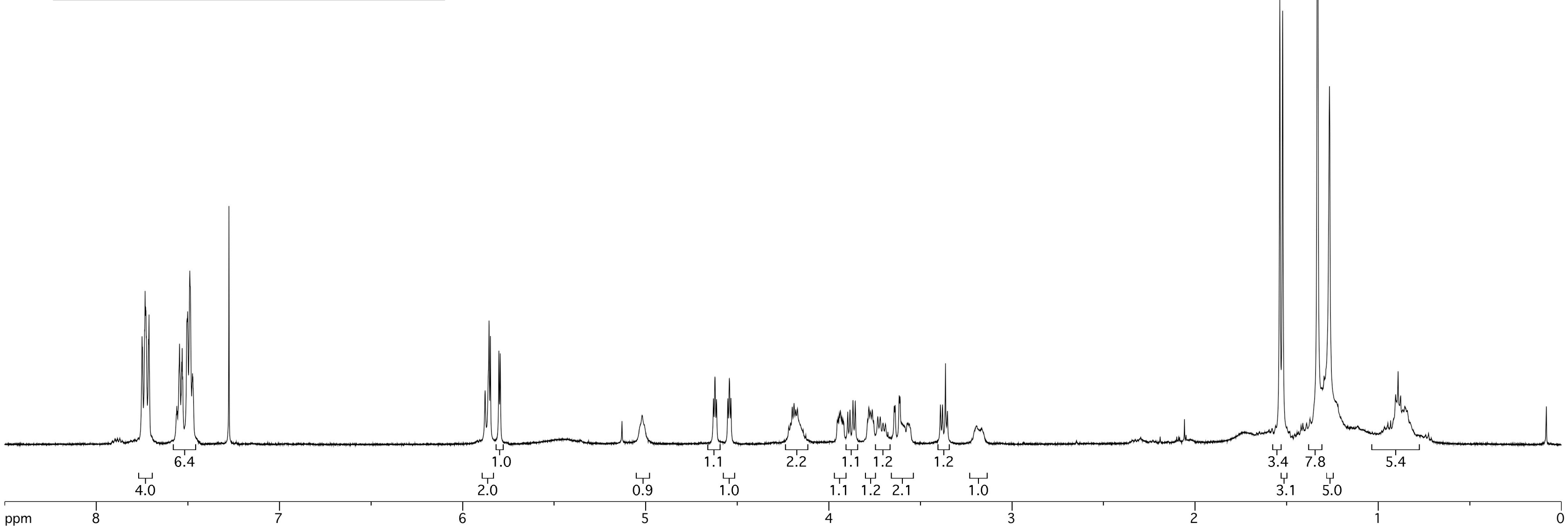

Compound **13b** ^{13}C NMR ($CDCl_3$)

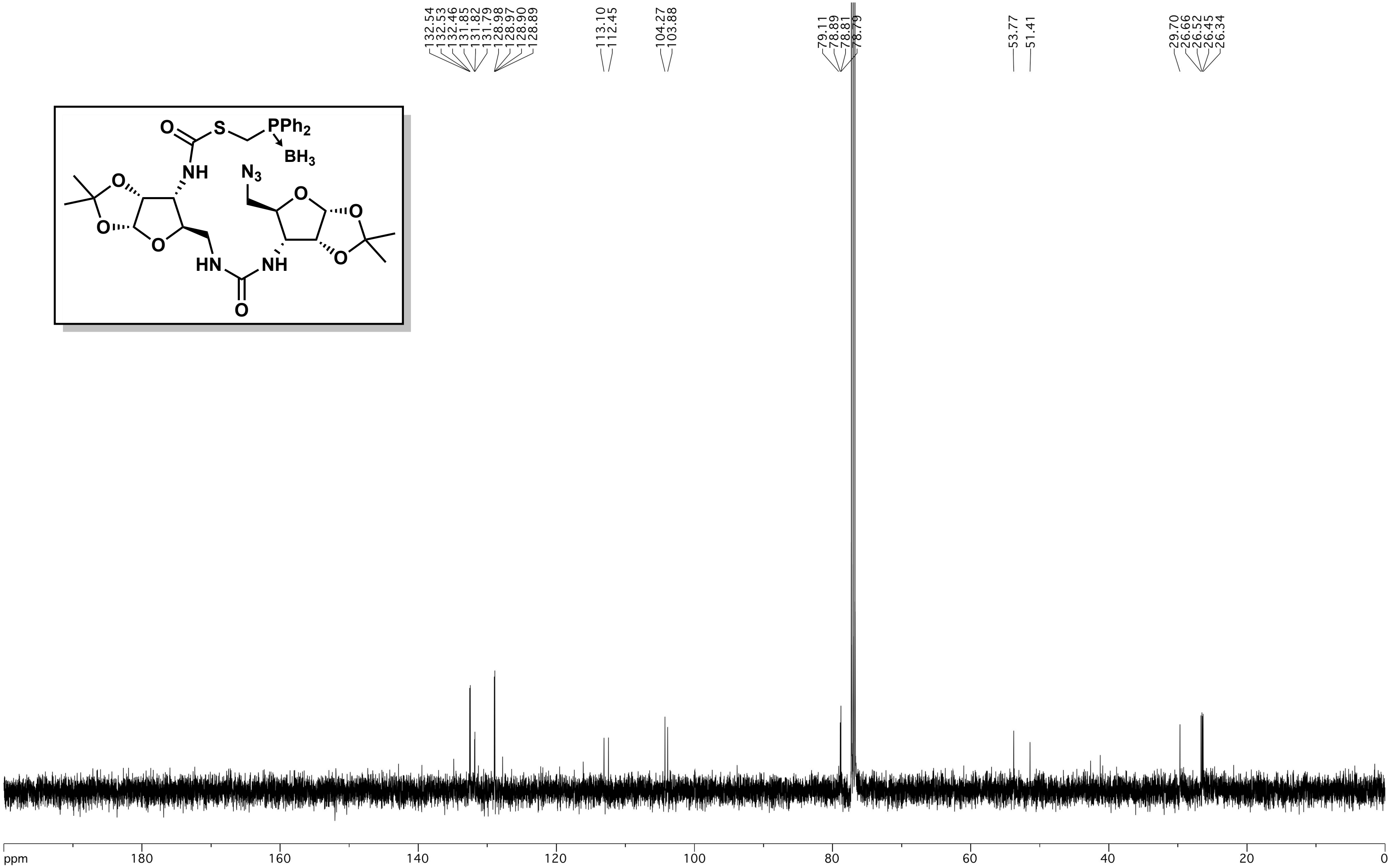

Compound **13b** FTIR

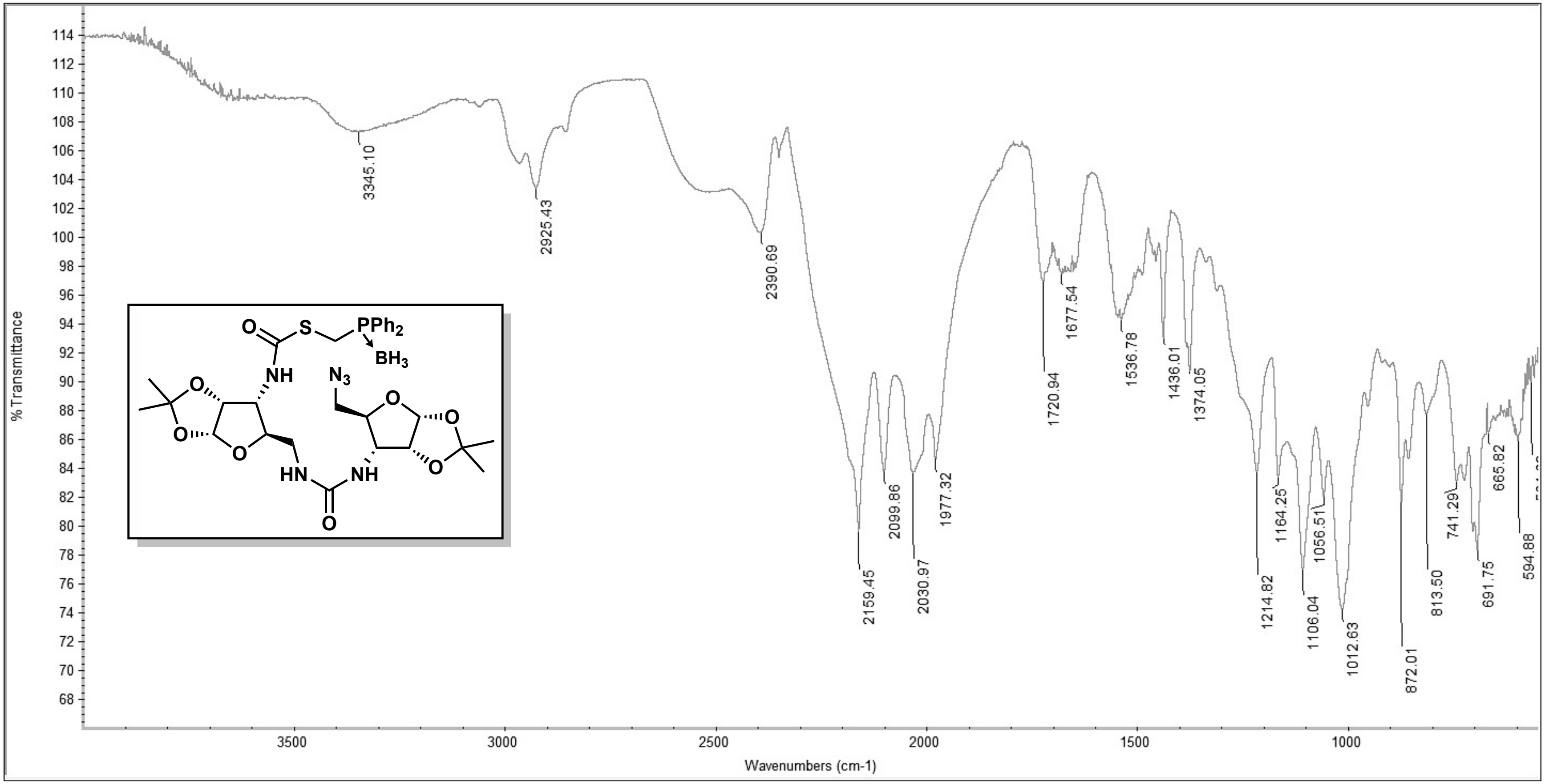

Compound **15b** ^1H NMR ($CDCl_3$)

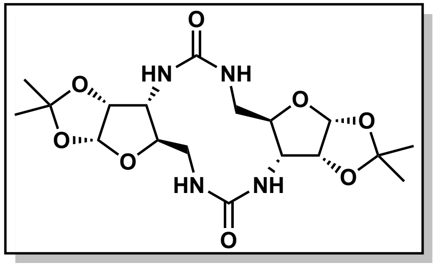

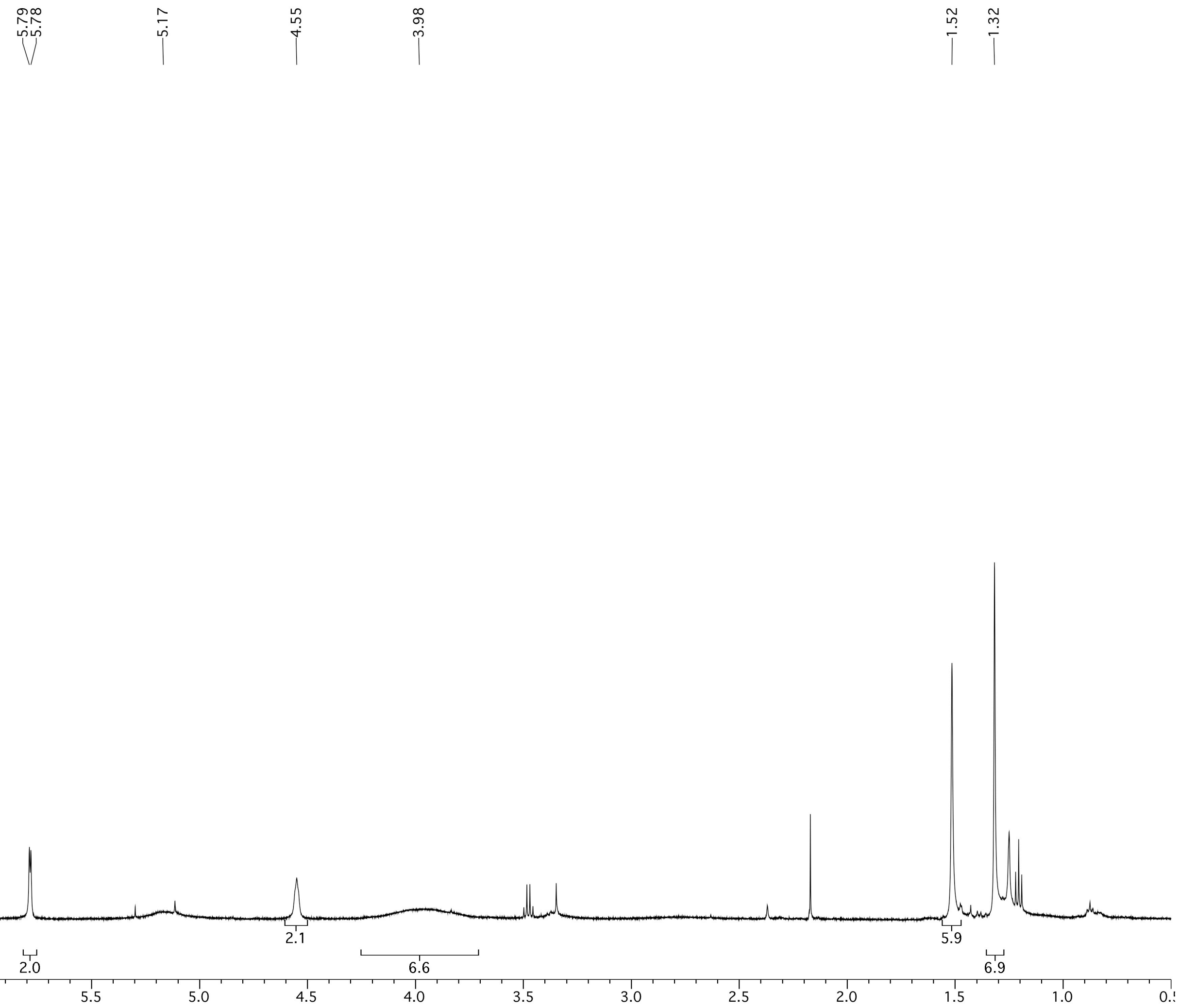

Compound **15b** ^{13}C NMR ($CDCl_3$)

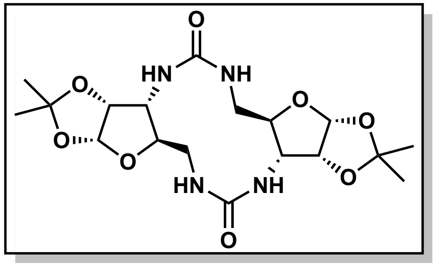

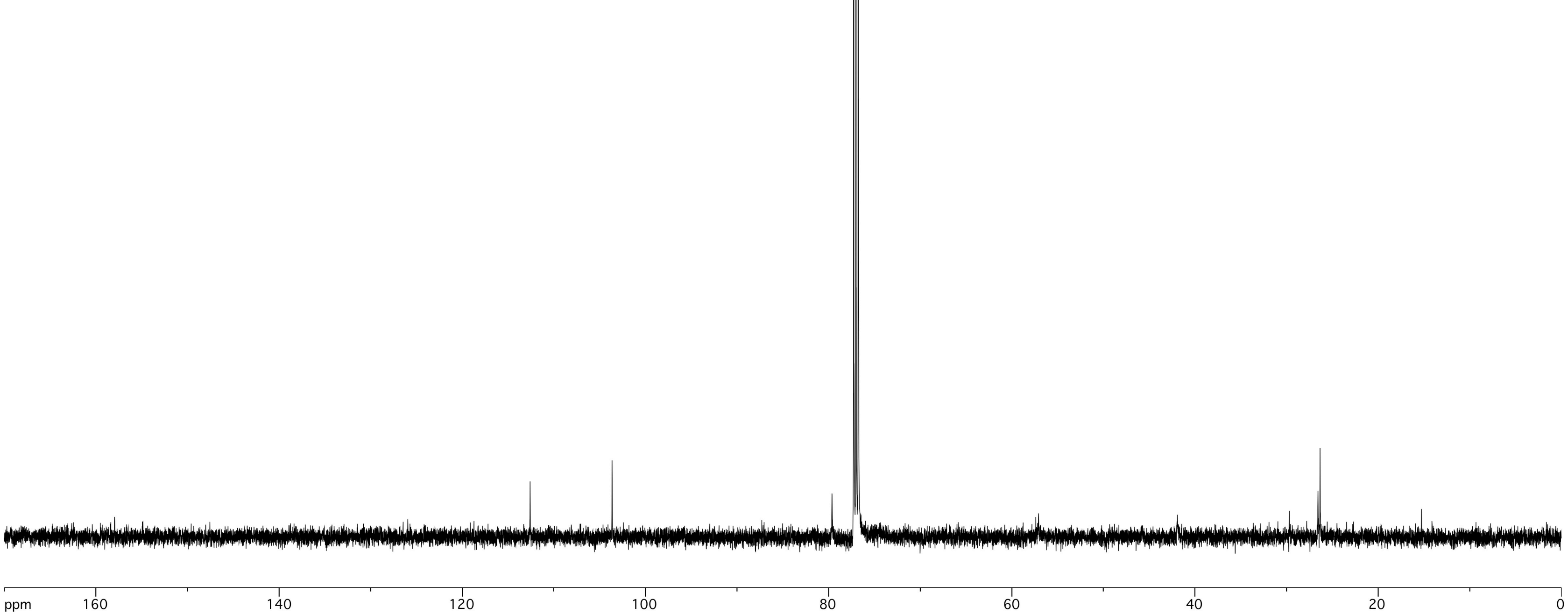

Compound **15b** FTIR

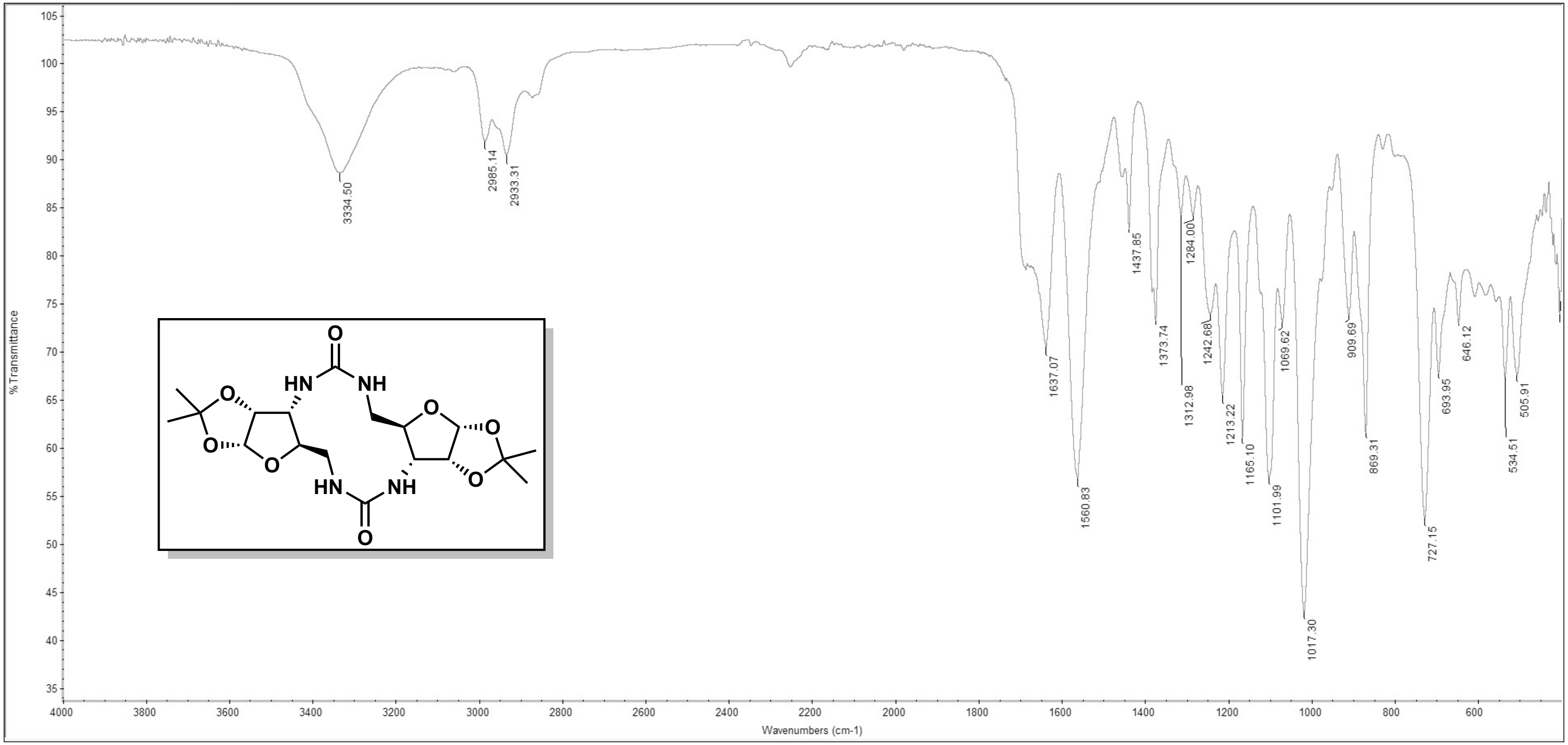

Supplement: Supplementary file 1 [file RA-007-C7RA06045A-s001.pdf]
